# Harnessing Organopotassium Reagents for Cross-Coupling with YPhos-Pd Catalysts: Opportunities, Applications, and Challenges

**DOI:** 10.1021/jacs.4c18073

**Published:** 2025-02-02

**Authors:** Daniel Knyszek, Julian Löffler, David E. Anderson, Eva Hevia, Viktoria H. Gessner

**Affiliations:** † Inorganic Chemistry II, Faculty of Chemistry and Biochemistry, Ruhr-University Bochum, Universitätsstraße 150, 44801 Bochum, Germany; ‡ Department für Chemie, Biochemie und Pharmazie, Universität Bern, Freiestrasse 3, 3012 Bern, Switzerland

## Abstract

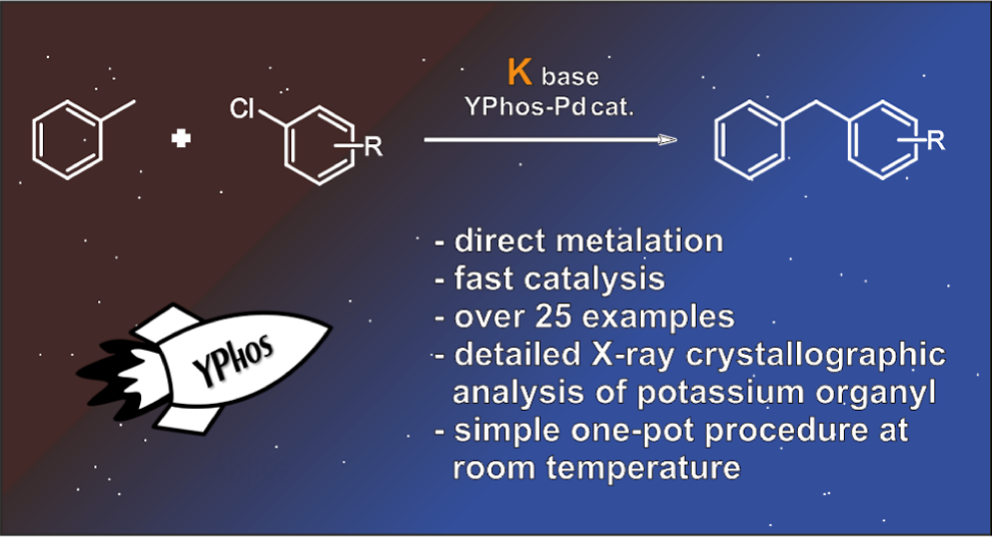

With advances in the applications of earth-abundant organopotassium reagents in C–C bond forming processes, this study pioneers Pd-catalyzed cross coupling reactions between aryl halides and a range of aryl and benzylpotassium species generated by direct C–H metalation. Key for the success of this approach is the use of electron-rich ylide-substituted phosphine (YPhos) ligands, which enable fast conversion of the potassium species in solution. This protocol can be carried out in a one-pot manner at room temperature, without the need for purification of the in situ prepared organopotassium compounds or any additional additives, enabling the isolation of a broad scope of coupling products even on a gram-scale.

## Introduction

Alkali-metal organyls especially organolithium reagents^1,2^ are among the most important organometallic reagents in organic synthesis. They are frequently employed as strong base/metalation reagents or for C–C bond formation reactions with unsaturated electrophiles such as carbonyl compounds. Despite the usually high efficiency of these transformations, the direct use of alkali-metal organyls in transition-metal catalyzed cross-coupling reactions still remains extremely limited.^3^ This contrasts with the coupling reactions that use organoboronic acids (Suzuki),^4^ organozinc (Negishi),^5^ tin (Stille),^6^ and magnesium reagents (Kumada),^7^ which have been significantly improved over the years and are widely applied, not only in academia but also in many industrial processes.^8−11^ While couplings using alkali-metal organyls in palladium catalysis can be envisaged as a highly appealing, atom economical approach, since many of these reagents can be accessed by direct C–H metalation of the organic substrate with an alkali-metal base,^12,13^ practically their use can be very challenging. This is primarily attributed to the high reactivity of organo-alkali reagents, which frequently results in unwanted side-reactions such as homocoupling or dehalogenation and low functional group tolerance.^14^ Moreover, selectivity issues are particularly severe for the more ionic heavier alkali-metal reagents, which notoriously suffer from fast degradation in many common organic solvents such as diethyl ether or THF.^15,16^ Accordingly, the few advances that have been made in direct coupling of organo-alkali compounds mostly focused on the use of lithium.^17−19^

**Figure 1:**
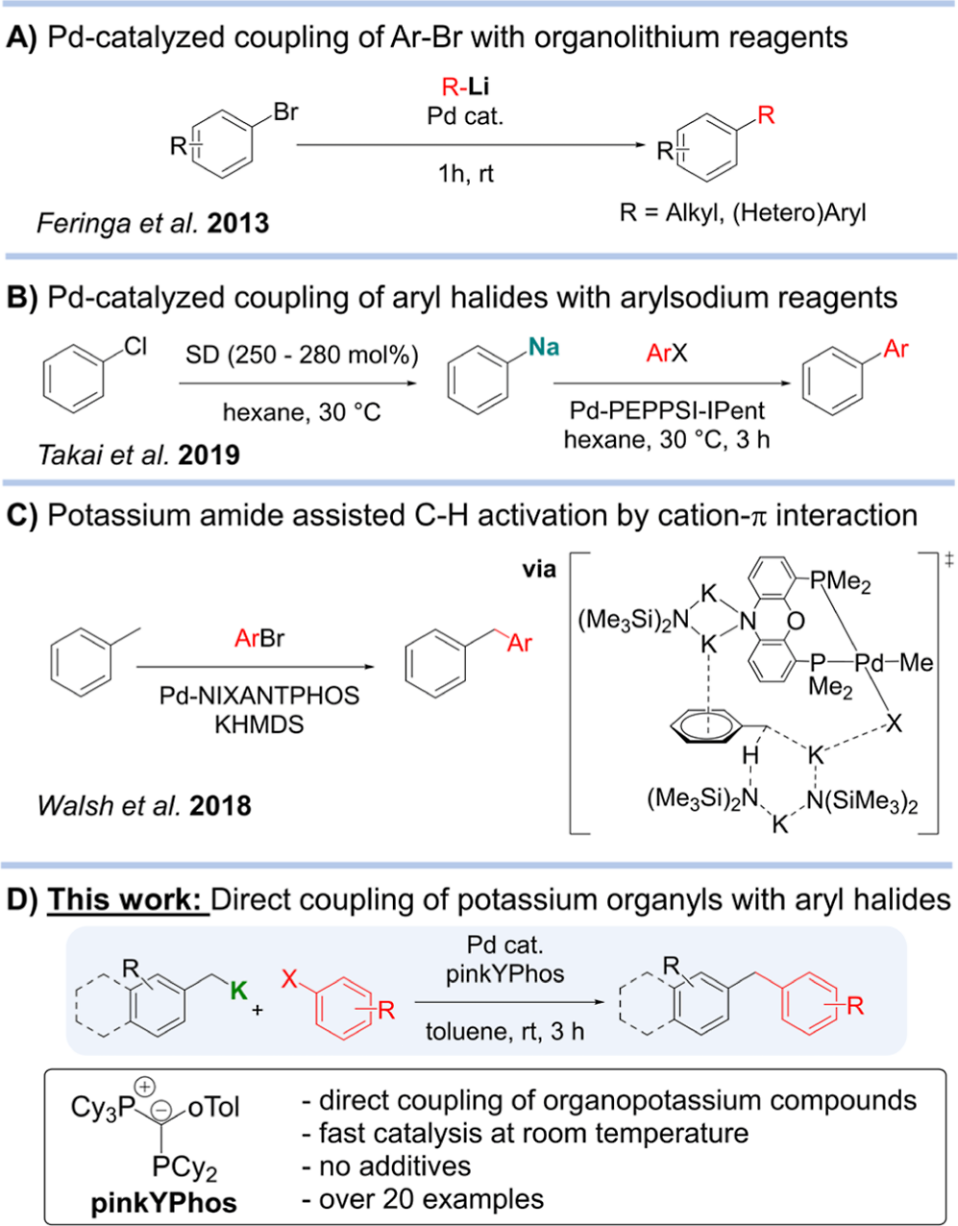
Cross-couplings of polar organometallic reagents.

The direct coupling of organolithium compounds was first reported by Murahashi in the 1970s^20,21^ but not applied for more than 30 years due to the poor selectivity. Significant progress was made by Feringa and co-workers in 2013, utilizing palladium catalysts with electron-rich phosphines or carbenes, which enabled the coupling of a series of aryl and alkyllithium compounds with aryl halides at room temperature (Figure 1A).^22−24^ This reaction was recently further improved by our group applying our highly electron-rich ylide-functionalized phosphines (YPhos),^25,26^ which allowed the challenging sp^3^-sp^2^ coupling of alkyllithiums with aryl chlorides.^14^

Despite their still limited functional group tolerance, these protocols represent valuable additions to other coupling reactions, since they allow the synthesis of important starting materials from commercially available organolithium or aryllithium compounds, conveniently synthesized by directed-ortho-metalations or via Li-halogen exchange protocols.^27^ While direct metalation protocols can be regarded as more appealing, since they do not require the use of prefunctionalized halogen-substituted substrates, direct lithiation of nonactivated substrates (in terms of p*K*_a_) can be particularly challenging. Typified by the Lochmann-Schlosser super base,^28,29^ heavier group 1 metal reagents have shown greater metalation power than organolithium reagents.^30−32^ This has been reported already as early as the 1960s, and since then further remarkable advances have been made by Knochel,^3,4^ Hevia,^33^ Mulvey,^34^ and others in the development of organosodium and organopotassium bases, broadening the scope of direct C–H metalations. Given these advances, the direct cross-coupling of these heavier organoalkali compounds is highly desirable. Furthermore, given the significantly greater abundance of sodium and potassium compared to lithium in the Earth’s crust, coupled with the ever-growing demand for lithium in battery technologies, such a protocol could provide a valuable alternative to mitigate potential resource scarcity.^35,36^

In 2019, Takai et al. reported the first protocol for the coupling of aryl halides with organosodium reagents formed by halogen-sodium exchange using a commercially available sodium dispersion (Figure 1B).^19^ Despite this initial breakthrough, the protocol still primarily required aryl halides as starting materials for generating the relevant sodium aryl intermediate, which often necessitated an additional transmetalation step to zinc or boron, prior to the palladium-catalyzed cross coupling step. Organopotassium reagents have also shown some promise in coupling reactions. Thus, insightful studies by Walsh have shown that KHMDS (HMDS = N(SiMe_3_)_2_) in combination with a Pd phosphine complex can promote the deprotonative activation of toluene and diarylmethane derivatives enabling their cross-coupling with aryl bromides (Figure 1C).^37,38^ Mechanistic investigations hint at a critical role for potassium in facilitating the activation of toluene via K-arene π-interactions. However, these sp^2^-sp^3^ couplings with aryl bromides required high temperatures (110 °C) for the less activated toluene derivates and was not extended to the more widely available aryl chlorides. More recently, Newman et al. demonstrated the use of the Lochmann-Schlosser superbase or organolithium reagents to generate benzylpotassium or lithium intermediates by direct Csp^3^–H metalation. While compounds formed through lithiation could be directly coupled, the potassium compounds required in situ transmetalation to zinc prior to the Pd-catalyzed cross-coupling with aryl chlorides.^39^ As far as we can ascertain, no direct cross coupling of isolated organopotassium reagents has been reported as their inherently high reactivity and limited compatibility with organic solvents have proved to be difficult to tame.

Recent reports by our group have uncovered the broad applicability of our YPhos ligands to promote challenging palladium-catalyzed reactions. Owing to their strong donor ability, YPhos Pd complexes facilitate oxidative addition even of the more challenging aryl chlorides, enabling fast catalysis.^40−42^ Motivated by these precedents, here, we investigate the direct cross-coupling of heavier alkali-metal organyls with aryl chlorides. Filling a gap in the knowledge, we report the efficient synthesis of a range of organo-potassium compounds via direct C–H metalation and their subsequent application in cross-coupling reactions using a highly active YPhos palladium catalyst (Figure 1D).

## Results and Discussion

Before devising a protocol for the coupling reaction, we first sought to optimize the C–H metalation to directly access organopotassium compounds without prior functionalization of the substrates. Albeit various routes for the potassiation of C–H bonds have been reported, they have predominantly been applied to a restricted range of substrates.^28,29,33,43^ To ensure access to a larger library of compounds, we thus explored various reaction conditions initially focusing on the synthesis of benzylpotassium reagents using a CO_2_ quench to quantify the efficiency of the metalation conditions (Table 1). Initial tests using 1 equiv of the Lochmann-Schlosser base and toluene only gave the corresponding carboxylic acid **5** in 45% yield after reaction with carbon dioxide (see Supporting Information). When increasing the amount of toluene to 1.5 equiv, the yield could be increased to 65% (entry 1). Using the same reaction conditions, O’Shea’s LiTMP/KO*t*Bu (TMP = 2,2,6,6-tetramethylpiperidide) system^43^ yielded the carboxylic acid in only 34% (entry 2). This lower conversion can presumably be attributed to the absence of a directing group in our system, which was utilized in the initial report by O’Shea to direct the metalation selectivity toward the methyl group.^43^ To our delight, switching to KCH_2_TMS^44^ (TMS = trimethylsilyl) afforded **5** in 80% yield (entry 3), and a further increase to 92% could be obtained when using PMDETA (*N*,*N*,*N*′,*N*″,*N*″-pentamethyltriethylenetriamine) as an additive in the metalation (entry 4).^33^

**Table 1:**
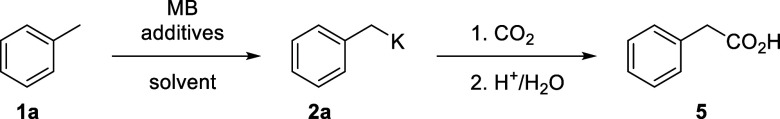
Optimization of Conditions for Direct Potassiation of Benzylic C–H Bonds^a^

entry	MB	additive	solvent	yield of **5** (%)
1	*n*BuLi + KO*t*Bu	none	hexane	65 (69^b^)
2	LiTMP + KO*t*Bu	none	THF	34
3	KCH_2_TMS	none	hexane	80
4	KCH_2_TMS	PMDETA	hexane	92
5	LiCH_2_TMS	PMDETA	hexane	0

a Standard conditions: 1.5 mmol of toluene, 1 mmol of base. Stirred for 2 h in 5 mL of solvent, then freeze–pump–thawed and replaced Ar atmosphere for CO_2_. Stirred for 10 min upon reaching r.t. Yields were determined by ^1^H NMR spectroscopy using hexamethylbenzene as the internal standard.

b 2 equiv of toluene.

It is important to note that KCH_2_TMS can conveniently be synthesized from commercially available LiCH_2_TMS and KO*t*Bu. However, when the same reaction was performed exclusively using LiCH_2_TMS and PMDETA, no toluene metalation was observed, highlighting the superior metalation power of potassium over the lithium reagent. As the formation of **5** could not be detected, this reaction furnished (trimethylsilyl)methylcarboxylic acid, stemming from the carboxylation of LiCH_2_TMS, which was identified spectroscopically with a yield of 94% (entry 5).

With a reliable protocol for the synthesis of benzylpotassium compounds in hand, we next targeted their selective coupling with aryl halides. We first chose the direct cross-coupling of parent benzylpotassium and 4-chloroanisole as the test reaction using toluene as the solvent and 3 equiv of TMEDA (*N*,*N*,*N*′,*N*′-tetramethyl-ethylene-1,2-diamine) as the solubilizing additive. Given the outstanding performance of the joYPhos ligand in the coupling of organolithium reagents, we chose this ligand in combination with the palladium(indenyl) precursor as a rapidly initiating precatalyst (**P2**).^45,46^ The reaction was carried out by performing the metalation in hexane, followed by a solvent switch to toluene and addition of the catalyst and aryl chloride. To our delight, the initial attempt carried out at room temperature gave the coupling product **4aa** already in a good yield of 70% (Table 2, entry 1). Changing from toluene to hexane as solvent led to a further improvement (Table 2, entry 2), while ethereal solvents such as THF or diethyl ether resulted in a significant drop of yield due to the formation of the homocoupling product and substrate decomposition (see Supporting Information for details). Despite these promising initial results, further experiments suffered from reproducibility issues, which we later traced back to the use of nitrogen-donors such as PMDETA or TMEDA as additives (vide infra). A possible explanation could be the high reactivity of the PMDETA/TMEDA solvated derivative, which can favor the formation of smaller aggregates, making the relevant benzylpotassium more prone to decomposition. Therefore, we decided to focus our further investigations on the establishment of a Lewis base-free protocol. Without TMEDA, the yield of the reaction dropped significantly to 26% (Table 2, entry 3). Changing the temperature or using salt additives also resulted in low yields for the formation of **4aa** (Table 2, entries 4, 5 and 8). However, the amount of potassium reagent was found to play a decisive role in the reaction outcome. When increasing the equivalents of the organometallic reagent from 1 to 3 equiv at a 0.05 M concentration, the yield significantly increased to 89% (Table 2, entry 6). To test whether this effect was concentration dependent, the reaction was carried out with 1 equiv of benzylpotassium at the same concentration of 0.05 M. However, the conversion was significantly reduced to 58%, confirming that an excess of benzylpotassium is advantageous for the reaction, presumably because of its limited solubility and possible deprotonation of the product as a side reaction (Table 2, entry 7).

**Table 2:**
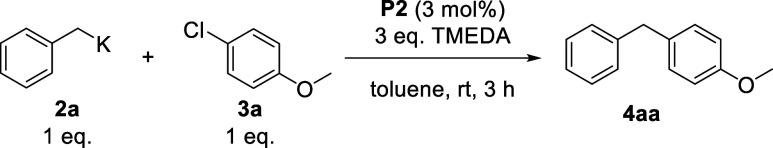
Screening Reactions for the Reaction of Benzylpotassium with 4-Chloroanisole^a^

entry	variation from standard conditions	yield (%)
1	none	70
2	3 equiv of TMEDA in hexane	82
3	no additives in hexane at r.t.	26
4	no additives in hexane at 65 °C	19
5	3 equiv of TMEDA, 10% CuOTf_2_ in hexane	30
6	3 equiv of BnK, *c* = 0.05 M, no TMEDA	89
7	1 equiv of BnK, *c* = 0.05 M, no TMEDA	58
8	*T* = 100 °C, no TMEDA	31
9	slow addition of B*n*K slurry	39
10	**P1** instead of **P2**	83
11	**P3** instead of **P2**	17
12	**P4** instead of **P2**	5
13	**P5** instead of **P2**	85
14	P(tBu)_3_ (3 mol %)	4
15	Pd-PEPPSI-IPent (3 mol %)	23
16	no catalyst	5
17	3 equiv of B*n*K, **P1** (3 mol %), no additives	95
18	**P1** (3 mol %), no additives, 3 equiv of **2a** in situ formed from LiCH_2_SiMe_3_ and KO*t*Bu	89

a Standard conditions: 0.25 mmol B*n*K and 3 mol % cat. were placed in a vial and suspended in the solvent. Addition of 0.25 mmol ArCl. Stirring for 3 h. Yield determined via GC-FID using 0.25 mmol of tetradecane as the internal standard.
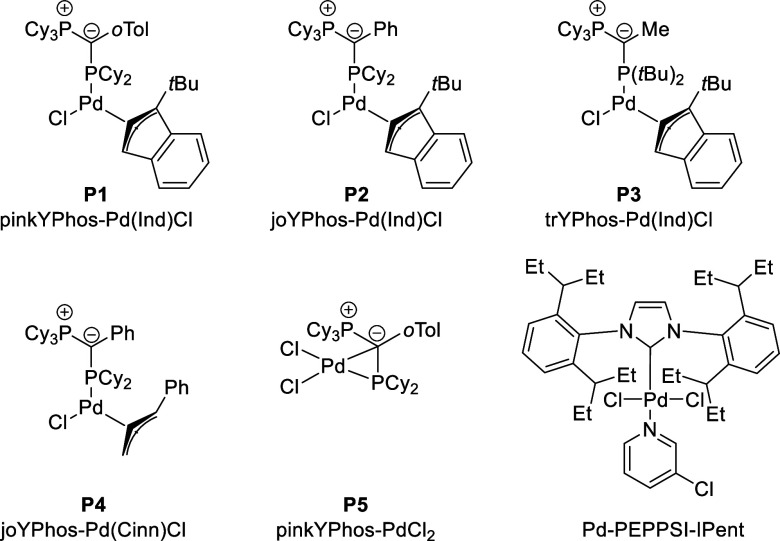

Due to the required excess of the organopotassium compound, small amounts of homocoupling product were observed, particularly for the reactions with small potassium organyls. It is interesting to note that contrary to the reported conditions of Pd catalyzed Murahashi couplings, it was not necessary to slowly add the organometallic reagent to the reaction mixture. In contrast, superior results were obtained with rapid addition, thereby facilitating the overall handling of the process (Table 2, entry 9). Overall, the reaction was found to be exceptionally fast. Reaction monitoring indicated completion within less than 10 min reaction time (Figure S14).

We next evaluated other palladium catalysts. Screening of a series of defined palladium precursors revealed a decisive impact of the phosphine ligand on the reaction success. Within our YPhos family, the ligand with an *o*-tolyl substituent in the backbone, **pinkYPhos**, achieved the highest conversion for our standard substrate **4aa** (Table 2, entries 10 and 17). Using a different Pd-source (cinnamyl instead of indenyl; entry 12 and 13) resulted in diminished yields. Thus, the Pd-indenyl system **P1** was used for all of the further screenings. Other catalyst systems, which were previously successfully employed in the coupling of organolithium and organosodium reagents,^22^ were unable to catalyze the reaction with comparable yields to our YPhos catalysts at the employed reaction conditions (Table 2, entries 14–15). It is likely that P(*t*Bu)_3_ is not able to undergo oxidative addition with aryl chlorides sufficiently fast under the mild reaction conditions (room temperature), whereas the NHC-based catalyst was able to deliver the product but not in high yields. Indeed, Takai et al. reported that temperatures of 70 °C are required for the direct coupling of aryl sodium reagents with aryl chlorides.^19^ Likewise, performing the reaction without a Pd catalyst resulted in only trace amounts of the product (Table 1, entry 16). Overall, the optimal reaction conditions turned out to be a 3 mol % catalyst loading of **P1** without any additives in toluene (Table 2, entry 17) furnishing **4aa** in a 95% yield. It is noteworthy that the coupling can also be conducted with in situ formed potassium reagents. For example, benzylpotassium prepared from a commercially available solution of LiCH_2_TMS and KO*t*Bu using a slight excess of toluene in hexane yielded 89% of the desired product (Entry 18, Table 2). Furthermore, it is also possible to utilize the Lochmann-Schlosser base for in situ potassiation (Figure 4).

**Figure 2:**
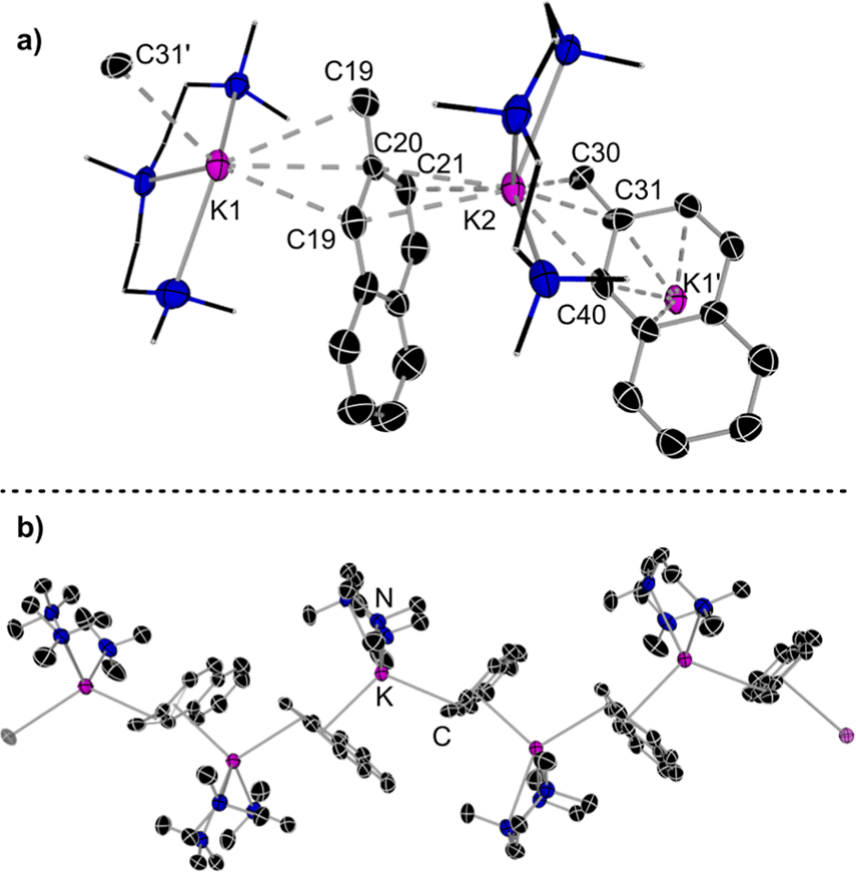
Solid structure of [**2c**·PMDETA]_∞_. Hydrogen atoms are omitted for clarity. Ellipsoids are shown at the 50% probability level. (a) Asymmetric unit. (b) 1D polymeric structure.

To further understand the obtained results, we next focused on the characterization of the organopotassium intermediates for a selection of substrates (toluene, 2-methylnaphthalene and 1-methylnapthalene). While in all cases, we observed the formation of the relevant benzylpotassium species, these intermediates are very insoluble in noncoordinating organic solvents, which precluded their crystallization. However, we found that addition of the tridentate Lewis donor PMDETA resulted in significantly more soluble intermediates, and in the case of 2-methylnaphthene, it was possible to isolate and structurally authenticate potassiated derivative [{(PMDETA)K(CH_2_C_10_H_7_)}_∞_] (**2c**). X-ray crystallographic studies established the polymeric constitution of **2c,** displaying a 1D zigzag chain structure, made up by π-interactions of the soft potassium cations with the 2-methylnaphthyl units (Figure 2b). Each potassium is chelated by a tridentate PMDETA ligand and coordinates to a 2-methylnaphthyl in a η^3^ fashion via its benzylic (Cα), *C*_ipso_, and one *C*_ortho_ atoms. Coordinative saturation of the soft *K* centers is achieved by π-engaging with the aromatic ring of a neighboring unit via η^3^-or η^4^ interactions, which generates two slightly different coordination environments for the *K* centers in the asymmetric unit of **2c** (K1 and K2 in Figure 2a). While this polymeric motif is reminiscent to others found in the literature for benzylpotasium derivatives,^47−50^ it is interesting to note that in **2c,** the *K*–*C*_ipso_ contacts [mean value, 3.1415) Å] are noticeably shorter than the *K*–*C*_α_ bonds [mean value, 3.532) Å]. These bonding preferences coupled with the relatively short *C*_α_–*C*_ipso_ [mean value, 1.380 Å] are consistent with the delocalization of the negative charge into the aromatic ring and with the CH_2_ group gaining more sp^2^ character. Supporting this interpretation, ^1^H and ^13^C NMR spectroscopic studies of **2c** in C_6_D_6_ solutions showed an informative downfield signal at 3.10 ppm in the ^1^H NMR spectra for the CH_2_ group, whereas the aromatic resonances are significantly shielded compared to usual aromatic shifts (ranging from 7.01 to 5.79 ppm, see Supporting Information for details). This trend in chemical shifts has been previously noted by Mulvey when comparing the spectroscopic data of monomeric alkali-metal benzyl derivatives (M = Li, Na and K) and has been interpreted as an indication of significant charge delocalization in the benzyl group.^51^

In C_6_D_6_ solutions, **2c** seems to retain some of its highly aggregated structure as indicated by ^1^H DOSY NMR spectroscopic studies which estimated a molecular weight above 1052 g mol^–1^ (see Supporting Information for details), which is significantly larger than those calculated for the formation of monomeric or dimeric arrangements (353.22 and 706.44 g mol^–1^, respectively).^52^ These findings contrasts with previous DOSY studies of related benzyl sodium derivatives in C_6_D_6_ solutions, which tend to adopt monomeric motifs.^33,53^

**Figure 3:**
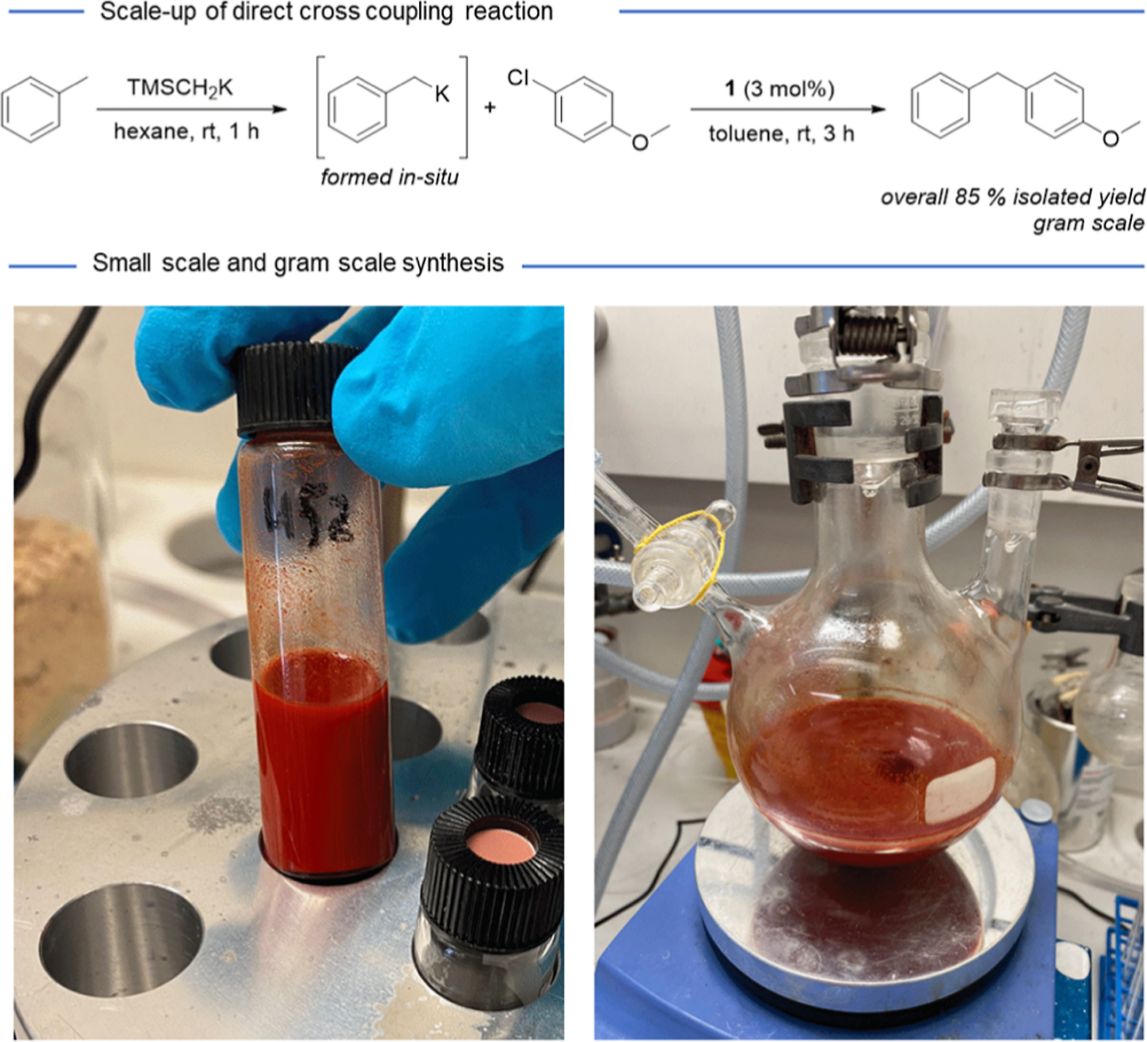
Standard scale of the reaction (left, 0.25 mmol) and scale-up of the cross-coupling (right, 7 mmol).

Importantly, NMR studies of PMDETA adduct **2c** with 4-chloroanisole showed fast decomposition without observing formation of any cross-coupled product. These findings suggest that formation of more soluble and less aggregated organopotassium species is detrimental for the success of the Pd-catalyzed C–C bond forming step, favoring instead alternative side reaction pathways. Thus, it appears that the low solubility of the potassium intermediates, which typically is considered an operational challenge in the use of these reagents in synthesis, here has a positive effect. Notably, under optimal catalytic conditions, the reaction mixture remains a suspension throughout the entire process (Figure 3). This suggests that a low concentration of the benzylpotassium reagent in solution is critical to minimize unwanted side reactions with the aryl chloride.

Furthermore, we observed side reactions in cross-coupling reactions with TMEDA. As previously reported by Newman for the coupling of organolithium compounds,^39^ TMEDA is readily cleaved under the reaction conditions to form potassium dimethylamide, which subsequently undergoes a Buchwald–Hartwig type coupling as side-reaction. Moreover, both TMEDA and PMDETA have been reported to undergo deprotonation with strong metal bases, leading to further metalated species that could potentially participate in coupling processes, thereby resulting in the formation of side products.^54−57^

**Figure 4:**
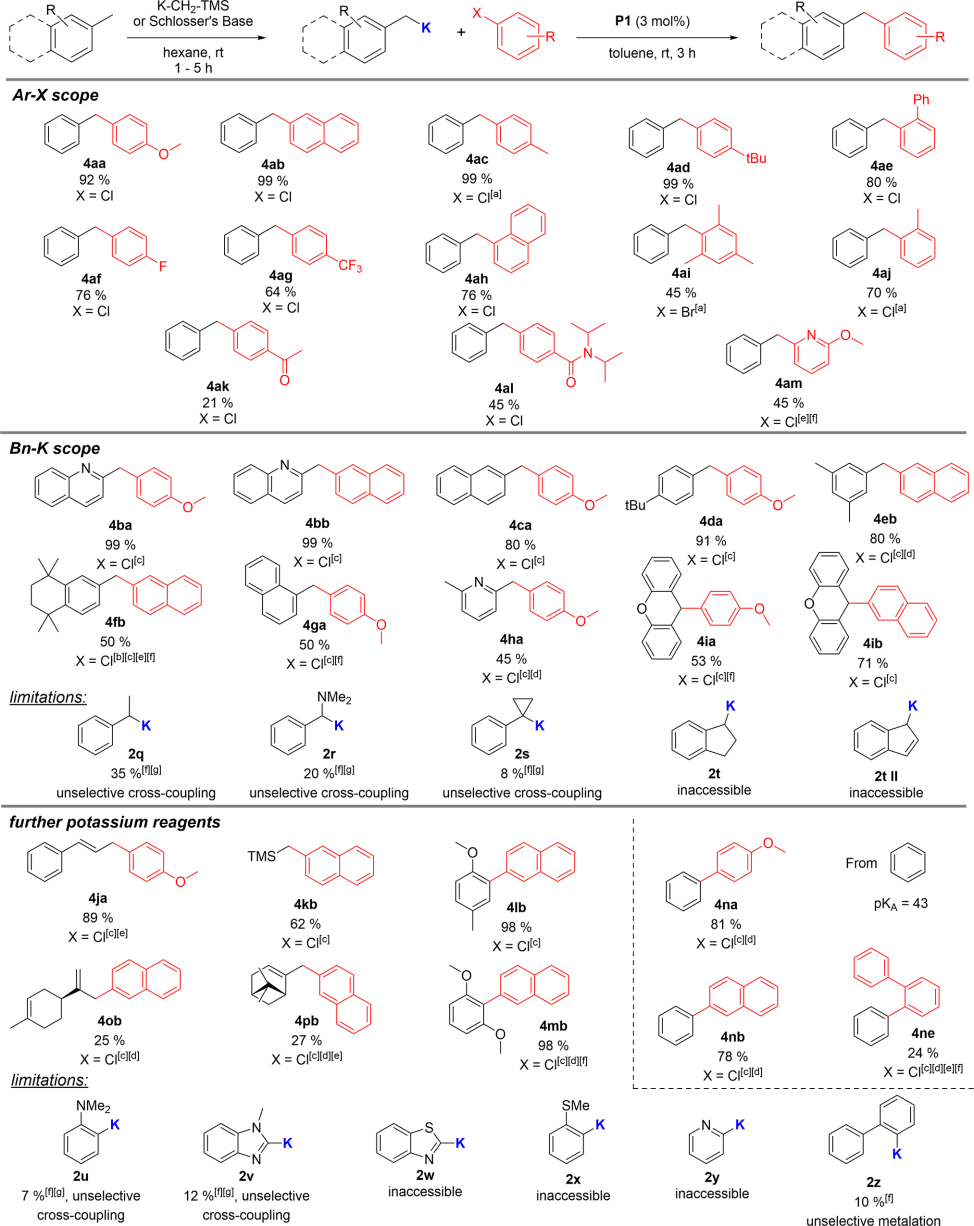
1 equiv (0.25 mmol) aryl halide, 3 equiv (0.75 mmol) potassium reagent, 0.03 equiv (0.0075 mmol) **P1**, 5 mL toluene (*c* = 0.05 M), fast addition of solvent, and 3 h at room temperature. [a] Isolated as a mixture with 5% homocoupling product of potassium organyl, clean NMR obtained after PTLC. [b] 5 mol % catalyst, [c] benzene as solvent, [d] deprotonation with Schlosser’s base. [e] Mixture of isomers after cross-coupling. [f] GC-Conversion. [g] Yield refers to the reaction with 4-chloroanisol.

Next, we evaluated the scope of the established protocol. To our delight, a variety of aryl halides were successfully coupled with benzylpotassium under the optimized reaction conditions (Figure 4). Excellent yields were achieved with 4-chloroanisole (**4aa**), 2-chloronaphthalene (**4ab**) and para-alkyl substituted aryl chlorides (**4ac**, **4ad**). Mono-ortho substituted aryl-halides (**4ae**, **4ah**, **4aj**) gave likewise good to high yields, whereas more sterically demanding di-ortho-substituted substrates such as mesityl bromide led to significantly lower yields, hinting toward a steric limit for our catalyst system (**4ai**). Substrates with electron-withdrawing (F, CF_3_) groups were also successfully coupled in moderate to good yields (**4af**, **4ag**). Even amide functionalities and ketones containing unsaturated C=O groups were tolerated but resulted in lower yields (**4ak**, **4al**). Given the notoriously high sensitivity of these functional groups toward benzylpotassium compounds, the successful isolation of these products is still remarkable and indicates an extremely fast coupling process. Coupling with heteroaryl chlorides also delivered serviceable yields (**4am**).

Besides different aryl halides, we tested the applicability of the protocol toward different benzylpotassium compounds without their intermediate purification. Potassiated quinaldine could be coupled with *p*-chloroanisole (**4ba**) and 2-chloronaphthalene (**4bb**) in quantitative yields. Other heteroaryl-derived benzylpotassium derivatives could also be successfully cross-coupled (**4ha**). Reactions with *p*-*tert*-butyl benzylpotassium, mesityl potassium, and potassiated 2-methyl naphthalene also resulted in good yields of **4ca**, **4eb,** and **4da**, respectively, whereas the isomeric 1-methyl naphthalene only gave moderate yields (**4ga**). Moderate yields were also recorded for other sterically demanding benzylpotassium reagents, such as xanthene (**4ia**, **4ib**) and 1,1,4,4,6-pentamethyl-2,2,3,3-tetrahydro-naphthalene (**4fb**). This observation highlights the steric limitations of our protocol. Accordingly, secondary and tertiary benzylpotassium reagents led to a clear decline in yields (**2q**, **2s**). Benzylpotassium reagents featuring amine substituents also gave poor yields (**2r**). Moreover, both types of substrates were found to be difficult to isolate due to the formation of inseparable side products. Our protocol failed for the metalation of Indane (**21**), as under our cross-coupling conditions, indene was observed as the sole isolable product of the reaction. Indene itself could not be successfully be metalated.

Besides benzylpotassium reagents, we also investigated other potassium reagents, such as (trimethylsilyl)methylpotassium (**4kb**) or cinnamyl potassium (**4ja**). The latter reacted readily with aryl halides and selectively led to the cross coupling at the terminal position with a 5:1 ratio of E/Z isomers. Furthermore, compounds that readily undergo directed *ortho* metalation (DoM) such as 4-methylanisole and 1,3-dimethoxybenzene were also coupled successfully in a C(sp^2^)-C(sp^2^) cross coupling reaction (**4 lb**, **4mb**). The true benefit of using strong potassium bases becomes clear from the direct C–H metalation and subsequent cross-coupling of nonactivated benzene. Using the Lochmann-Schlosser base, metalation of benzene (p*K*_a_ = 43) is easily accomplished at room temperature^28^ and directly used in the high-yielding coupling with 4-chloroanisole (**4na**) and 2-chloronaphthalene (**4nb**), respectively. Lower yields were obtained for more sterically demanding aryl chlorides (**4ne**). This example demonstrates the untapped potential of the heavier alkali metal bases in C–H metalation/coupling protocols, obviating the need for functionalized (halogenated) aromatics. Furthermore, the protocol was utilized for the direct metalation of terpenes such as (−)-limonene and β-pinene, which both were successfully cross coupled (**4ob**, **4pb**). In the case of β-pinene, the overall yield of the cross coupling was 50%, but it resulted in a mixture of products, from which only **4pb** could be isolated cleanly.

The robustness of the protocol was showcased by performing the reaction at 28-fold scale, resulting in no significant loss in yield (Figure 3). Thus, compound **4aa** could be isolated on a 1.2 g scale in 85% yield. Due to the simple one-pot reaction of the developed protocol, scale-up was easily accomplished compared to the Murahashi-coupling, which requires a slow addition of the alkali metal reagent via a syringe pump. It is important to note, however, that the purity of the arylpotassium compound is critical. Allowing the mixture to remain at room temperature for an extended period during the metalation step increased the formation of unwanted side products, ultimately leading to lower yields in the subsequent cross-coupling reaction. Overall, the examples shown in Figure 4 demonstrate the applicability of our protocol to a range of aryl chlorides and potassium reagents.

Limitations particularly stem from steric congestion, which hampers the transformation of bulky aryl chlorides and tertiary benzylpotassium compounds and the unavailability of the potassium reagent through direct C–H metalation (**2w**, **2x**, **2y** and **2z**). Also, functional group tolerance and the cross-coupling of more complex heteroarenes (**2v**, **2w**) are problematic, but they can potentially be mitigated through the development of faster catalysts in the future (c.f. **4al**). The development of the highly electron-rich YPhos ligands is crucial for directing the high reactivity of potassium organyls toward cross-coupling. With the ongoing development of more potent and more selective s-block metal bases for the direct metalation of unreactive compounds,^58,59^ as well as the development of improved catalyst systems, this protocol will certainly be extended and gain greater significance in coupling chemistry in the future.

## Conclusions

In summary, we have reported the first direct cross coupling of organopotassium compounds with aryl halides. While the direct metalation using a Lochmann Schlosser base has proven successful, (trimethylsilyl)methylpotassium emerged as the most effective reagent to reliably access the potassiated compounds by direct C–H metalation. The potassium reagents can be utilized either as isolated solids or solutions prepared in situ from commercially available (trimethylsilyl)methyllithium and potassium *tert*-butoxide. The subsequent coupling reaction is enabled by a palladium catalyst equipped with a highly electron-rich, ylide-substituted phosphine, which allows for fast oxidative addition and transmetalation, thus minimizing the usually dominant side and decomposition reactions observed with these heavy alkali metal bases. The limited solubility of the generated organopotassium species seems to be an advantage in this protocol in order to minimize side reactions or catalyst degradation. The protocol is applicable to a variety of aryl halides and benzylpotassium reagents and therefore enables sp^3^-sp^2^ and sp^2^-sp^2^ couplings including the coupling of simple phenyl potassium formed by direct C–H metalation of parent benzene. The presented combination of direct C–H metalation with potassium bases and direct cross-coupling furthermore showcases that the chemistry of heavier s-block organyls can be further advanced by continuous development of more active catalysts. Although this strategy is still limited by the accessibility of organopotassium reagents by direct metalation, the development of new, more powerful metalation reagents in recent years promises broader applications of this strategy in the future.

## Supporting Information Available

General procedures, NMR spectra of all isolated compounds, and details of the catalysis optimization and crystal structure analyses (PDF)
