# Supplementary material for: Harnessing Organopotassium Reagents for Cross-Coupling with YPhos-Pd Catalysts: Opportunities, Applications, and Challenges
Source: J Am Chem Soc. 2025 Feb 2;147(6):5417–25. doi: 10.1021/jacs.4c18073 (PMC11826883; doi:10.1021/jacs.4c18073)
Supplement: Supplementary file 1 — ja4c18073_si_001.pdf [file ja4c18073_si_001.pdf]

Harnessing Organopotassium Reagents for Cross-Coupling with YPhos-Pd Catalysts: Opportunities, Applications, and Challenges

Daniel Knyszek[1], Julian Löffler[1], David E. Anderson[2], Eva Hevia[2]*, Viktoria H. Gessner[1]*

[1] Inorganic Chemistry II, Faculty of Chemistry and Biochemistry, Ruhr-University Bochum, Universitätsstraße 150, 44801 Bochum, Germany

[2] Department für Chemie und Biochemie, Universität Bern, Freiestrasse 3, 3012 Bern, Switzerland

E-mail: viktoria.gessner@rub.de; eva.hevia@unibe.ch

Table of Contents

1. Experimental Details 2

1.1. General Experimental Information 2

1.2. Synthesis and isolation of potassium reagents 3

1.2.1. Synthesis of KCH_2SiMe_3 ($TMSCH_2K$) 3

1.2.2. Synthesis of [2c·PMDETA]∞ 3

1.3. Direct metalation of unactivated organic compounds 6

1.3.1. Direct metalation with Schlosser base in hexane 6

1.3.2. Direct metalation with Schlosser base in THF 6

1.3.3. Direct donor-free Metalation with $TMSCH_2K$ in hexane 8

1.3.4. Direct metalation with $TMSCH_2K$ in hexane with PMDETA 9

1.3.5. NMR experiments of potassium organyls 11

1.3.6. 1H-DOSY NMR study of 2c·PMDETA 13

1.4. Direct cross coupling of organopotassium compounds with aryl halides 14

1.4.1. General procedure for the direct cross coupling with isolated benzyl potassium 14

1.4.2. Screening of reaction conditions 16

2. Synthesis and characterization of the catalysis products 21

2.1. Procedures and spectroscopic details 21

2.2. NMR spectra of the isolated compounds 32

3. Xray crystallographic data 57

4. Contributions 63

5. References 63

1. Experimental Details

1.1. General Experimental Information

All experiments were carried out under a dry, oxygen-free argon atmosphere using standard Schlenk techniques. Involved solvents were dried using an MBraun SPS-800 (THF, DCM, toluene, acetonitrile, diethylether and pentane) or dried in accordance with standard procedures. Deuterated solvents were stored over molecular sieves in an argon-filled glovebox. All other reagents were purchased from Sigma-Aldrich (Merck), ABCR, TCI or Acros Organics or in case of palladium precursors donated by Umicore AG and Co KG. All reagents purchased from chemical suppliers were used without further purification. The YPhos ligands keYPhos[1], joYPhos,[2] pinkYPhos[3] were prepared according to literature procedures. References to the prepared organic compounds are given below for each compound.

NMR spectra were recorded on Avance-400 spectrometers at 25 °C if not stated otherwise. All values of the chemical shift are in ppm regarding the δ-scale. All spin-spin coupling constants (*J*) are printed in Hertz (Hz). To display multiplicities and signal forms correctly the following abbreviations were used: s = singlet, d = doublet, t = triplet m = multiplet, dd = doublet of doublet, br = broad signal. Signal assignment was supported by, HSQC (^{1}H / ^{13}C), HMBC (^{1}H / ^{13}C) correlation experiments.

GC/MS analyses were carried out with an Agilent 8890 GC and 5977B MSD system using an HP-5 capillary column (Phenyl methyl siloxane, 30 m × 320 × 0.25, 100/2.3-30-300/3, 2 min at 60 °C, heating rate 30 °C/min, 3 or 10 min at 300 °C). Yields were determined by GC-FID using *n*-tetradecane as internal standard.

Elemental analyses were performed on an Elementar vario MICRO-cube elemental analyzer in the in-house analytical facility.

Column chromatography was performed in standard glass columns of reasonable length with Silica Gel (0.06 – 0.2 mm) for the stationary phase. All solvents were distilled prior to performing column chromatography.

Caution: Benzyl potassium and other organopotassium and alkali reagents, especially as neat compounds, are severely air-/moisture-sensitive and pyrophoric organometallic compounds. These compounds need to be handled under an inert gas atmosphere to exclude reactions with oxygen and water. Guidelines for their handling can be found in literature: T. L. Rathman, J. A. Schwindeman, *Org. Process Res. Dev.* **2014**, *18*, 1192.

1.2. Synthesis and isolation of potassium reagents

1.2.1. Synthesis of KCH_2SiMe_3 ($TMSCH_2K$)

In an argon-filled Schlenk flask at 0 °C, 50.0 mL dry hexane was added to 2.24 g (20 mmol) KOtBu and 1.88 g (20 mmol) $LiCH_2SiMe_3$ affording a fine, colorless suspension which was stirred for 2 h. The suspension was warmed to ambient temperature and stirred overnight. Isolation of the colorless precipitate (KCH_2SiMe_3) was achieved by gravity filtration using a glass-tapped filter frit. The filter cake was washed with 3 x 15 mL aliquots of hexane until the liquors ran completely clear. The solid was dried under vacuum and stored in the glovebox for further use. Typical yield = 86 %, 2.18 g.

1.2.2. Synthesis of [2c·PMDETA]$_\infty$

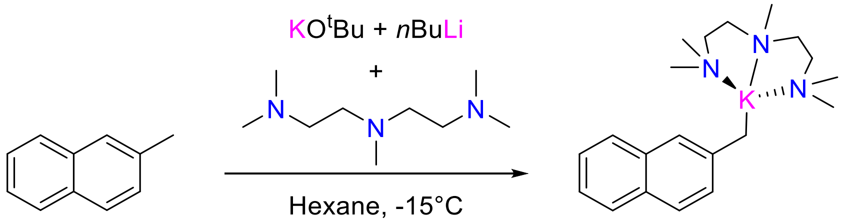

In an argon-flushed Schlenk flask, KO^{t}Bu (2.0 mmol, 224 mg) was suspended in hexanes (7 mL), to which 2-methylnaphthalene (2.2 mmol, 300 mg) and *n*BuLi (2.5 M) (2.0 mmol, 0.800 mL) were added. The resultant dark purple suspension was stirred at room temperature for 2 h. PMDETA (5.0 mmol, 1.05 mL) was added to the suspension, followed by slow addition of THF (1.5 mL). The mixture was gently heated until the precipitate fully dissolved. The solution was slowly cooled to -15 °C, affording dark purple crystals of [**2c**·PMDETA]$_\infty$. The crystals were isolated by filtration, washed with cold pentane, and dried in vacuo affording dark purple X-ray quality crystals (502 mg, 0.71 mmol, 71%).

^{1}H NMR (400 MHz, C_6D_6) δ 7.01 (d, *J* = 7.6 Hz, 1H, C_{Ar}-H), 6.97 – 6.91 (m, 2H, C_{Ar}-H), 6.79 (d, *J* = 9.0 Hz, 1H, C_{Ar}-H), 6.62 (dd, *J* = 9.0, 2.2 Hz, 1H, C_{Ar}-H), 6.29 (ddd, *J* = 7.8, 5.1, 2.8 Hz, 1H, C_{Ar}-H), 5.79 (d, *J* = 2.1 Hz, 1H, C_{Ar}-H), 3.08 (q, *J* = 2.8 Hz, 2H, C_{Bn}-H), 2.00 (s, 12H, CH_3 PMDETA), 1.95 (s, 8H, CH_2 PMDETA), 1.94 (s, 3H, CH_3 PMDETA).

^{13}C NMR (75 MHz, C_6D_6) δ 149.9 (C_{Ar}), 141.9 (C_{Ar}), 128.6 (C_{Ar}), 127.2 (C_{Ar}), 126.1 (C_{Ar}), 122.8 (C_{Ar}), 119.8(C_{Ar}), 110.9 (C_{Ar}), 92.3 (C_{Ar}), 58.9 ($C_{Benzylic}$), 57.2 (CH_2 PMDETA), 55.9 (CH_2 PMDETA), 44.9 (CH_3 PMDETA) , 40.8 (CH_3 PMDETA).

Elemental analysis: Calculated for C20 H32 N3 K: C, 67.94; H, 9.12; N, 11.88, found: C, 67.20; H, 9.11; N, 12.21.

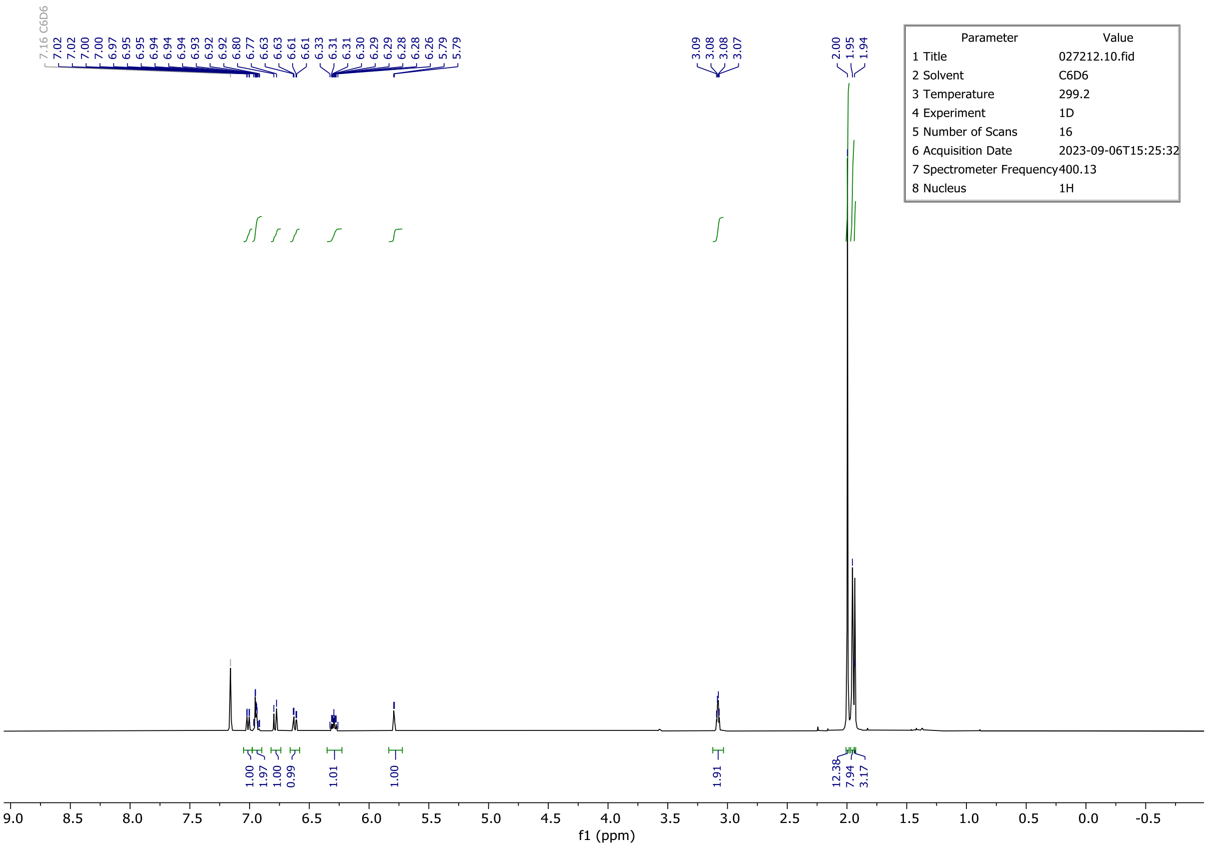

Figure S1. 1H NMR spectrum of [**2c**·PMDETA]$_{\infty}$ in C6D6

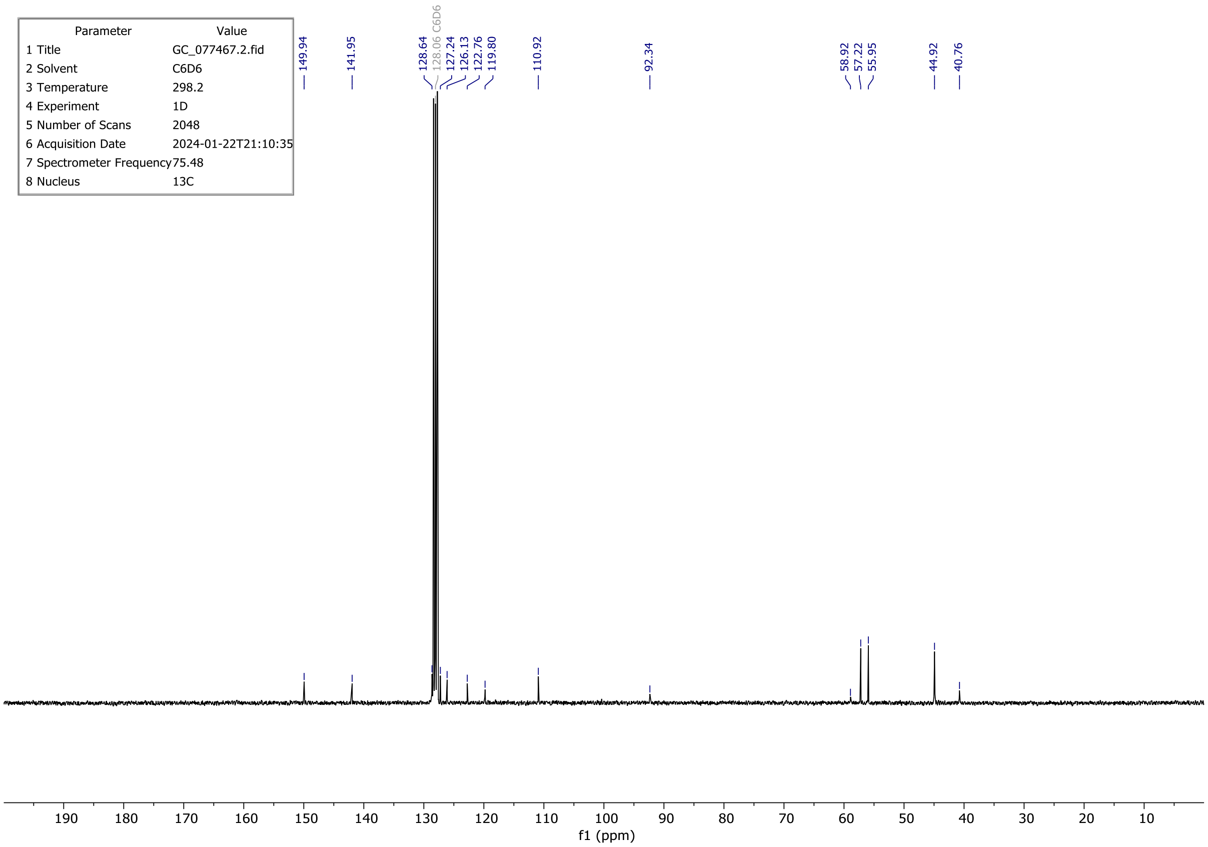

Figure S2. 13C NMR spectrum of [**2c**·PMDETA]$_\infty$ in C6D6.

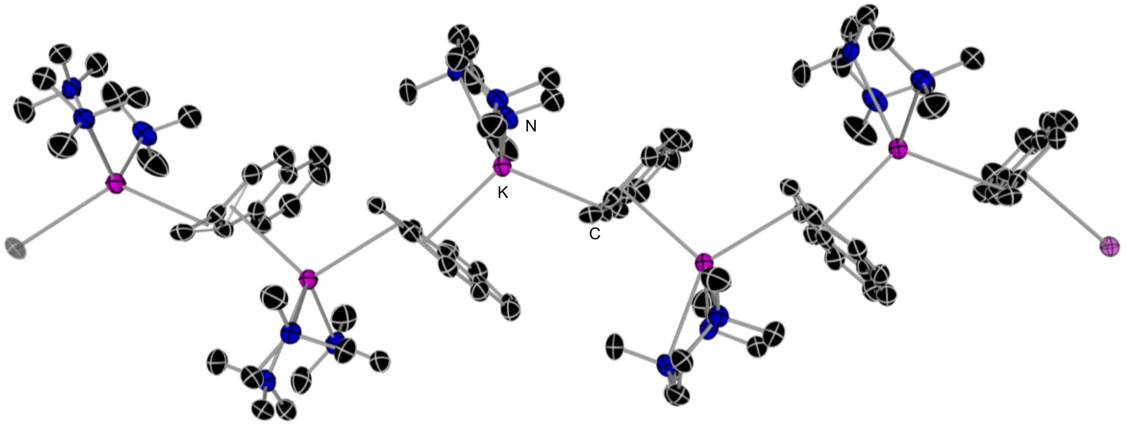

Figure S3. Polymeric structure of [**2c**·PMDETA]$_\infty$

1.3. Direct metalation of unactivated organic compounds

1.3.1. Direct metalation with Schlosser base in hexane

In an oven dried Schlenk flask, the toluene derivative (1 eq.) and KO*t*Bu (1 eq.) were suspended in dry hexane. After cooling to -79 °C, *n*BuLi (1 eq.) was added dropwise and the suspension was allowed to warm up to room temperature. Stirring was continued for 2 h and hexane was filtered off. The resulting solid was washed with hexane and dried under reduced pressure. The solid was used without further purifications.

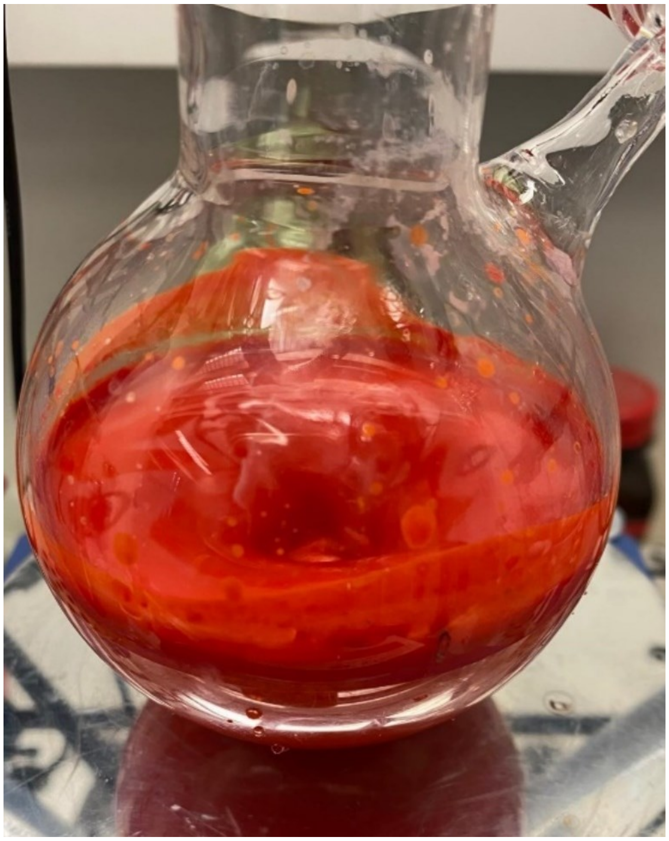

Figure S4. Benzyl potassium prepared via Lochmann-Schlosser superbase.

1.3.2. Direct metalation with Schlosser base in THF

In an oven dried Schlenk flask, the toluene derivative (1 eq.) and KO*t*Bu (1 eq.) were dissolved in dry THF. After cooling to -79 °C, *n*BuLi (1 eq.) was added dropwise and stirring was continued for 1 h at that temperature. The solution was allowed to warm up to – 20 °C, and the solvent was removed under reduced pressure at that temperature. The solid was washed with small amounts of hexane and dried under reduced pressure. The solid was used without further purifications.

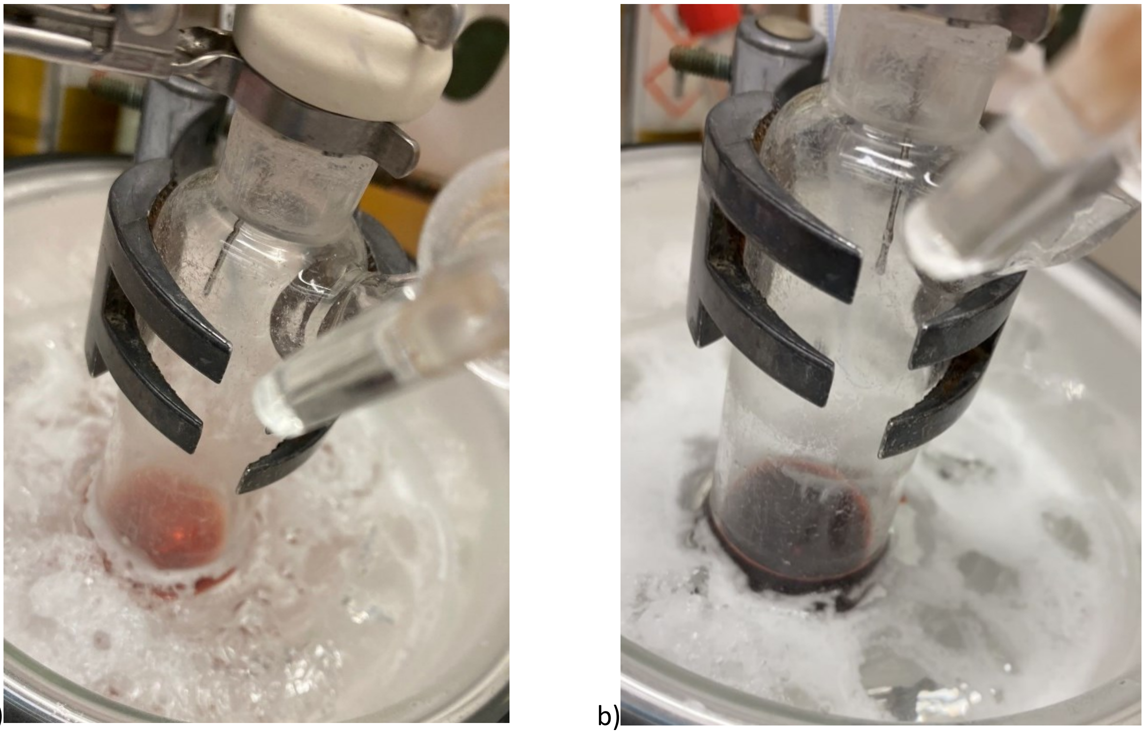

a) b)

Figure S5. Benzyl potassium prepared via Lochmann-Schlosser superbase in THF a) after addition of first few drops of *n*BuLi b) after completed addition of *n*BuLi.

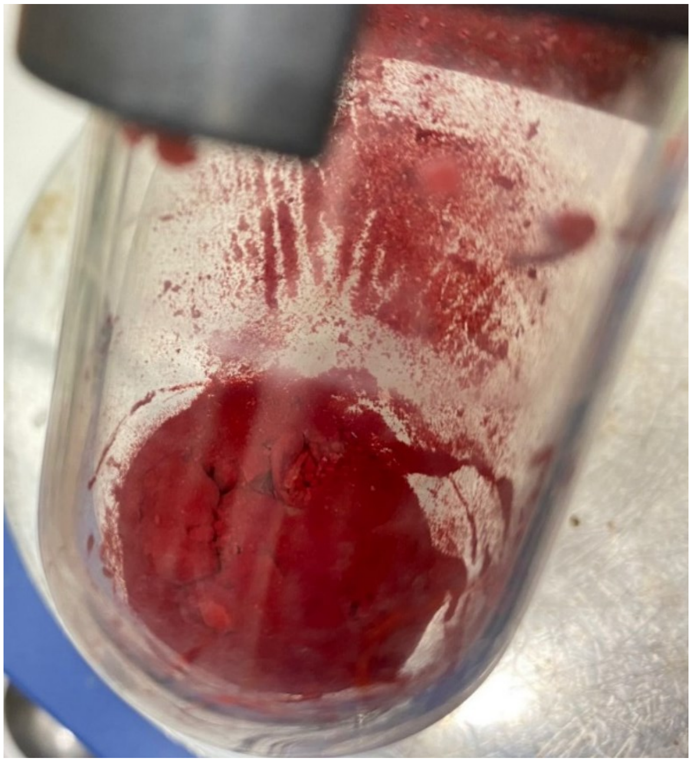

Figure S6. Isolated benzyl potassium with THF as donor.

1.3.3. Direct donor-free Metalation with $TMSCH_2K$ in hexane

In an oven dried Schlenk flask, $TMSCH_2K$ (1 eq.) was suspended in dry hexane and toluene derivative (1.5 eq.) was added to the suspension at 0 °C (if it is a solid, it was added with a funnel against an argon-flow). After allowing the suspension to warm up to room temperature, stirring was continued for 3 h. Solvent was removed under reduced pressure and the remaining solid was used without further purification.

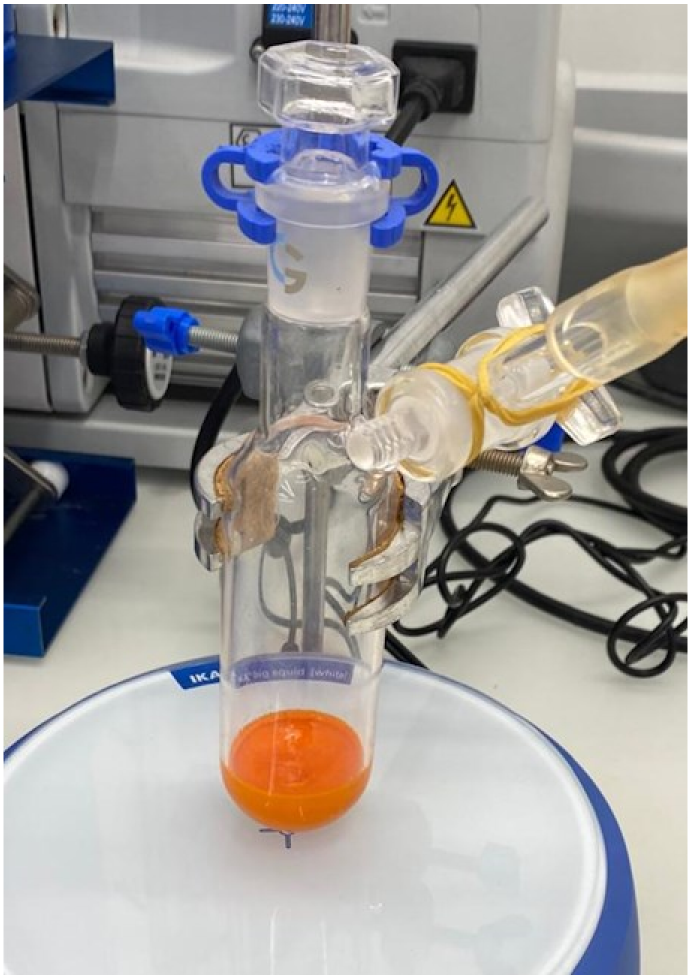

Figure S7. Benzyl potassium prepared via $TMSCH_2K$ in hexane without additional donor.

1.3.4. Direct metalation with $TMSCH_2K$ in hexane with PMDETA

In an oven dried Schlenk flask, $TMSCH_2K$ (1 eq.) was suspended in dry hexane. The mixture was cooled to 0 C and PMDETA was added. Immediately after, toluene derivative (1.5 eq.) was added to the suspension, and the mixture was allowed to warm up to room temperature. Stirring was continued for 1 h. Solvent was removed under reduced pressure to obtain the solvated benzyl potassium derivative.

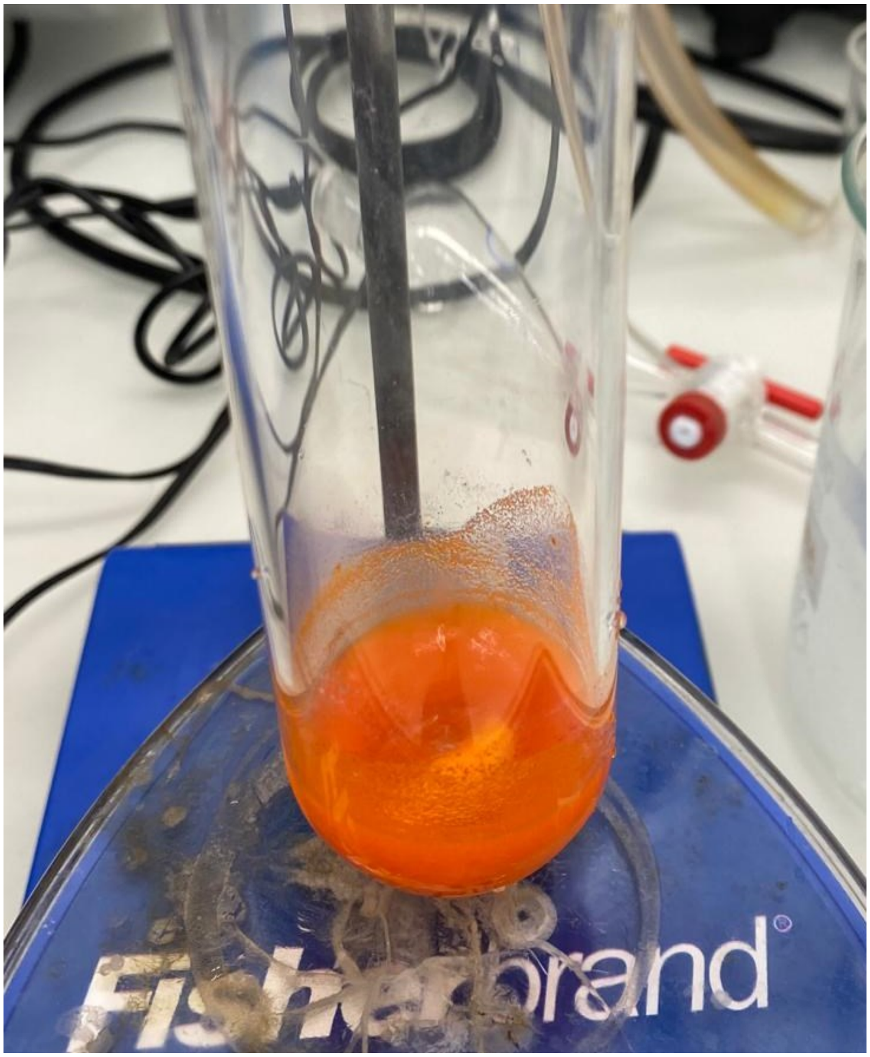

Figure S8. Benzyl potassium prepared via $TMSCH_2K$ in hexane with PMDETA as additional donor.

The yield of the metalation was determined by CO_2 quench and subsequent aqueous workup. The corresponding carboxylic acid was analyzed via NMR spectroscopy with hexamethyl-benzene as internal standard.

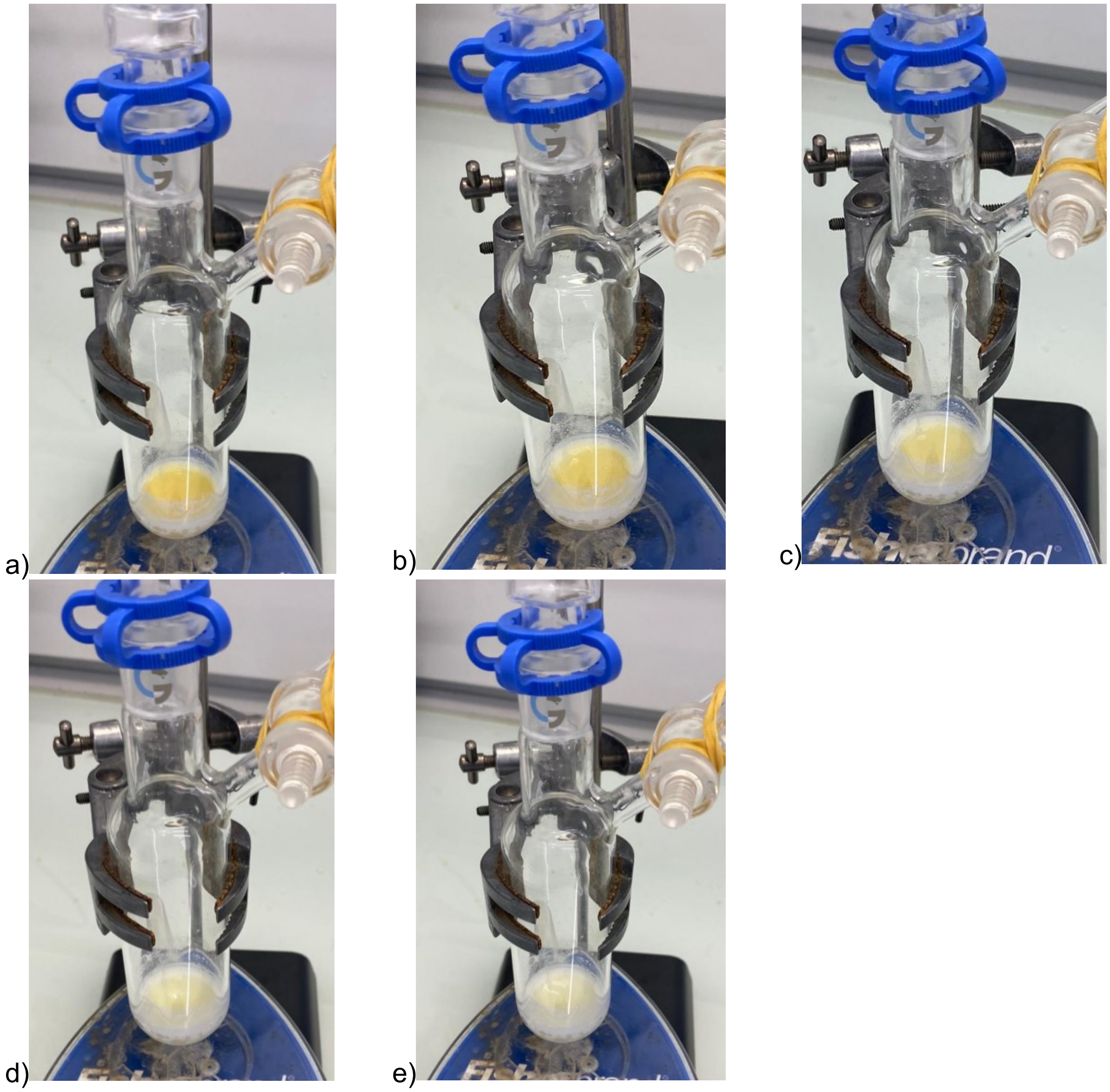

Figure S9. CO2 quench of benzyl potassium obtained via metalation in hexane with TMS-CH_2-K after CO_2 atmosphere was added (a). Gradual color change observed within one minute (b-d) and final product obtained after 2 min (e).

1.3.5. NMR experiments of potassium organyls

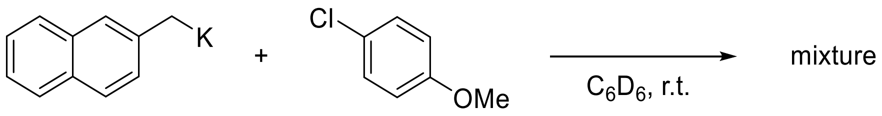

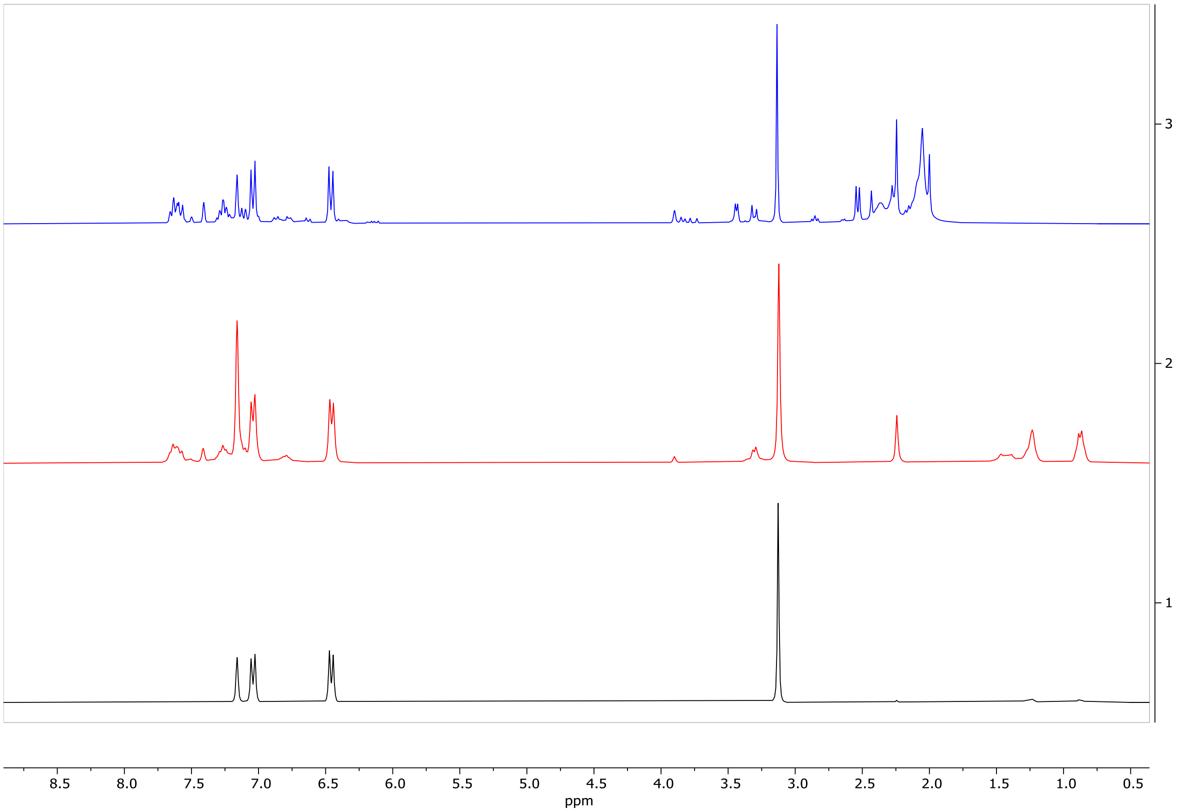

Figure S10. ^{1}H-NMR spectra of reaction of **2c** with 4-chloroanisole in C_6D_6 a) without donor after 30 min b) without donor after 4 days c) with PMDETA after 2 min.

The reaction of **2c** with 4-chloroanisole without donor showcases that the two reaction components are reasonably stable in the timeframe of the catalysis reaction, with decomposition only occurring after multiple days. The reaction of **2c**PMDETA with 4-chloroanisole leads to immediate decomposition.

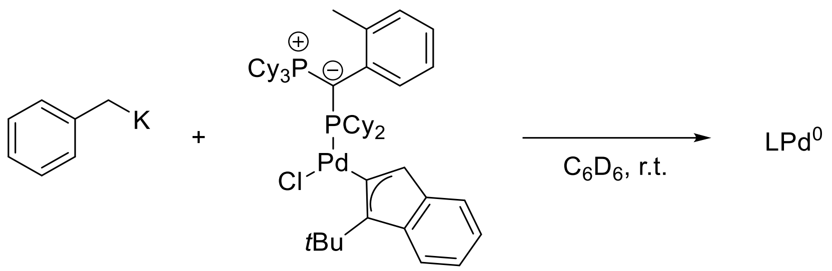

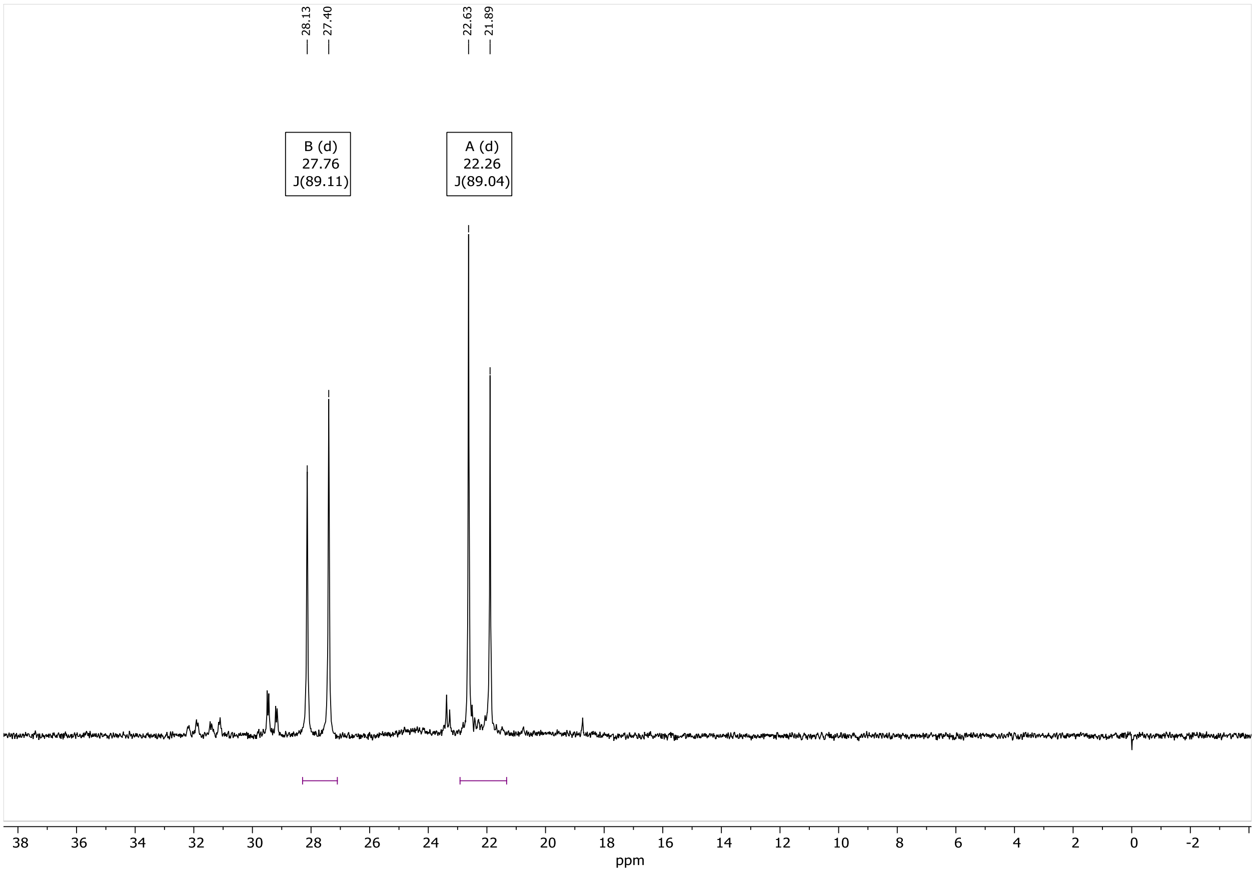

Figure S11. $^{31}P\{^1H\}$-NMR spectrum of reaction of **1c** with **P1** in C_6D_6.

From the $^{31}P\{^1H\}$-NMR data it can be assumed that a catalytically active Pd^0 species is formed after reduction of the Pd^{II} precursor by the potassium organyl.

1.3.6. 1H-DOSY NMR study of 2c·PMDETA

Diffusion Ordered Spectroscopy (DOSY) experiment was conducted by NMR using the External Calibration Curve (ECC) method at 15 mM in C_6D_6 as described by Stalke[4]. Data was accumulated by linearly varying the diffusion encoding gradients over a range of 2% to 95% for 32 gradient values. The signal decay dimension on the pseudo-2D data was generated by Fourier transformation of the time-domain data. The diffusion profile and coefficients were ascertained by use of the DOSY processing features of TopSpin software. The peak of residual proteo- solvent was used as an internal standard.

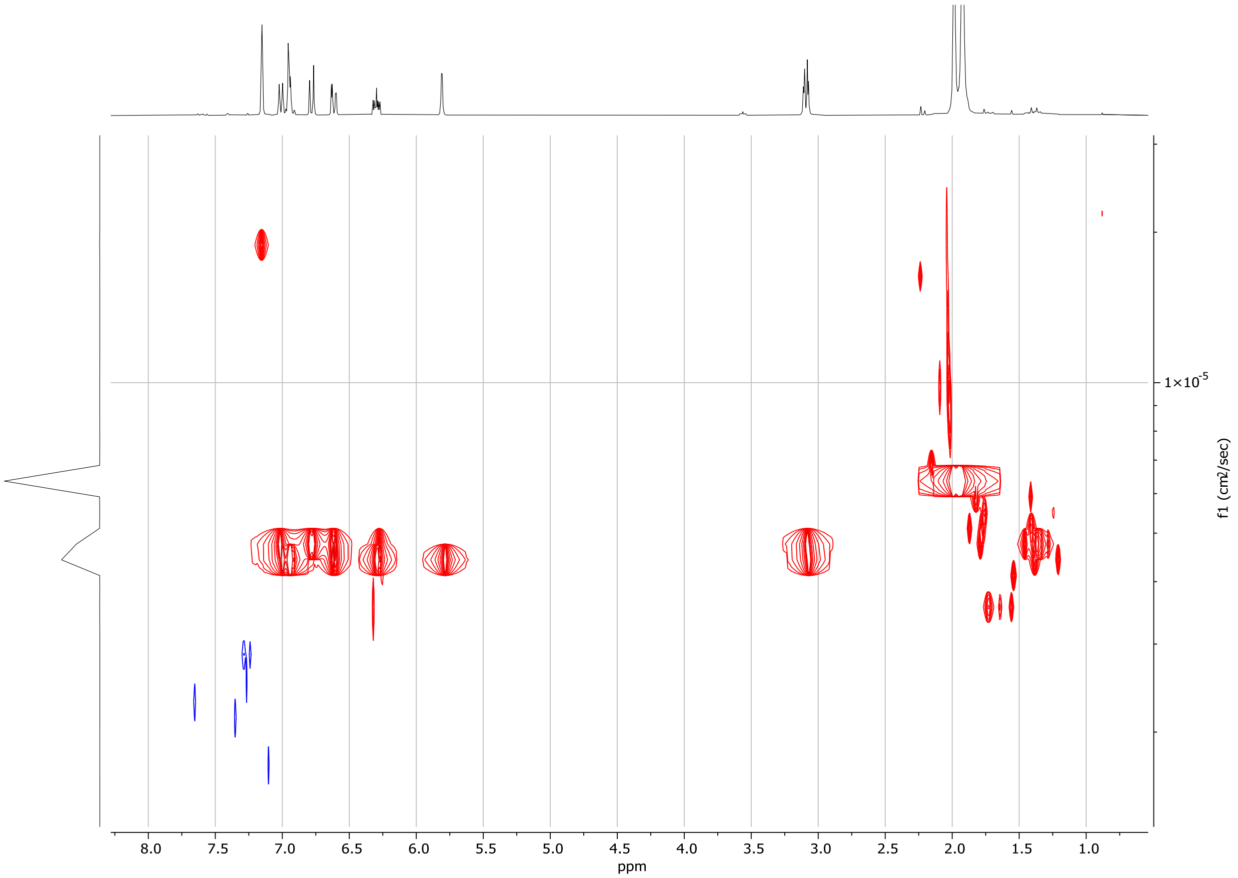

Figure S12. 1H-DOSY NMR spectrum of **2c**PMDETA.

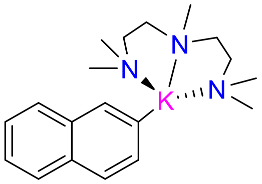

Monomer = 353.22 g mol^{-1}
Dimer = 706.44 g mol^{-1}
Trimer = 1059.66 g mol^{-1}

Using C_6D_6 as an internal standard, we calculated the estimated molecular weight of the [(PMDETA)(**2c**)] aggregate to be:

CS – 1730 g mol^{-1}

MERGE – 1242 g mol^{-1}

DSE – 1052 g mol^{-1}

Diffusion coefficient D_x = 4.5 10^{-10} m^2 s^{-1}

1.4. Direct cross coupling of organopotassium compounds with aryl halides

1.4.1. General procedure for the direct cross coupling with isolated benzyl potassium

A: In a glovebox, a vial was charged with the organopotassium (0.75 mmol, 3 eq.) made via direct metalation using Schlosser's base, and pinkYPhos-Pd(Ind)Cl **1** (0.0075 mmol, 3 mol%). The vial was sealed with a septum cap and taken outside the glovebox. To this vial a stock solution of aryl halide (0.25 mmol, 1 eq., 0.05 M in toluene/benzene) was added at once and the mixture was stirred rapidly for 3 h at room temperature. Subsequently, the reaction mixture was quenched with water and extracted with EtOAc three times. The organic phases were combined and dried with $MgSO_4$. The crude product was purified via column chromatography.

General procedure for the direct cross coupling in one-pot

B: In a glovebox, a Schlenk flask was charged with TMS-CH_2-K (0.75 mmol, 3 eq). The flask was taken out of the box. The base was suspended in 6 ml hexane and the toluene derivative was added (1.125 mmol, 4.5 eq.) and the mixture was stirred for 3 - 12 h. The solvent was removed under reduced pressure. To the resulting solid, pinkYPhos-Pd(Ind)Cl **1** (0.0075 mmol, 3 mol%) was added and the solids were suspended in toluene or benzene. The aryl chloride (0.25 mmol, 1 eq.) was added quickly and the mixture was stirred for 3 h. The reaction mixture was quenched with water and extracted with EtOAc three times. The organic phases were combined and dried with $MgSO_4$. The crude product was purified via column chromatography.

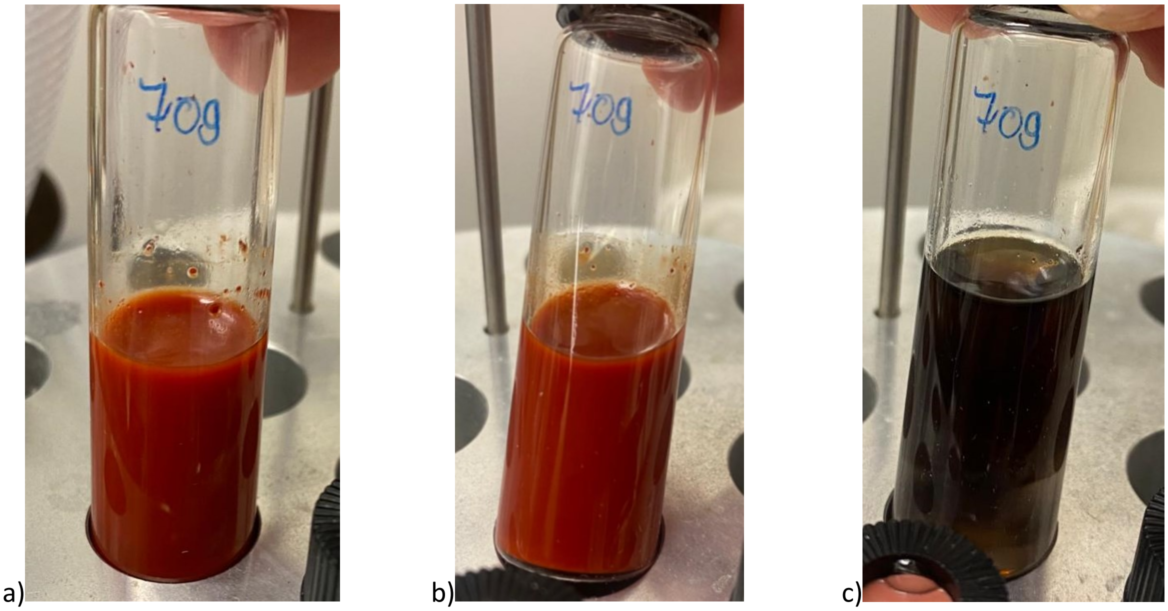

Figure S13. Typical cross coupling following the general procedure. a) after addition or ArCl in toluene. b) after 3 h. c) after aqueous workup.

1.4.2. Screening of reaction conditions

Table S1. Screening of TMEDA additive/BzK ratio.

For all screening reactions, isolated benzyl potassium synthesized via the procedure described in section 1.3.1 was used unless stated otherwise.

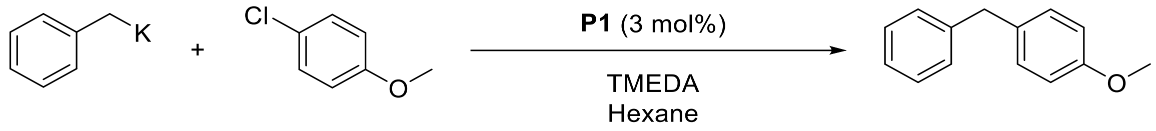

Entry	eq. BnK	eq. TMEDA	Yield (%)
1	1	1	40
2	2	1	46
3	3	1	41
4	4	1	12
5	5	1	38
6	1	2	87
7	1	3	16
8	1	4	12
9	1	5	11
10	1	0	26

Table S2. Catalyst screening.

Entry	Catalyst	eq. BnK	Yield (%)
1	**P2**	1	82
2	**P1**	1	83
3	pinkY-Allyl	1	12
4	joY-Allyl	1	8
5	**P4**	1	5
6	**P3**	1	17
7	Pd-PEPPSI-IPent	1	28
8	$Pd(PPh_3)_4$	1	6
9	Pd_2dba_3*PCy_3	1	2
10[1]	**P2**	3	89

[1]Without TMEDA

Table S3. Additive screening.

Entry	Solvent	eq. BnK	eq. TMEDA	Catalyst	Additive	Time	Yield (%)
1	Hexane	1	2	**P2**	Benzoquinone 1 eq.	3 h	0
2	Hexane	1	2	**P2**	$CuAc_2$ 1 eq.	3 h	48
3	Hexane	1	2	**P2**	$CuAc_2$ 2 eq.	3 h	46
4	Hexane	1	2	**P2**	$CuAc_2$ 3 mol%	3 h	18
5	Hexane	1	2	**P2**	AgAc 1 eq.	3 h	28
6	Hexane	1	2	**P2**	AgAc 3 mol%	3 h	23
7	Hexane	1	2	**P2**	$CuAc_2$/AgAc 0.25:0.25	3 h	39
8	Hexane	1	2	**P2**	$CuAc_2$/AgAc 0.5:0.5	3 h	60
9	Hexane	1	2	**P2**	$CuAc_2$/AgAc 1:1	3 h	11
10	Hexane	1	2	**P2**	$CuTf_2$ 5 mol%	3 h	74
11	Hexane	1	2	**P2**	$CuTf_2$ 10 mol%	3 h	80
12	Hexane	1	2	**P2**	$CuTf_2$ 15 mol%	3 h	79
13	Hexane	1	2	**P2**	$CuTf_2$ 20 mol%	3 h	74
14	Hexane	1	2	**P2**	$ZnCl_2$ 10 mol%	3 h	50
15	Hexane	1	2	**P2**	Air	3 h	15
16	Tech hex	1	2	**P2**	None	3 h	56
17	Hexane	1	2	None	$CuTf_2$ 1 eq.	3 h	5
18	Hexane	1	3	**P1**	$CuTf_2$ 5 mol%	3 h	82
19	Hexane	1	3	**P1**	$CuTf_2$ 10 mol%	3 h	79
20	Hexane	1	3	**P1**	$CuTf_2$ 15 mol%	3 h	85
21	Hexane	1	3	**P1**	None	3 h	54
22	Hexane	1	3	**P1**	NaI 1 eq.	3 h	50
23	Hexane	1	3	**P1**	NaI 10 mol%	3 h	70
24	Hexane	1	3	**P1**	Water (2 drops)	3 h	25
25	Hexane	1	3	**P1**	NaOTf 5 mol%	3 h	30
26	Hexane	1	3	**P1**	$MgOTf_2$ 10 mol%	3 h	31

Table S4. Speed of addition of ArCl stock solution.

Entry	Solvent	eq. BnK	eq. TMEDA	Catalyst	Speed of add.	Time	Yield (%)
1	Hexane	1	3	**P1**	Dropwise	3 h	59
2	Hexane	1	3	**P1**	Slow	3 h	39
3	Hexane	1	3	**P1**	Fast	3 h	45
4	Hexane	1	3	**P1**	Fast	24 h	48

Table S5. Control experiments of conditions.

Entry	Solvent	eq. BnK	eq. TMEDA	Catalyst	Additive	Time	Yield (%)
1	Hexane	1	3	**P1**	CuTf2 1 eq	3 h	54
2	Hexane	1	3	**P1**	CuTf2 10 mol%	3 h	30
3	Hexane	1	3	**P1**	None	3 h	35
4	Hexane	1	3	**P1**	None	3 h	41
5	Hexane	1	3	**P1 (3 mol%)**	None	3 h	36
6	Hexane	1	3	**P1 (5 mol%)**	None	3 h	59
7	Hexane	1	3	**P1 (10 mol%)**	None	3 h	20
8	Hexane	1	3	**P1 (3 mol%)**	CuTf2 10 mol%	3 h	30
9	Hexane	1	3	**P1 (5 mol%)**	CuTf2 10 mol%	3 h	75
10	Hexane	1	3	**P1 (10 mol%)**	CuTf2 10 mol%	3 h	19
11	Toluene	1	3	**P2**	None	3 h	70

As the control experiments verified unreliable results when using additives and donors after changing our initial batch of TMEDA, we opted to screen concentrations and amounts of organopotassium reagent with respect to the aryl halide in order to obtain a reliable protocol.

Table S6. Equivalent and concentration screening

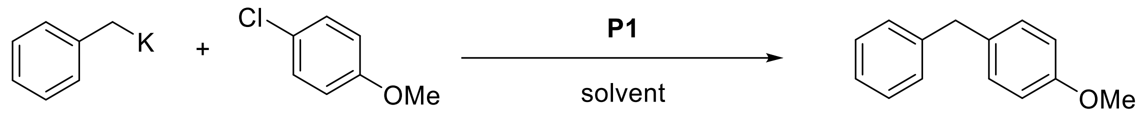

Entry	Solvent	eq. BnK	T (°C)	Time	Yield (%)
1	Hexane	1	65	3 h	19
2	Hexane	1	r.t.	3 h	19
3	Toluene	1	100	3 h	25
4	Toluene	3	100	3 h	31
5	Toluene	1	r.t.	16 h	26
6[1]	Toluene	1	r.t.	3 h	58
7	Toluene	3	r.t.	3 h	95
8	Toluene	4	r.t.	3 h	60
9	Hexane	3	r.t.	3 h	20
10	Hex/Tol	3	r.t.	3 h	82
11[2]	Toluene	3	r.t.	3 h	89
12[3]	Toluene	3	r.t.	3 h	89

[1] Concentrated mixture; 0.05 M. [2] BnK formed via deprotonation with *in-situ* generated TMS-CH_2-K (TMS-CH_2-Li 1 eq., KO*t*Bu 1 eq., toluene 5 eq. in hexane, 3 h at room temperature), subsequent filtration and solvent-switch to toluene for the cross coupling. [3] **P2** as catalyst

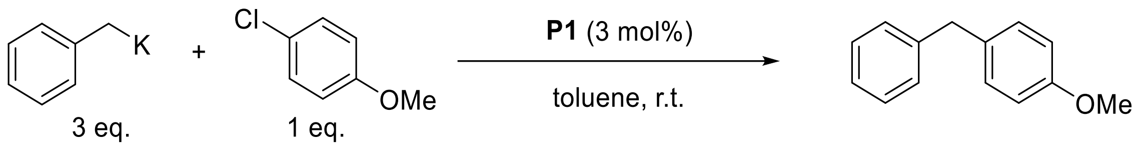

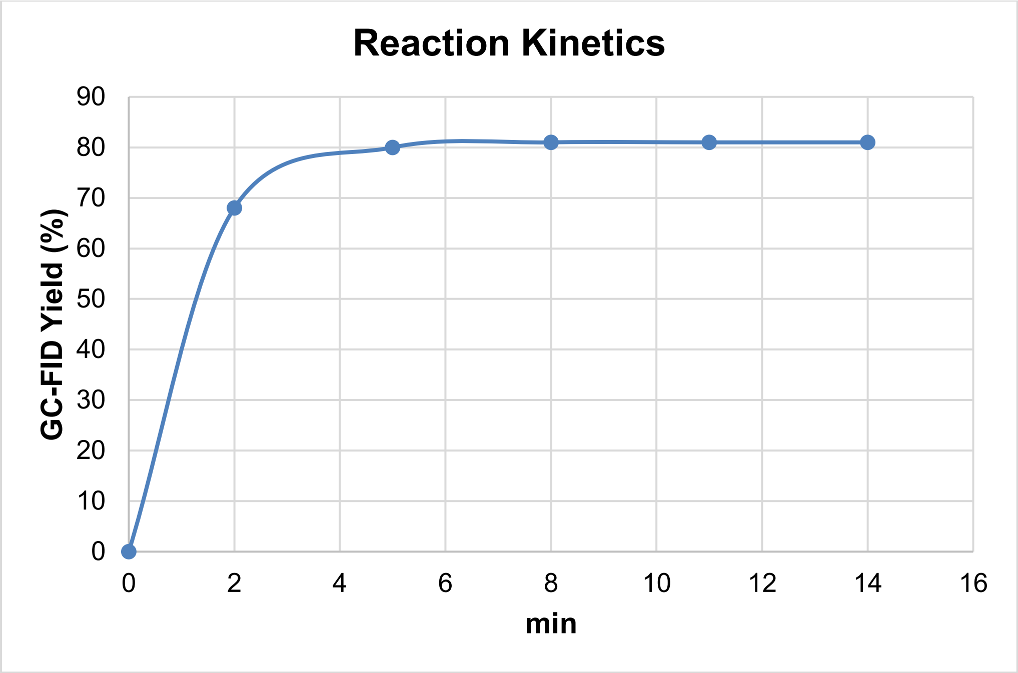

Figure S14. Kinetic monitoring of the reaction via GC-FID.

2. Synthesis and characterization of the catalysis products

2.1. Procedures and spectroscopic details

4-methoxydiphenylmethane (**4aa**).

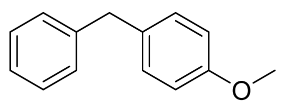

4aa

4aa was obtained with 92 % yield (91 mg, 0.5 mmol scale) via the general procedure for the direct cross-coupling **B** with 4-chloroanisole. The sample was purified via column chromatography (hexane/EtOAc 95:5). The analytical data is in accordance with the reported literature.

^{1}H-NMR (300 MHz, $CDCl_3$): δ = 7.32 – 7.25 (m, 2H), 7.23 – 7.15 (m, 3H), 7.11 (d, J = 8.8, Hz, 2H), 6.84 (d, J = 8.6 Hz, 2H), 3.93 (s, 2H), 3.79 (s, 3H) ppm. **^{13}C{^{1}H} NMR** (75 MHz, $CDCl_3$): δ = 158.1, 141.7, 133.4, 130.0, 128.9, 128.5, 126.1, 114.0, 55.4, 41.1 ppm. **MS (EI):** m/z (%) = 198.1 (100 [M^+]), 197.1 (42), 167.1 (40), 121.1 (33), 165.1 (28), 153.1 (20), 152.1 (19), 183.1 (16), 91.1 (15), 199.1 (15), 77.0 (14), 166.1 (11).

Reference: L. Chahen, H. Doucet, M. Santelli, *Synlett*. **2003**, 1668 – 1672.

2-benzylnaphthalene (**4ab**).

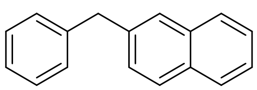

4ab

4ab was obtained with 99 % (108 mg, 0.5 mmol scale) yield via the general procedure for the direct cross-coupling **A** with 2-chloronaphthalene. The sample was purified via column chromatography (hexane/EtOAc 100:0). The analytical data is in accordance with the reported literature.

^{1}H-NMR (300 MHz, $CDCl_3$): δ = 7.96 – 7.84 (m, 2H), 7.79 – 7.74 (m, 2H), 7.62 – 7.60 (m, 1H), 7.46 – 7.38 (m, 2H), 7.32 – 7.27 (m, 2H), 7.23 – 7.18 (m, 3H), 4.12 (s, 2H) ppm. **^{13}C{^{1}H} NMR** (75 MHz, $CDCl_3$): δ = 141.1, 138.7, 138.5, 133.8, 133.7, 132.8, 132.2, 129.1, 128.6, 128.6, 128.3, 128.2, 127.8, 127.7, 127.7, 127.7, 127.6, 127.2, 126.5, 126.2, 126.2, 126.1, 126.1, 125.8, 125.4, 42.2 ppm. **MS (EI):** m/z (%) = 218.09 (100 [M^+]), 217.09 (72), 202.05 (34), 215.07 (32), 203.05 (30), 219.1 (17), 141.03 (16), 115.02 (14), 107.89 (11), 216.08 (10).
Reference: T. Suga, Y. Ukaji, *Org. Lett.* **2018**, 7846 – 7850.

4-benzyltoluene (**4ac**).

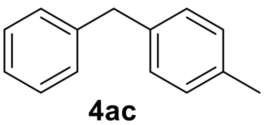

4ac

4ac was obtained with 99 % (91 mg, 0.5 mmol scale) as a mixture containing bibenzyl via the general procedure for the direct cross-coupling **B** with 4-chlorotoluene. The analytical data is in accordance with the reported literature.

^{1}H-NMR (300 MHz, $CDCl_3$): δ = 7.50 – 7.43 (m, 1H), 7.29 – 7.22 (m, 2H), 7.20 – 7.14 (m, 3H), 7.07 (s, 3H), 3.93 (s, 2H), 2.30 (s, 3H) ppm **^{13}C-NMR** (75 MHz, $CDCl_3$): δ = 141.5, 138.2, 135.6, 129.2, 129.0, 128.9, 126.9, 126.1, 46.6, 21.1 ppm. **MS (EI):** m/z (%) = 167.1 (100), 182.1 (73 [M^+]), 165.1 (37), 104 (27), 166.1 (19), 152 (16), 168.1 (14), 91 (14), 181.1 (12), 89 (11), 183.1 (11).

Reference: S. K. Yen, L. L. Koh, H. V. Huynh, T. S. A. Hor, *Eur. J. Inorg. Chem.* **2009**, 4288–4297.

1-(1,1-dimethylethyl)-4-(phenylmethyl)benzene (**4ad**).

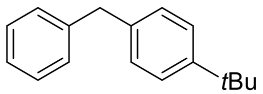

4ad

4ad was obtained with 99 % (111 mg, 0.5 mmol scale) yield via the general procedure for the direct cross-coupling **B** with 1-chloro-4-(tertbutyl)benzene. The sample was purified via column chromatography (hexane/EtOAc 100:0). The analytical data is in accordance with the reported literature.

^{1}H-NMR (300 MHz, $CDCl_3$): δ = 7.28 (dt, J = 4.2, 2.2 Hz, 2H), 7.26 – 7.21 (m, 2H), 7.19 – 7.14 (m, 3H), 7.12 – 7.05 (m, 2H), 3.92 (s, 2H), 1.27 (s, 9H) ppm. **^{13}C{^{1}H} NMR** (75 MHz, $CDCl_3$): δ = 148.9, 141.4, 138.2, 129.1, 128.6, 128.5, 126.1, 126.0, 125.4, 41.5, 34.5, 31.5 ppm. **MS (EI):** m/z (%) = 209.2 (100), 91 (51), 224.2 (35 [M^+]), 210.2 (22), 165.1 (12).

Reference: H. Yang, X. Han, G. Li, Z. Ma, Y. Hao, *J. Phys. Chem. C.* **2010**, 22221 – 22229.

2-(phenylmethyl)-1,1'-biphenyl (**4ae**).

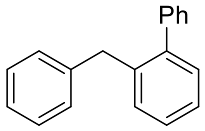

4ae

4ae was obtained with 80 % (202 mg, 1 mmol scale) yield via the general procedure for the direct cross-coupling **B** with 2-bromobiphenyl. The sample was purified via column chromatography (hexane/EtOAc 100:0). The analytical data is in accordance with the reported literature.

^{1}H-NMR (300 MHz, $CDCl_3$): δ = 7.40 – 7.11 (m, 12H), 7.08 – 6.92 (m, 2H), 3.95 (s, 2H) ppm. **^{13}C{^{1}H} NMR** (75 MHz, $CDCl_3$): δ = 142.4, 141.7, 141.6, 138.3, 130.4, 130.2, 129.4, 129.0, 128.3, 128.1, 127.6, 127.0, 126.3, 125.9, 39.1 ppm. **MS (EI):** m/z (%) = 244.1 (100 [M^+]), 165.1 (60), 166.1 (28), 243.1 (23), 167.1 (22), 245.1 (21), 229.1 (20), 228.1 (12), 152 (12).

Reference: C-R. Chen, S. Zhou, D. B. Biradar, H-M. Gau, *Adv. Synth. Catal.* **2010**, 352, 1718 – 1727.

1-fluoro-4-(phenylmethyl)-benzene (**4af**).

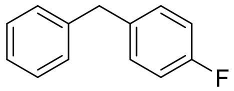

4af

4af was obtained with 76 % (71 mg, 0.5 mmol scale) yield via the general procedure for the direct cross-coupling **B** with 4-chloro fluorobenzene. The sample was purified via column chromatography (hexane/EtOAc 99:1). The analytical data is in accordance with the reported literature.

^{1}H-NMR (300 MHz, $CDCl_3$): δ = 7.32 – 7.23 (m, 2H), 7.23 – 7.18 (m, 1H), 7.18 – 7.12 (m, 3H), 7.11 (d, *J* = 2.3 Hz, 1H), 7.01 – 6.91 (m, 2H), 3.94 (s, 2H) ppm. **^{13}C{^{1}H} NMR** (75 MHz, $CDCl_3$): δ = 161.5 (d, *J* = 243.9 Hz), 141.0, 136.9 (d, *J* = 3.1 Hz), 130.4 (d, *J* = 7.8 Hz), 128.9, 128.6, 126.3, 115.3 (d, *J* = 21.3 Hz), 41.2 ppm. **MS (EI):** m/z (%) = 186.1 (100 [M^+]), 185.1 (78), 165.1 (36), 183 (30), 109 (17), 187.1 (14), 171 (12), 91.1 (12), 166.1 (10).

Reference: M. J. Burns, I. J. S. Fairlamb, A. R. Kapdi, P. Sehnal, R. J. K. Taylor, *Org. Lett.* **2007**, 9, 26, 5397 – 5400.

4-benzylbenzotrifluoride (**4ag**).

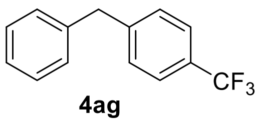

4ag

4ag was obtained with 64 % (75 mg, 0.5 mmol scale) yield via the general procedure for the direct cross-coupling **B** with 1-chloro-4-(trifluoromethyl)benzene. The sample was purified via column chromatography (hexane/EtOAc 99:1). The analytical data is in accordance with the reported literature.

^{1}H-NMR (300 MHz, $CDCl_3$): δ = 7.51 (d, *J* = 8.1 Hz, 2H), 7.33 – 7.24 (m, 4H), 7.24 – 7.20 (m, 1H), 7.16 (ddt, *J* = 7.4, 1.4, 0.7 Hz, 2H), 4.01 (s, 2H) ppm. **^{13}C{^{1}H} NMR** (75 MHz, $CDCl_3$): δ = 145.3, 140.1, 129.3, 129.0, 128.8, 128.9 – 128.3 (m), 126.6, 125.5 (q, *J* = 3.8 Hz), 122.6, 41.8 ppm. **MS (EI):** m/z (%) = 167.1 (100), 236.1 (73 [M^+]), 165.1 (42), 166.1 (19), 168.1 (15), 91 (14), 152.1 (13), 235.1 (12), 237.1 (11).

Reference: C. I. Someya, E. Irran, S. Enthaler, *Asian J. Org. Chem.* **2012**, 1, 322 – 326.

1-benzylnaphthalene (**4ah**).

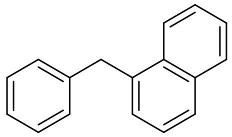

4ah

4ah was obtained with 76 % (165 mg, 1 mmol scale) yield via the general procedure for the direct cross-coupling **B** with 1-chloronaphthalene. The sample was purified via column chromatography (hexane/EtOAc 100:0). The analytical data is in accordance with the reported literature.

^{1}H-NMR (300 MHz, $CDCl_3$): δ = 7.99 (dddd, J = 7.5, 5.3, 2.6, 1.2 Hz, 1H), 7.92 – 7.80 (m, 1H), 7.76 (d, *J* = 8.2 Hz, 1H), 7.50 – 7.37 (m, 3H), 7.34 – 7.07 (m, 7H), 4.45 (s, 2H) ppm. **^{13}C{^{1}H} NMR** (75 MHz, $CDCl_3$): δ = 140.7, 136.7, 134.0, 132.2, 128.8, 128.8, 128.6, 127.4, 127.3, 126.2, 126.1, 125.7, 124.4, 39.1 ppm. **MS (EI):** m/z (%) = 218.1 (100 [M^+]), 217.1 (73), 202.1 (35), 215.1 (33), 203.1 (32), 219.1 (18), 141 (15), 115 (12), 216.1 (11), 107.9 (10).

Reference: M. Y. Tsang, C. Vinas, F. Teixidor, J. G. Planas, N. Conde, R. SanMartin, M. T. Herrero, E. Dominguez, A. Lledos, P. Vidossich, D. Choquesillo-Lazarte, *Inorg. Chem.* **2014**, 53, 17, 9284 – 9295.

1,3,5-trimethyl-2-(phenylmethyl)-benzene (**4ai**).

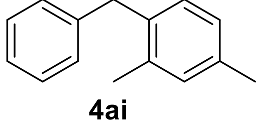

4ai

4ai was obtained with 45 % (48 mg, 0.5 mmol scale) yield via the general procedure for the direct cross-coupling **B** with mesitylbromide. The sample was purified via column chromatography (hexane/EtOAc 100:0). The analytical data is in accordance with the reported literature.

^{1}H-NMR (300 MHz, $CDCl_3$): δ = 7.24 – 7.12 (m, 3H), 7.01 (ddt, J = 7.0, 2.1, 1.0 Hz, 2H), 6.89 (s, 2H), 4.02 (s, 2H), 2.29 (s, 3H), 2.21 (s, 6H) ppm. **^{13}C{^{1}H} NMR** (75 MHz, $CDCl_3$): δ = 139.5, 137.1, 135.8, 133.9, 129.0, 128.4, 128.0, 125.7, 34.8, 21.0, 20.2 ppm **MS (EI):** m/z (%) = 195.1 (100), 210.1 (70 [M^+]), 180.1 (23), 165.1 (20), 132.1 (18), 196.1 (17), 133.1 (14), 179.1 (14), 91 (14), 178.1 (14), 211.2 (12).

Reference: B. Li, K. Leng, Y. Zhang, J. J. Dynes, J. Wang, Y. Hu, D. Ma, Z. Shi, L. Zhu, D. Zhang, Y. Sun, M. Chrzanowski, S. Ma, *J. Am. Chem. Soc.* **2015**, 137, 12, 4243–4248

1-methyl-2-(phenylmethyl)-benzene (**4aj**).

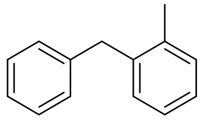

4aj

4aj was obtained with 70 % (64 mg, 0.5 mmol scale) yield via the general procedure for the direct cross-coupling **B** with 2-chlorotoluene. The sample was purified via column chromatography (hexane/EtOAc 100:0). The analytical data is in accordance with the reported literature.

^{1}H-NMR (300 MHz, $CDCl_3$): δ = 7.25 (tq, *J* = 6.3, 1.4 Hz, 2H), 7.19 – 7.17 (m, 1H), 7.17 – 7.12 (m, 3H), 7.12 – 7.06 (m, 3H), 3.97 (s, 2H), 2.22 (s, 3H) ppm. **^{13}C{^{1}H} NMR** (75 MHz, $CDCl_3$): δ = 140.5, 139.0, 136.7, 130.4, 130.0, 128.8, 128.5, 126.5, 126.1, 126.0, 39.5, 19.8 ppm. **MS (EI):** m/z (%) = 167.1 (100), 182.1 (73 [M$^+$]), 165.1 (37), 104 (27), 166.1 (19), 152 (16), 168.1 (14), 91 (14), 181.1 (12), 89 (11), 183.1 (11).

Reference: S. W. Seo, H. S. Song, J. H. Song, G-H. Kim, I. In, C. P. Park, *Tetrahedron Letters*, **2015**, 56, 2795 – 2798.

1-(4-benzylphenyl)ethanone (**4ak**).

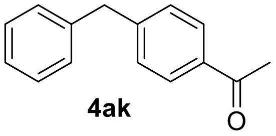

4ak

4ak was obtained with 21 % (45 mg, 1 mmol scale) yield via the general procedure for the direct cross-coupling **B** with 1-(4-chlorophenyl)ethanone. The sample was purified via column chromatography (hexane/EtOAc 85:15). The analytical data is in accordance with the reported literature.

^{1}H-NMR (300 MHz, $CDCl_3$): δ = 7.89 (d, *J* = 8.5 Hz, 2H), 7.32 – 7.25 (m, 4H), 7.25 – 7.16 (m, 3H), 4.04 (s, 2H), 2.58 (s, 3H) ppm. **^{13}C{^{1}H} NMR** (75 MHz, $CDCl_3$): δ = 197.9, 146.9, 140.1, 135.3, 129.8, 129.2, 129.0, 129.0, 128.7, 128.7, 126.5, 42.0, 26.7, 1.1 ppm. **MS (EI):** m/z (%) = 195.06 (100), 210.08 (41 [M$^+$]), 165.05 (38), 152.03 (24), 196.05 (15), 166.06 (13), 167.05 (10).

Reference: C-M. Hsu, S-C. Lee, H-E. Tsai, Y-T. Tsao, C-L. Chan, S. Minoza, Z-N. Tsai, L-Y. Li, H-H. Liao, *J. Org. Chem.* **2022**, 87, 5, 3799 – 3803

4-benzyl-*N,N*-diisopropylbenzamide (**4al**).

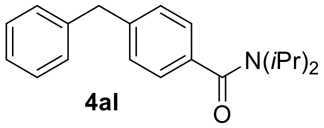

4al

4al was obtained with 45 % (66 mg, 0.5 mmol scale) yield via the general procedure for the direct cross-coupling **B** with 4-chloro-*N,N*-diisopropylbenzamide. The sample was purified via column chromatography (hexane/EtOAc 80:20).

^{1}H-NMR (300 MHz, $CDCl_3$): δ = 7.33 – 7.15 (m, 9H), 3.99 (s, 2H), 3.94 – 3.36 (m, 2H), 1.79 – 1.00 (m, 12H) ppm. **^{13}C{^{1}H} NMR** (75 MHz, $CDCl_3$): δ = 171.21, 141.90, 140.76, 129.15, 129.07, 128.65, 126.37, 126.10, 41.89, 20.92 ppm. **MS (EI):** m/z (%) = 195.1 (100), 252.1 (32), 165.1 (29), 152 (21), 196.1 (20), 295.2 (15 [M$^+$]), 166.1 (11).

HRMS-QTOF (m/z) = [R(iPr)N$^-$] calcd for $C_{17}H_{18}NO^-$, 252.1394; found, 252.1393.

2-methoxy-6-(phenylmethyl)pyridine (**4am**).

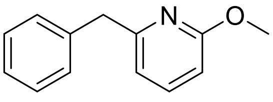

4am

4am was obtained with 45 % GC-conversion as a mixture via the general procedure for the direct cross-coupling **B** with 2-chloro-6-methoxypyridine. The identity of the product was verified via GC-MS.

MS (EI): m/z (%) = 198.1 (100), 199.09 (55 [M^+]), 183.05 (20), 184.06 (16), 91.05 (14), 154.05 (11).

2-[(4-methoxyphenyl)methyl]quinoline (**4ba**).

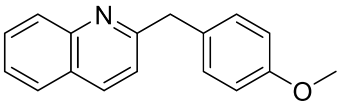

4ba

4ba was obtained with 99 % (172 mg, 700 mmol scale) yield via the general procedure for the direct cross-coupling **B** with 4-chloroanisole. The sample was purified via column chromatography (hexane/EtOAc 99:1). The analytical data is in accordance with the reported literature.

^{1}H-NMR (300 MHz, $CDCl_3$): δ = 8.12 – 7.98 (m, 2H), 7.80 – 7.64 (m, 2H), 7.49 (ddd, *J* = 8.1, 6.9, 1.2 Hz, 1H), 7.24 – 7.17 (m, 3H), 6.87 – 6.80 (m, 2H), 4.28 (s, 2H), 3.77 (s, 3H) ppm. **^{13}C{^{1}H} NMR** (75 MHz, $CDCl_3$): δ = 161.7, 158.4, 147.9, 136.6, 131.4, 130.3, 129.5, 129.1, 127.6, 126.9, 126.0, 121.6, 114.1, 55.3, 44.8 ppm. **MS (EI):** m/z (%) = 248.11 (100), 249.11 (95 [M^+]), 234.09 (71), 204.07 (20), 121.05 (20), 250.1 (18), 217.08 (17), 206.09 (15), 128.03 (14), 235.09 (14), 77.04 (13), 205.08 (10), 233.08 (10).

Reference: M. Puthanveedu, V. Polychronidou, A. P. Antonchick, *Org. Lett.* **2019**, 21, 9, 3407 – 3411.

2-(2-Naphthalenylmethyl)quinoline (**4bb**).

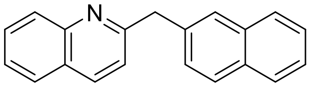

4bb

4bl was obtained with 99 % (132 mg, 0.5 mmol scale) yield via the general procedure for the direct cross-coupling **B** with 2-chloronaphthalene. The sample was purified via column chromatography (hexane/EtOAc 99:1). The analytical data is in accordance with the reported literature.

^{1}H-NMR (300 MHz, $CDCl_3$): δ = 8.11 (dq, *J* = 8.4, 0.9 Hz, 1H), 8.00 (dd, *J* = 8.5, 0.8 Hz, 1H), 7.79 – 7.72 (m, 5H), 7.71 – 7.67 (m, 1H), 7.49 (ddd, *J* = 8.1, 6.9, 1.2 Hz, 1H), 7.45 – 7.37 (m, 3H), 7.27 – 7.19 (m, 1H), 4.50 (s, 2H) ppm. **^{13}C{^{1}H} NMR** (75 MHz, $CDCl_3$): δ = 161.2, 148.0, 136.8, 136.6, 133.7, 132.3, 129.6, 129.6, 129.1, 128.4, 128.1, 127.7, 127.7, 127.6, 126.9, 126.2, 126.1, 125.6, 121.7, 45.8 ppm. **MS (EI):** m/z (%) = 268.14 (100), 269.12 (66 [M^+], 267.11 (34), 133.57 (20), 270.11 (13), 266.11 (13), 115.04 (13).

Reference: D-W. Wang, X-B. Wang, D-S. Wang, S-M. Lu, Y-G. Zhou, Y-X. Li, *J. Org. Chem.* **2009**, 74, 7, 2780 – 2787.

2-[(4-methoxyphenyl)methyl]quinoline (**4ca**).

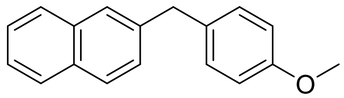

4ca

4ca was obtained with 80 % (198 mg, 1 mmol scale) yield via the general procedure for the direct cross-coupling **B** with 4-chloroanisole. The sample was purified via column chromatography (hexane/EtOAc 99:1). The analytical data is in accordance with the reported literature.

^{1}H-NMR (300 MHz, $CDCl_3$): δ = 7.84 – 7.71 (m, 3H), 7.61 (dt, *J* = 2.3, 1.0 Hz, 1H), 7.53 – 7.35 (m, 2H), 7.30 (dd, *J* = 8.4, 1.8 Hz, 1H), 7.24 – 7.09 (m, 2H), 6.89 – 6.72 (m, 2H), 4.08 (s, 2H), 3.78 (s, 3H) ppm. **^{13}C{^{1}H} NMR** (75 MHz, $CDCl_3$): δ = 158.1, 139.2, 133.7, 133.2, 132.1, 130.1, 128.2, 128.1, 127.7, 127.7, 127.6, 127.0, 126.0, 125.4, 125.4, 114.0, 55.4, 41.3 ppm. **MS (EI):** m/z (%) = 248.13 (100 [M$^+$]), 217.11 (40), 247.12 (31), 215.1 (31), 202.08 (29), 249.13 (20), 203.09 (20), 121.05 (17), 216.1 (13), 233.1 (12), 141.06 (12), 101.05 (11).

Reference: P. Maity, D. M. Shacklady-McAtee, G. P. A. Yap, E. R. Sirianni, M. P. Watson, *J. Am. Chem. Soc.* **2013**, 135, 1, 280 – 285.

1-(1,1-Dimethylethyl)-4-[(4-methoxyphenyl)methyl]benzene (**4da**).

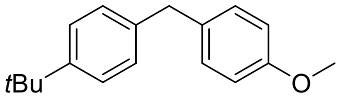

4da

4da was obtained with 91 % (162 mg, 0.7 mmol scale) yield via the general procedure for the direct cross-coupling **B** with 4-chloroanisole. The sample was purified via column chromatography (hexane/EtOAc 99:1). The analytical data is in accordance with the reported literature.

^{1}H-NMR (300 MHz, $CDCl_3$): δ = 7.34 – 7.19 (m, 2H), 7.17 – 7.04 (m, 4H), 6.88 – 6.76 (m, 2H), 3.89 (s, 2H), 3.77 (s, 3H), 1.29 (s, 9H) ppm. **^{13}C{^{1}H}-NMR** (75 MHz, $CDCl_3$): δ = 158.0, 148.8, 138.6, 133.5, 130.0, 128.5, 125.4, 113.9, 55.3, 40.6, 34.5, 31.5 ppm. **MS (EI):** m/z (%) = 239.18 (100), 254.16 (53 [M$^+$]), 121.07 (48), 240.15 (25), 197.09 (23), 255.16 (11).

Reference: G. Lu, R. Li, Z. Shen, Q. Wu, H. Sun, *Appl. Organomet. Chem.* **2020**, 34, e5741.

1-[(4-methoxyphenyl)methyl]-3,5-dimethylbenzene (**4eb**).

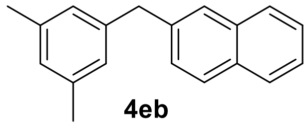

4eb

4eb was obtained with 80 % (196 mg, 1 mmol scale) yield via the general procedure **B** with 2-chloronaphthalene. The sample was purified via column chromatography (hexane/EtOAc 100:0). The analytical data is in accordance with the reported literature.

^{1}H-NMR (400 MHz, $CDCl_3$): δ = 7.81 – 7.73 (m, 3H), 7.64 (d, *J* = 1.7 Hz, 1H), 7.49 – 7.39 (m, 2H), 7.32 (dd, *J* = 8.4, 1.8 Hz, 1H), 6.85 (s, 3H), 4.06 (s, 2H), 2.27 (s, 6H) ppm.
MS (EI): m/z (%) = 211.1 (100), 226.2 (99 [M$^+$]), 195.1 (24), 121 (22), 225.2 (22), 165.1 (20), 118.1 (18), 227.2 (17), 212.1 (17), 196.1 (14), 179.1 (12), 152.1 (12), 77 (11), 180.1 (10).

Reference: P. Guo, K. Wang, W-J. Jin, H. Xie, L. Qi, X-Y. Liu, X-Z. Shu, *J. Am. Chem. Soc.* **2021**, *143*, 513 – 523.

2-((5,5,8,8-tetramethyl-5,6,7,8-tetrahydronaphthalen-2-yl)methyl)naphthalene (**4fb**).

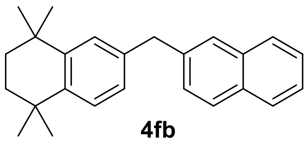

4fb

4fb was obtained with 50 % GC-conversion with the general procedure for the direct cross-coupling **A** with 2-chloronaphthalene. The identity of the product was verified via GC-MS.

MS (EI): m/z (%) = 141.1 (100), 313.2 (72), 328.2 (29 [M^+]), 314.2 (20), 142.1 (15), 115 (11).

1-[(4-methoxyphenyl)methyl]naphthalene (**4ga**).

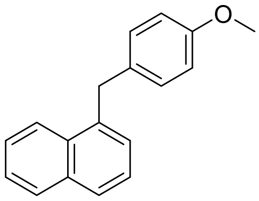

4ga

4ga was obtained with 50 % GC-conversion via the general procedure for the direct cross-coupling **B** with 4-chloroanisole. The identity of the product was verified via GC-MS.

MS (EI): m/z (%) = 248.1 (100 [M^+]), 217.1 (38), 247.1 (31), 215.1 (31), 202.1 (27), 249.1 (19), 203.1 (18), 233.1 (15), 121 (15), 216.1 (14), 141 (10).

Reference: D. Zhang, Z. Xu, T. Tang, L. Le, C. Wang, N. Kambe, R. Qiu, *Org. Lett.* **2022**, *24*, 3155 – 3160.

2-[(4-methoxyphenyl)methyl]-6-methylpyridine (**4ha**).

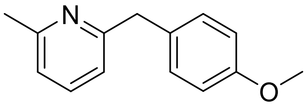

4ha

4ha was obtained with 45 % (96 mg, 1 mmol scale) yield via the general procedure for the direct cross-coupling **A** with 4-chloroanisole. The sample was purified via column chromatography (hexane/EtOAc 90:10). The analytical data is in accordance with the reported literature.

^{1}H-NMR (300 MHz, CDCl3): δ = 7.45 (t, J = 7.7 Hz, 1H), 7.22 – 7.15 (m, 2H), 6.97 (d, J = 7.6 Hz, 1H), 6.87 – 6.81 (m, 3H), 4.09 (s, 2H), 3.78 (s, 3H), 2.56 (s, 3H) ppm.

^{13}C{^{1}H} NMR (75 MHz, CDCl3): δ = 160.8, 158.3, 157.8, 137.0, 131.7, 130.2, 128.8, 127.3, 120.8, 120.0, 114.0, 55.3, 43.7, 24.5 ppm.

MS (EI): m/z (%) = 198.11 (100), 213.14 (92 [M^+]), 212.13 (74), 121.07 (26), 170.1 (20), 199.11 (16), 214.13 (15), 77.06 (14), 65.05 (13), 168.09 (12), 154.07 (12), 78.04 (12), 181.09 (11).

Reference: M. Takimoto, M. Liu, M. Nishiura, Z. Hou, *ACS Catal.* **2022**, *12*, 13792 – 13804.

9-(4-methoxyphenyl)-9*H*-xanthene (**4ia**).

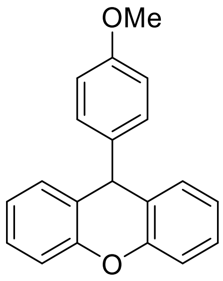

4ia

4ia was obtained with 53 % GC-conversion via the general procedure for the direct cross-coupling **A** with 4-chloroanisole. The identity of the product was verified via GC-MS.

MS (EI): m/z (%) = 181.03 (100), 288.08 (32 [M$^+$]), 287.07 (19), 182.04 (14). 152.02 (11).

Reference: W. Chen, Z. Xie, H. Zheng, H. Lou, L. Liu, *Org. Lett.* **2014**, *16*, 5988 – 5991.

9-(2-naphthalenyl)-9*H*-xanthene (**4ib**).

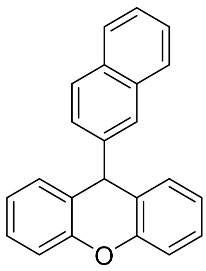

4ib

4ib was obtained with 71 % (110 mg, 0.5 mmol scale) yield via the general procedure for the direct cross-coupling **A** with 2-chloronaphthalene. The sample was purified via column chromatography (hexane/EtOAc 90:10). The analytical data is in accordance with the reported literature.

^{1}H-NMR (300 MHz, $CDCl_3$): δ = 7.84 – 7.68 (m, 4H), 7.52 – 7.38 (m, 2H), 7.25 (d, *J* = 0.9 Hz, 1H), 7.24 – 7.12 (m, 4H), 7.05 (ddt, *J* = 7.7, 1.5, 0.7 Hzm 2H), 6.94 (ddd, *J* = 7.7, 6.8, 1.6 Hz, 2H), 5.42 (s, 1H) ppm. **^{13}C{^{1}H} NMR** (75 MHz, $CDCl_3$): δ = 151.1, 143.6, 133.4, 132.4, 130.0, 129.0, 128.1, 127.9, 127.8, 126.9, 126.8, 126.3, 125.9, 124.1, 123.3, 116.7, 44.6 ppm. **MS (EI):** m/z (%) = 181.03 (100), 308.08 (30 [M$^+$]), 182.04 (14), 307.07 (14), 152.05 (10).

Reference: C. Li, R. Ding, H-Y. Guo, S. Xia, L. Shu, P-L. Wang, H. Li *Green Chem.* **2022**, 24, 7883 – 7888.

1-methoxy-4-[(2*E*)-3-phenyl-2-propen-1-yl]benzene (**4ja**).

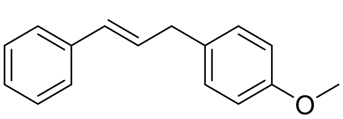

4ja

4ja was obtained with minor impurities of different isomers with 89 % (99 mg, 0.5 mmol scale) yield via the general procedure for the direct cross-coupling **A** with 4-chloroanisole. The sample was purified via column chromatography (hexane/EtOAc 95:5). The analytical data is in accordance with the reported literature.

^{1}H-NMR (300 MHz, $CDCl_3$): δ = 7.39 – 7.28 (m, 5H), 7.23 – 7.10 (m, 3H), 6.89 – 6.81 (m, 3H), 6.48 – 6.16 (m, 2H), 3.80 (s, 3H), 3.50 (d, J = 6.2 Hz, 2H) ppm. **^{13}C{^{1}H} NMR** (75 MHz, $CDCl_3$): δ = 158.2, 137.6, 132.3, 130.8, 129.8, 129.7, 128.6, 127.1, 126.2, 114.0, 55.4, 38.6, 33.8 ppm. **MS (EI):** m/z (%) = 224.1 (100 [M$^+$]), 115.0 (61), 193.1 (30), 223.1 (29), 121.1 (28), 91.0 (27), 209.1 (22), 77.0 (19), 178.1 (19), 165.1 (18), 116.1 (18), 225.1 (17), 78.0 (11), 89.0 (10).

Reference: I. Fernandez, R. Hermatschweiler, F. Breher, P. S. Pregosin, L. F. Veiros, M. J. Calhorda, *Angew. Chem. Int. Ed.* **2006**, *45*, 6386 – 6391.

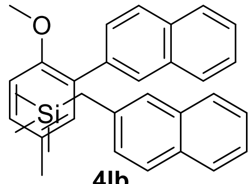

4lb
4kb

2-[(trimethylsilyl)methyl]naphthalene (**4kb**).

((Trimethylsilyl)methyl)potassium was synthesized via metal exchange reaction of TMS-CH_2-Li with KO*t*Bu and **4kb** was obtained with 62 % (33 mg, 0.25 mmol scale) yield via the general procedure for the direct cross-coupling **A** with 2-chloronaphthalene. The sample was purified via column chromatography (hexane/EtOAc 99:1). The analytical data is in accordance with the reported literature.

^{1}H-NMR (300 MHz, $CDCl_3$): δ = 7.81 – 7.66 (m, 3H), 7.63 – 7.56 (m, 1H), 7.52 – 7.41 (m, 1H), 7.39 – 7.29 (m, 1H), 7.26 – 7.12 (m, 1H), 2.24 (s, 2H), 0.02 (s, 9H) ppm.

Reference: M. Tobisu, T. Takahira, N. Chatani, *Org. Lett.* **2015**, 17,17, 4352 – 4355.

2-(2-methoxy-5-methylphenyl)naphthalene (**4lb**).

4lb was obtained with 98 % (158 mg, 0.65 mmol scale) yield via the general procedure for the direct cross-coupling **A** with 2-chloronaphthalene. The sample was purified via column chromatography (hexane/EtOAc 100:0). The analytical data is in accordance with the reported literature.

^{1}H-NMR (300 MHz, $CDCl_3$): δ = 7.97 – 7.92 (m, 1H), 7.85 (dd, J = 9.1, 3.5 Hz, 3H), 7.68 (dd, J = 8.5, 1.7 Hz, 1H), 7.46 (tt, J = 5.9, 4.7 Hz, 2H), 7.28 – 7.21 (m, 1H), 7.14 (dd, J = 8.4, 2.3 Hz, 1H), 6.91 (d, J = 8.3 Hz, 1H), 3.79 (s, 3H), 2.36 (s, 3H) ppm. **^{13}C{^{1}H} NMR** (75 MHz, $CDCl_3$): δ = 154.7, 136.4, 133.5, 132.5, 131.9, 130.5, 130.2, 129.1, 128.2, 128.1, 127.7, 127.2, 126.0, 125.8, 111.5, 55.9, 20.6, 1.1 ppm. **MS (EI):** m/z (%) = 248.1 (100 [M^+]), 218.1 (53), 233.1 (32) **HRMS-QTOF** (m/z) = [$C_{18}H_{16}O$] calcd for $C_{18}H_{16}O$, 248.1201; found, 248.1204.

2-(2,6-dimethoxyphenyl)naphthalene (**4mb**).

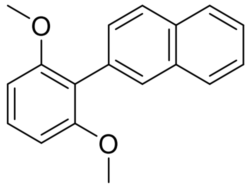

4mb

4mb was obtained with 98 % GC-conversion via the general procedure for the direct cross-coupling **A** with 2-chloronaphthalene. The identity of the product was verified via GC-MS.

MS (EI): m/z (%) = 264.1 (100 [M^+]), 249.1 (33), 234.0 (29), 265.1 (21), 218.0 (18), 152.0 (17), 189.0 (15), 178.0 (15), 263.1 (12).

Reference: S. Asako, H. Nakajima, K. Takai, *Nat. Catal.* **2019**, 2, 297 – 303.

4-methoxybiphenyl (**4na**).

Phenyl potassium was prepared via Schlosser's base with benzene as the solvent. **4na** was obtained with 81 % (150 mg, 1 mmol scale) yield via the general procedure for the direct cross-coupling **A** with 4-chloroanisole. The sample was purified via column chromatography (hexane/EtOAc 99:1). The analytical data is in accordance with the reported literature.

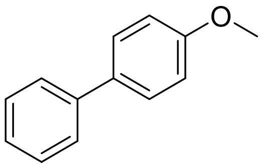

4na

^{1}H-NMR (300 MHz, $CDCl_3$): δ = 7.59 – 7.50 (m, 4H), 7.42 (tq, J = 6.8, 0.7 Hz, 2H), 7.35 – 7.27 (m, 1H), 7.00 – 6.97 (m, 2H), 3.86 (s, 3H) ppm. **^{13}C{^{1}H} NMR** (75 MHz, $CDCl_3$): δ = 159.2, 140.9, 133.9, 128.8, 128.3, 126.8, 126.8, 114.3, 55.4 ppm. **MS (EI):** m/z (%) = 184.1(100 [M$^+$]), 169.1 (50), 141.1 (48), 115.0 (37), 185.1 (15), 139.1 (13).

Reference: C. Wu, S. P. McCollom, Z. Zheng, J. Zhang, S-C. Sha, M. Li, P. J. Walsh, N. C. Tomson, *ACS Catal.* **2020**, *10*, 7934 – 7944.

2-phenylnaphthalene (**4nb**).

Phenyl potassium was prepared via Schlosser's base with benzene as the solvent. **4nb** was obtained with 78 % (160 mg, 1 mmol scale) yield via the general procedure for the direct cross-coupling **A** with 4-chloroanisole. The sample was purified via column chromatography (hexane/EtOAc 100:0). The analytical data is in accordance with the reported literature.

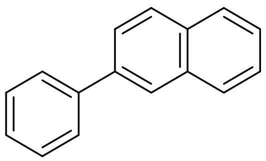

4nb

^{1}H-NMR (400 MHz, $CDCl_3$): δ = 7.97 (d, *J* = 1.8 Hz, 1H), 7.87 – 7.74 (m, 3H), 7.71 – 7.61 (m, 3H), 7.47 – 7.36 (m, 4H), 7.35 – 7.26 (m, 1H) ppm.

^{13}C{^{1}H} NMR (101 MHz, $CDCl_3$): δ = 141.2, 138.6, 133.7, 132.7, 128.9, 128.5, 128.3, 127.7, 127.5, 127.4, 126.4, 126.0, 125.9, 125.7 ppm.

MS (EI): m/z (%) = 201.1 (100[M$^+$]), 202.0 (37), 203.1 (30), 205.1 (17), 101.0 (13).

Reference: J. L. Bolliger, C. M. Frech, *Adv. Synth. Catal.* **2010**, *352*, 1075 – 1080.

***O*-terphenyl** (**4ne**).

Phenyl potassium was prepared via Schlosser's base with benzene as the solvent. **4ne** was obtained with 24 % GC-conversion within a mixture of homocoupling products, via the general procedure for the direct cross-coupling **A** with 4-chloroanisole. The product can be identified in the spectrum of the mixture and the MS-data matches the expected product.

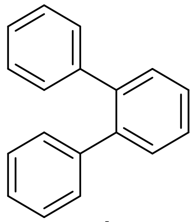

4ne

MS (EI): m/z (%) = 230.1(100 [M$^+$]), 229.1 (62), 215.0 (35), 228.1 (33), 231.1 (19), 226.0 (18), 202.0 (14), 114.0 (14), 113.0 (14), 227.1 (13), 101.0 (12).

Reference: T. Tu, Z. Sun, W. Fang, M. Xu, Y. Zhou, *Org. Lett.* **2012**, *14*, 4250 – 4253.

Cross coupling of terpenes:

While the cross-coupling of the terpene potassium compounds works reasonably well, the obtained final yields are considerably low. This is due to the low selectivity during the metalation, and therefore multiple possibilities for product formation during the cross couplings. The shown isolated compounds are the isomers which could be isolated relatively cleanly without any more complicated purification method. MS-data was obtained from the mixture of isomers. We recommend the usage of HPLC-technique in order to isolate all isomers and get 100% pure compounds.

(S)-2-(2-(4-methylcyclohex-3-en-1-yl)allyl)naphthalene (**4ob**).

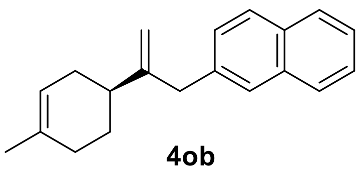

4ob

4ob was obtained with 25 % (65 mg, 1 mmol scale) yield via the general procedure for the direct cross-coupling **A** with 2-chloronaphthalene. The sample was purified via column chromatography (hexane/EtOAc 100:0).

^{1}H-NMR (300 MHz, $CDCl_3$): δ = 7.83 – 7.72 (m, 3H), 7.64 – 7.58 (m, 1H), 7.51 – 7.37 (m, 2H), 7.32 (dd, *J* = 8.4, 1.8 Hz, 1H), 5.40 – 5.32 (m, 1H), 4.91 (s, 1H), 4.79 – 4.67 (m, 1H), 3.55 (s, 2H), 2.08 (d, *J* = 11.5 Hz, 3H), 2.00 – 1.88 (m, 3H), 1.83 (ddq, *J* = 12.5, 4.7, 2.4 Hz, 2H), 1.61 (t, *J* = 1.4 Hz, 3H) ppm.

^{13}C NMR (75 MHz, $CDCl_3$): δ = 153.6, 137.7, 133.9, 133.7, 132.2, 127.9, 127.9, 127.7, 127.6, 127.4, 125.9, 125.3, 120.7, 110.3, 42.3, 39.0, 31.5, 30.7, 28.4, 23.5 ppm.

MS (EI): m/z (%) = 179.0 (100), 262.1(94 [M$^+$]), 141.0 (92), 165.0 (61), 121.1 (55), 115.0 (48), 178.0 (45), 167.1 (41), 93.0 (40), 193.1 (32), 194.1 (30), 166.0 (29), 142.0 (24), 79.0 (22), 91.0 (20), 263.2 (20), 128.0 (18), 119.0 (18), 180.1 (18), 168.1 (18), 129.0 (16).

HRMS-QTOF (m/z) = [$C_{20}H_{22}$] calcd for $C_{20}H_{22}$, 262.1722; found, 262.1726.

(1R,5S)-6,6-dimethyl-2-(naphthalen-2-ylmethyl)bicyclo[3.1.1]hept-2-ene (**4pb**).

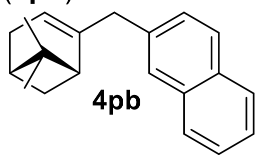

4pb

4pb was obtained with 27 % (138 mg, 2 mmol scale) yield with minor impurities of differet isomers via the general procedure for the direct cross-coupling **A** with 2-chloronaphthalene. The sample was purified via column chromatography (hexane/EtOAc 100:0).

^{1}H-NMR (300 MHz, $CDCl_3$): δ = 7.86 – 7.63 (m, 4H), 7.53 – 7.29 (m, 4H), 6.19 (d, J = 2.3 Hz, 1H), 2.79 (ddt, J = 8.1, 6.4, 2.5 Hz, 2H), 2.59 (t, J = 5.4 Hz, 1H), 2.44 (dtd, J = 9.9, 5.8, 1.5 Hz, 1H), 2.09 – 1.98 (m, 1H), 1.98 – 1.78 (m, 1H), 1.53 (d, J = 9.8 Hz, 1H), 1.29 (s, 3H), 0.76 (s, 4H) ppm.
^{13}C{^{1}H} NMR (75 MHz, $CDCl_3$): δ = 147.5, 136.2, 133.6, 131.7, 127.9, 127.8, 127.7, 127.6, 127.6, 127.5, 127.4, 126.9, 126.0, 125.3, 122.2, 54.0, 41.4, 40.8, 27.7, 26.4, 26.3, 24.2, 22.2, 22.2 ppm.
MS (EI): m/z (%) = 141.0 (100), 91.0 (30), 218.1 (21), 262.2 (21 [M$^+$]), 115.0 (17), 142.1 (16), 121.1 (13), 93.0 (12), 178.1 (11).

HRMS-QTOF (m/z) = [$C_{20}H_{22}$] calcd for $C_{20}H_{22}$, 262.1722; found, 262.1724.

2.2. NMR spectra of the isolated compounds

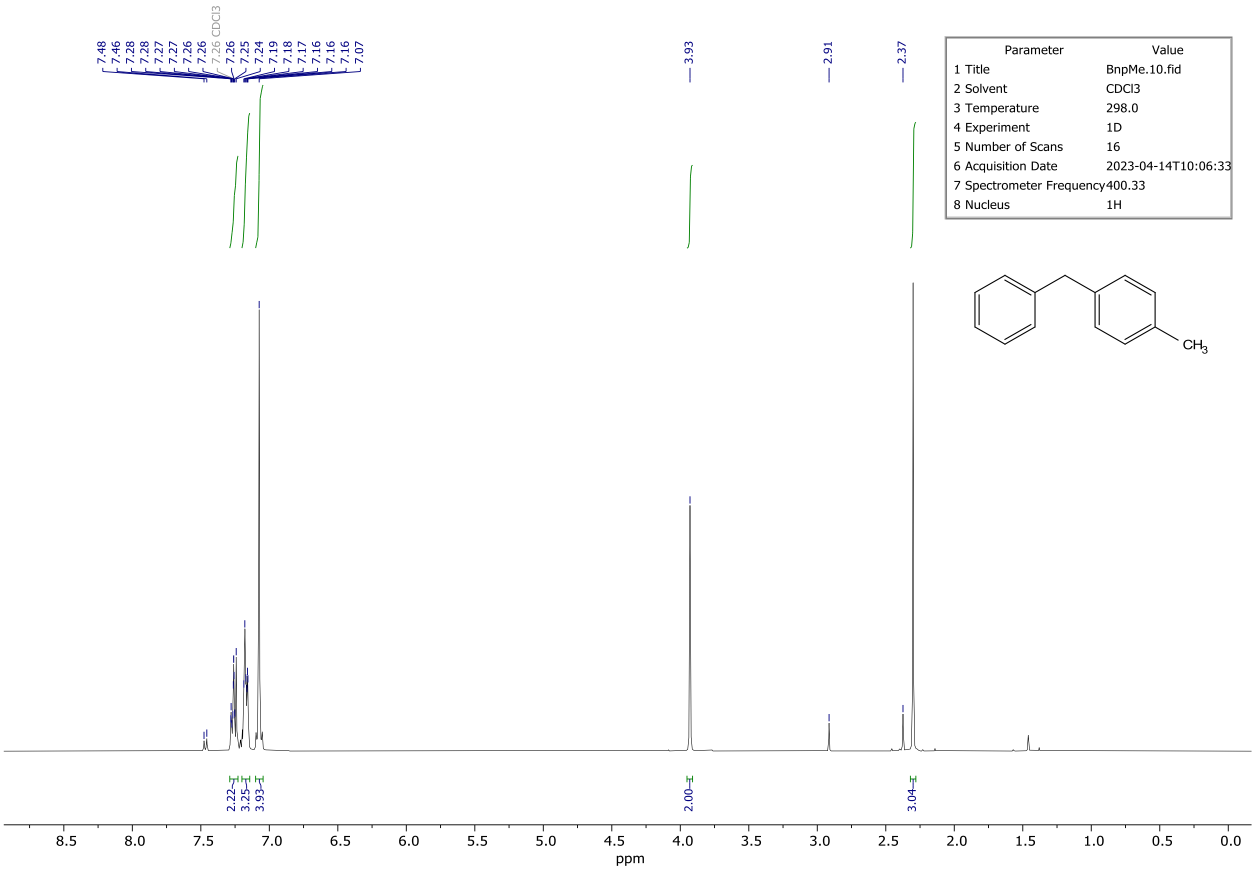

Figure S 15. ^{1}H-NMR spectrum of **4ac**. Impurities of homocoupling products are visible as minor impurities.

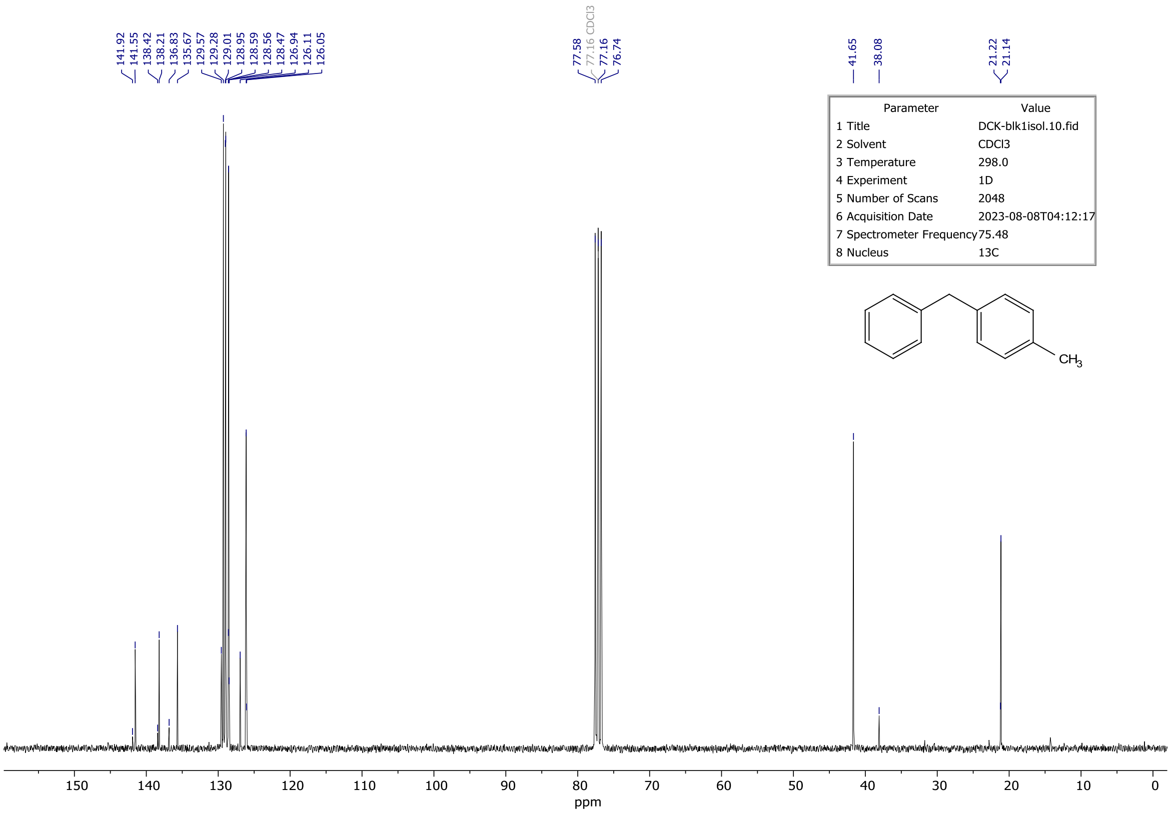

Figure S 16. $^{13}C\{^{1}H\}$-NMR spectrum of **4ac** as a mixture with homocoupling products as minor impurities.

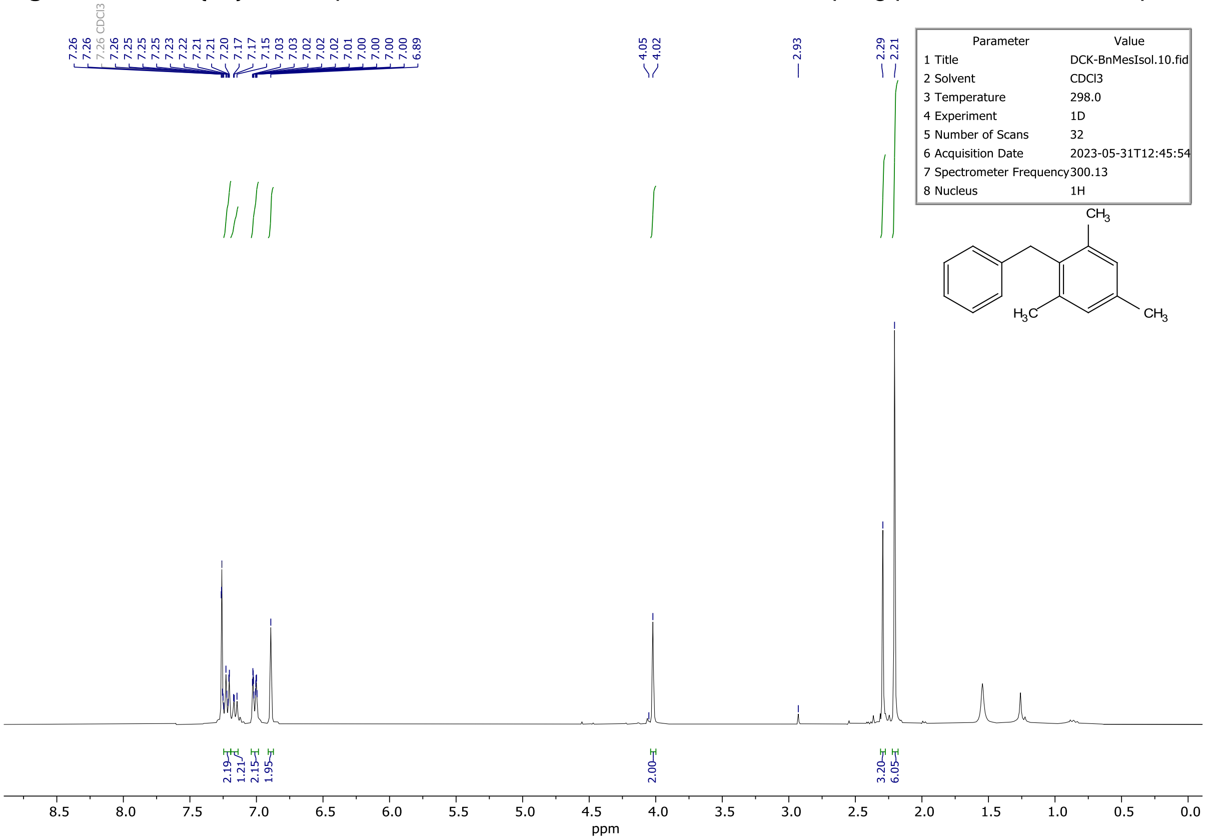

Figure S 17. ^{1}H-NMR spectrum of **4ai** with homocoupling product of BzK as minor impurity.

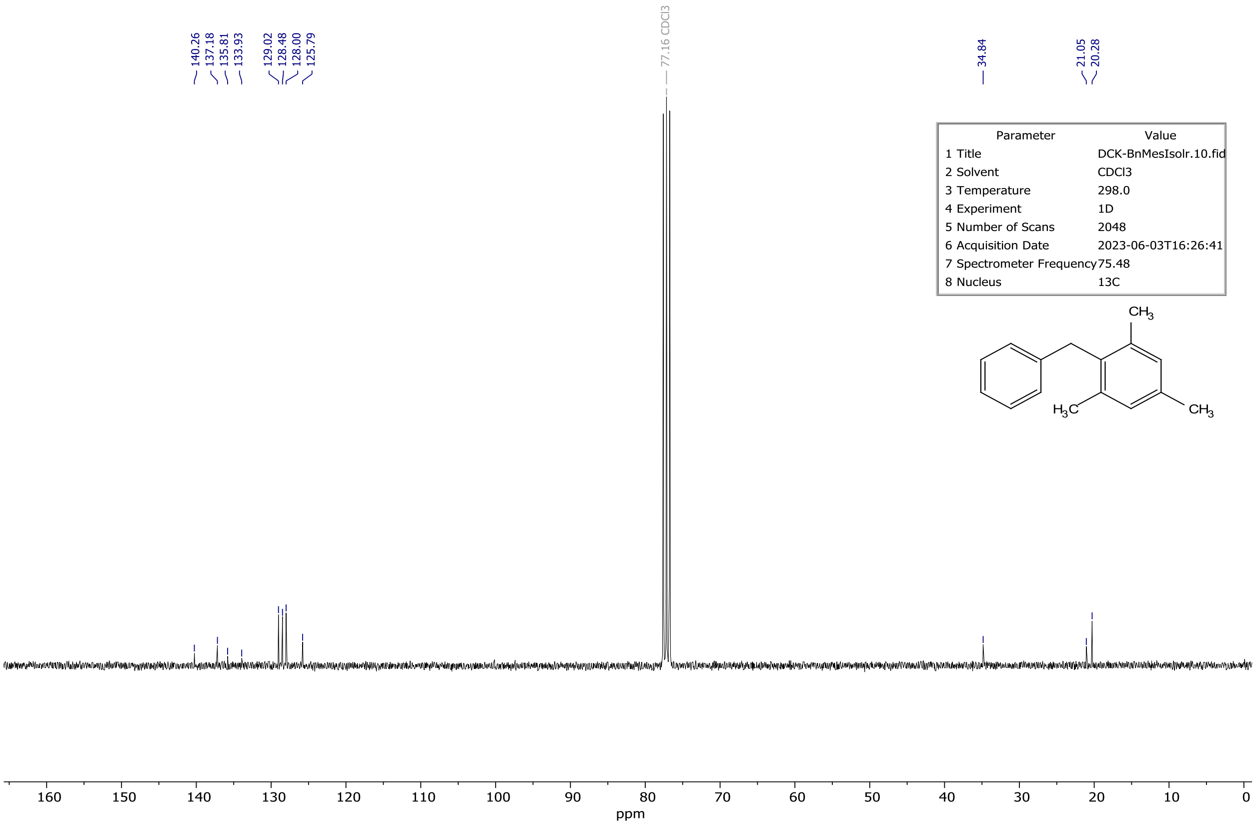

Figure S 18. $^{13}C\{^{1}H\}$-NMR spectrum of **4ai** with homocoupling product of BzK as minor impurity.

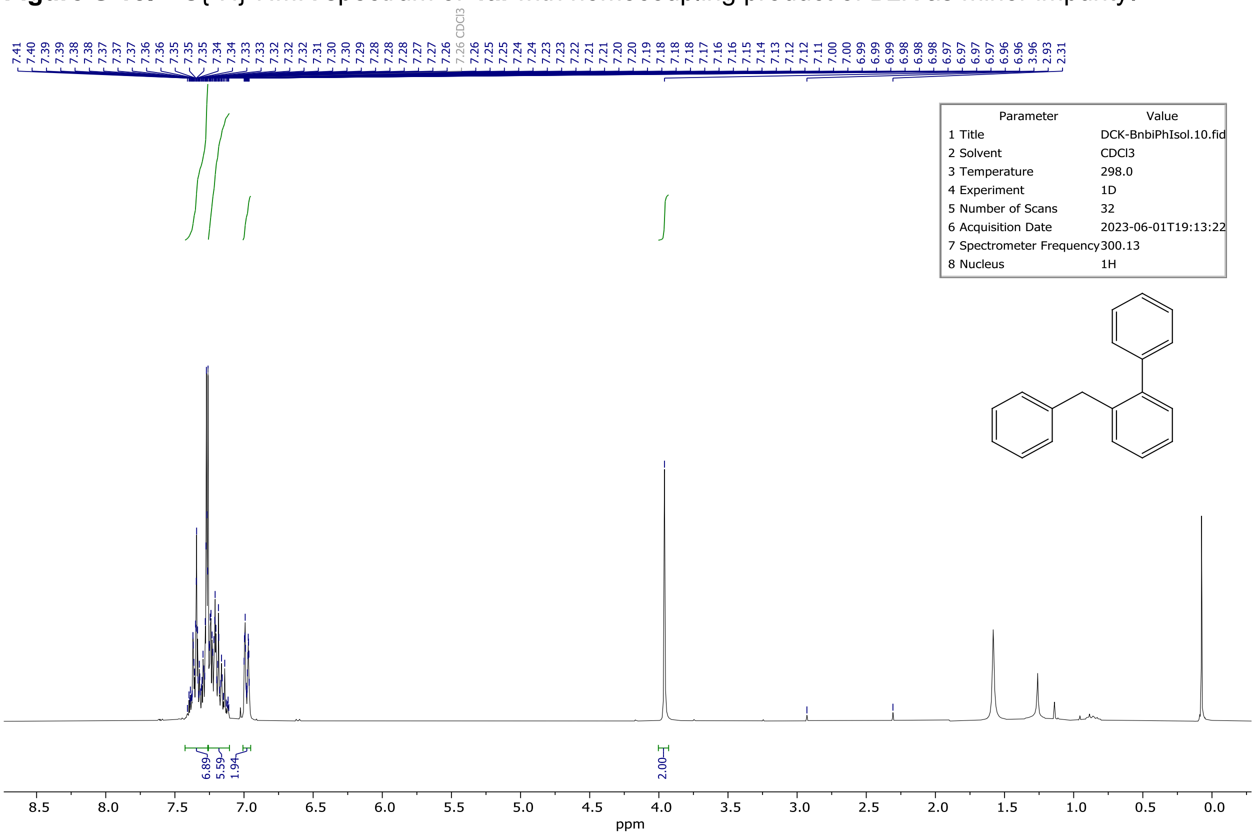

Figure S 19. ^{1}H-NMR spectrum of **4ae** with homocoupling product of BzK as minor impurity.

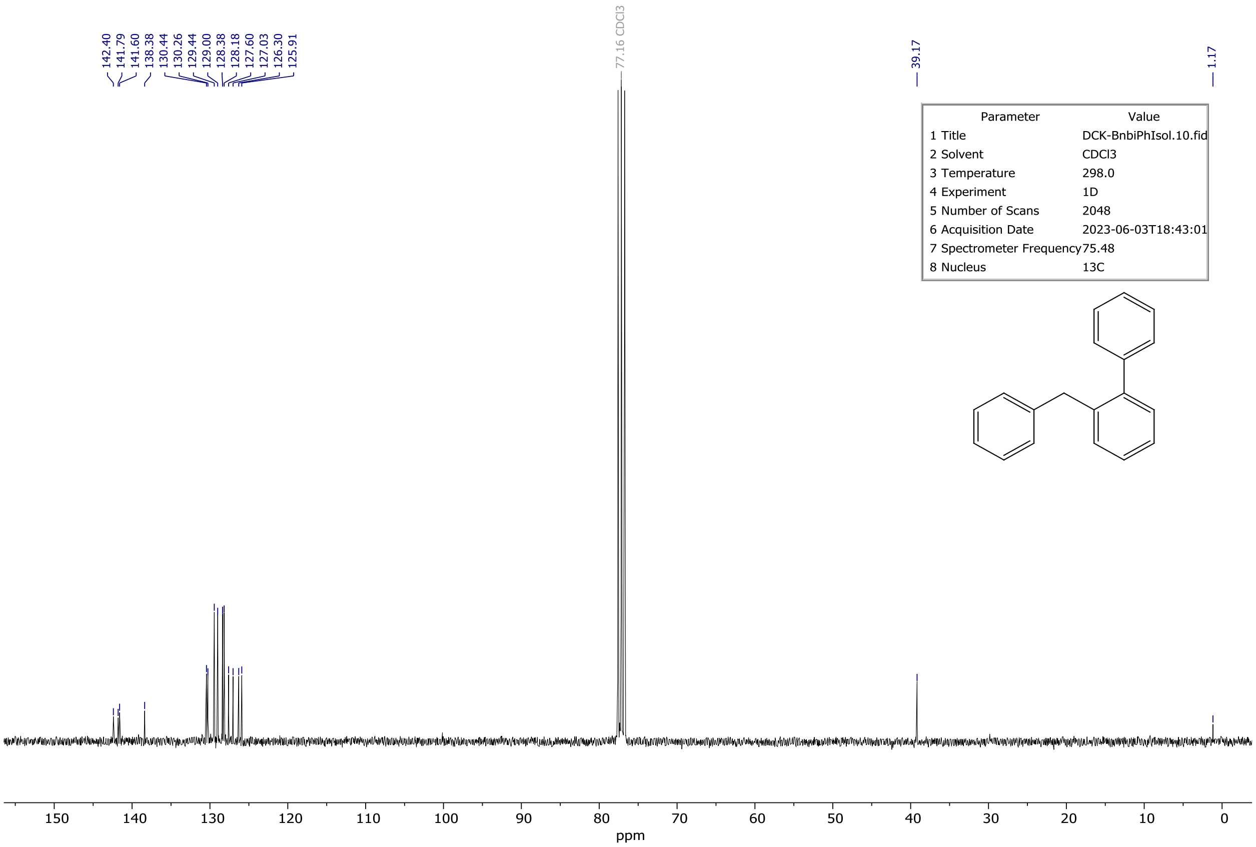

Figure S 20. $^{13}C\{^{1}H\}$-NMR spectrum of **4ae** with homocoupling product of BzK as minor impurity.

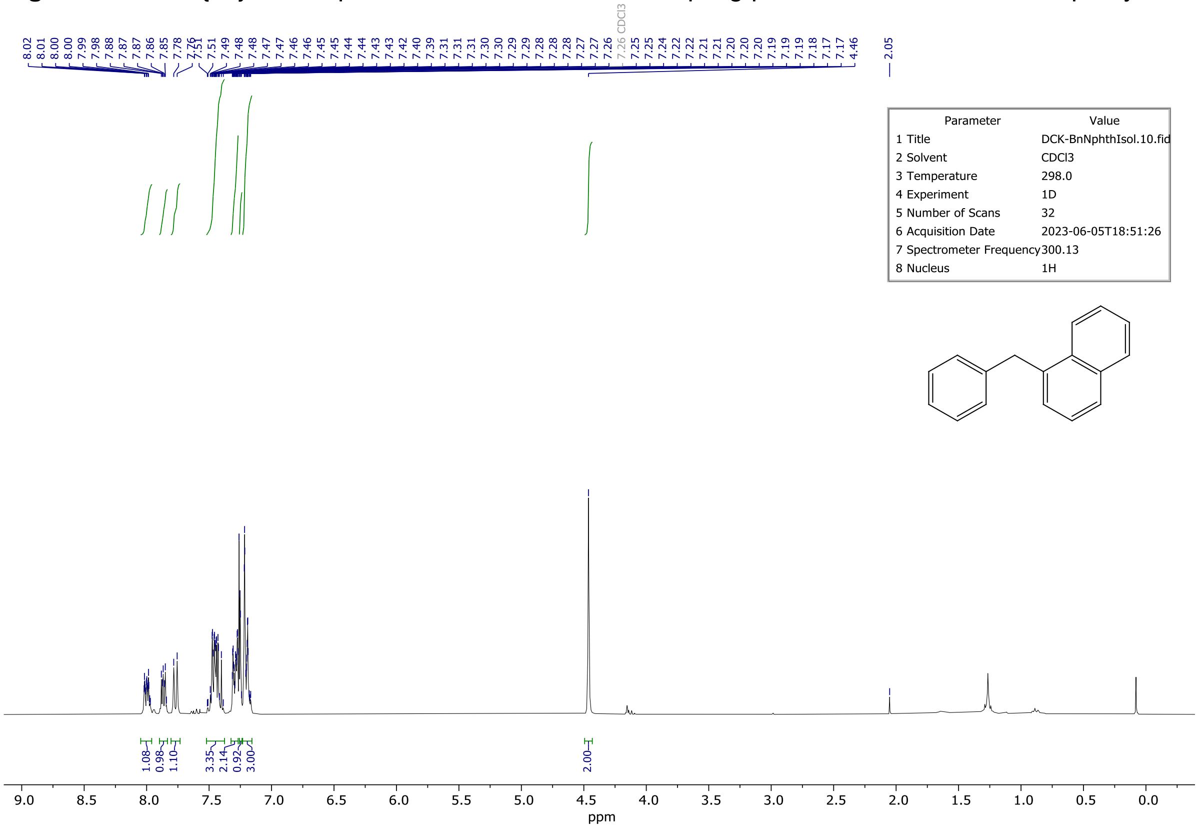

Figure S 21. ^{1}H-NMR spectrum of **4ah**.

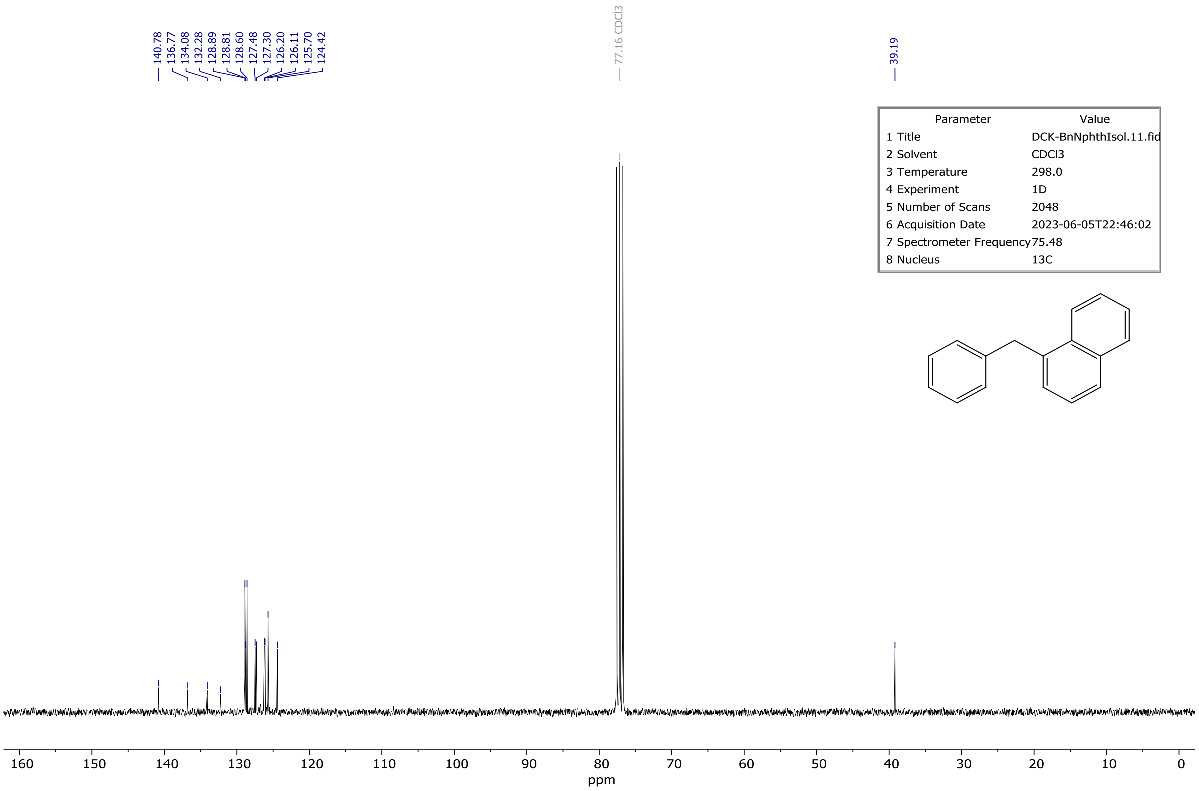

Figure S 22. $^{13}C\{^{1}H\}$-NMR spectrum of **4ah**.

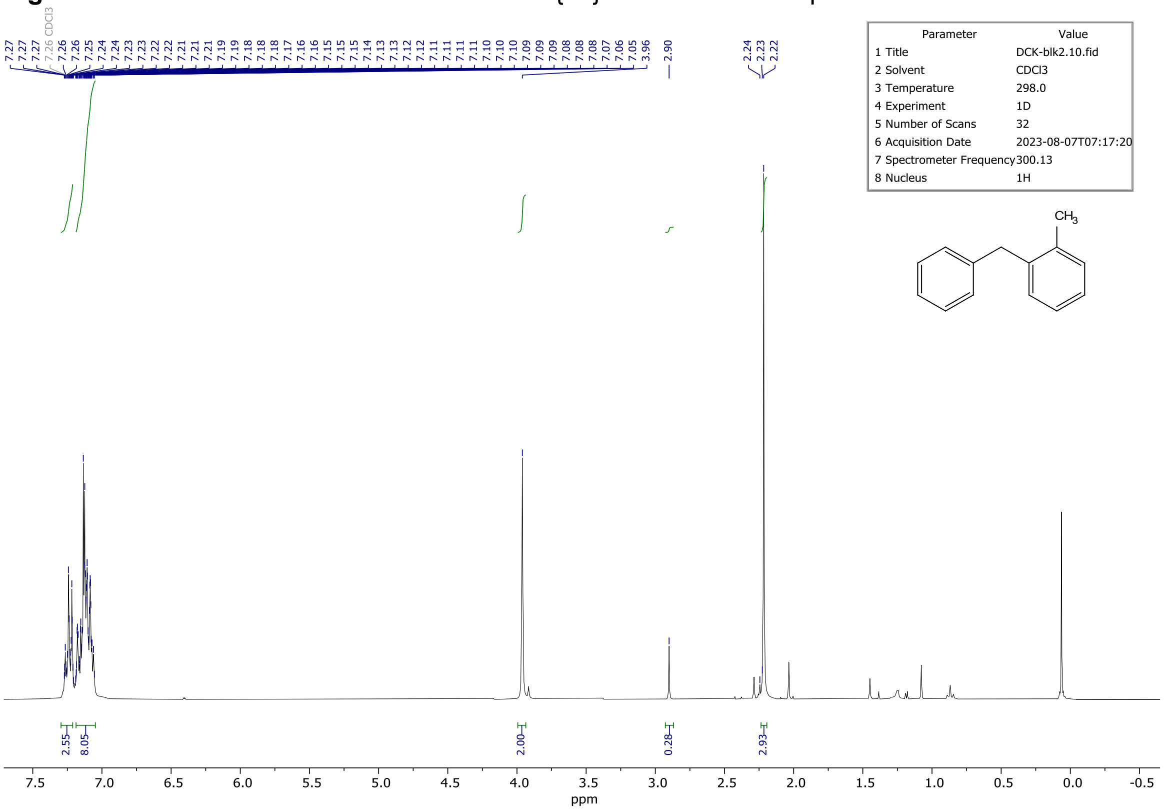

Figure S 23. ^{1}H-NMR spectrum of **4aj** as a mixture with homocoupling products. Residual grease at 0.07 ppm

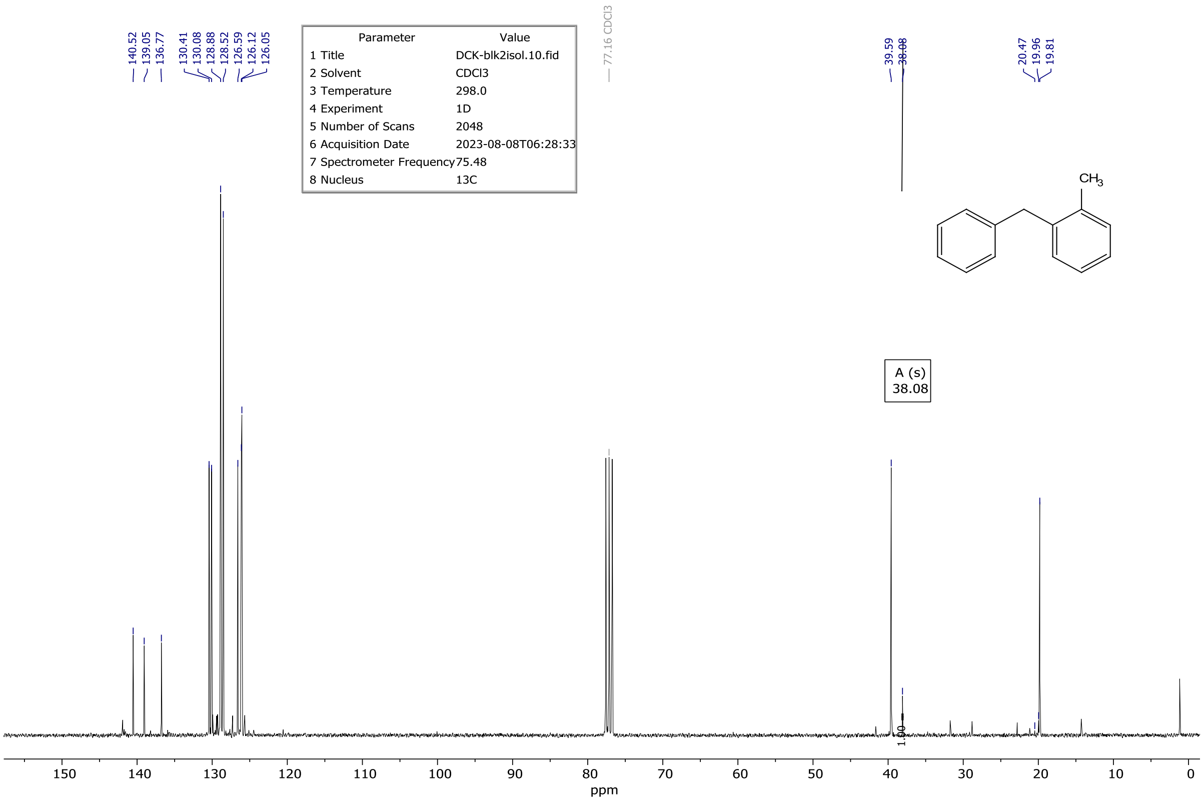

Figure S 24. $^{13}C\{^{1}H\}$-NMR spectrum of **4aj** as a mixture with homocoupling products. Residual grease at 1.18 ppm.

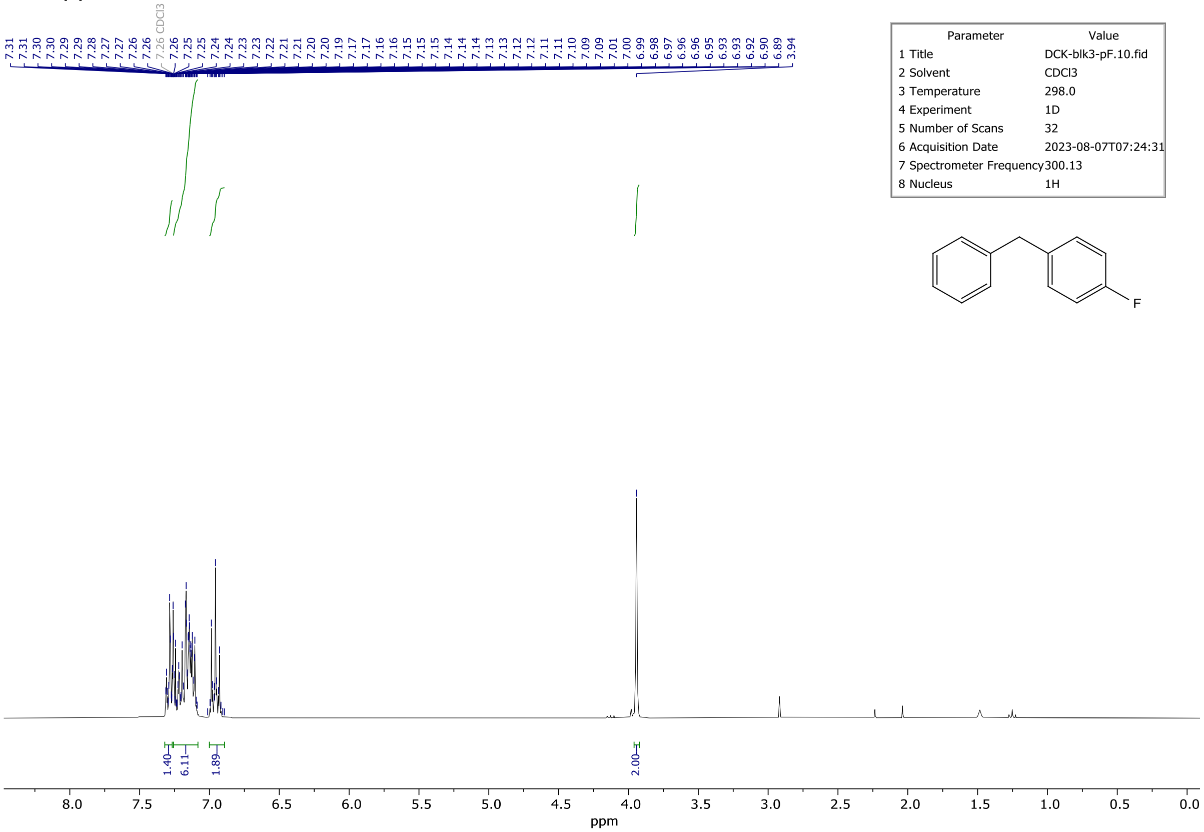

Figure S 25. ^{1}H-NMR spectrum of **4af** with homocoupling product of BzK as minor impurity.

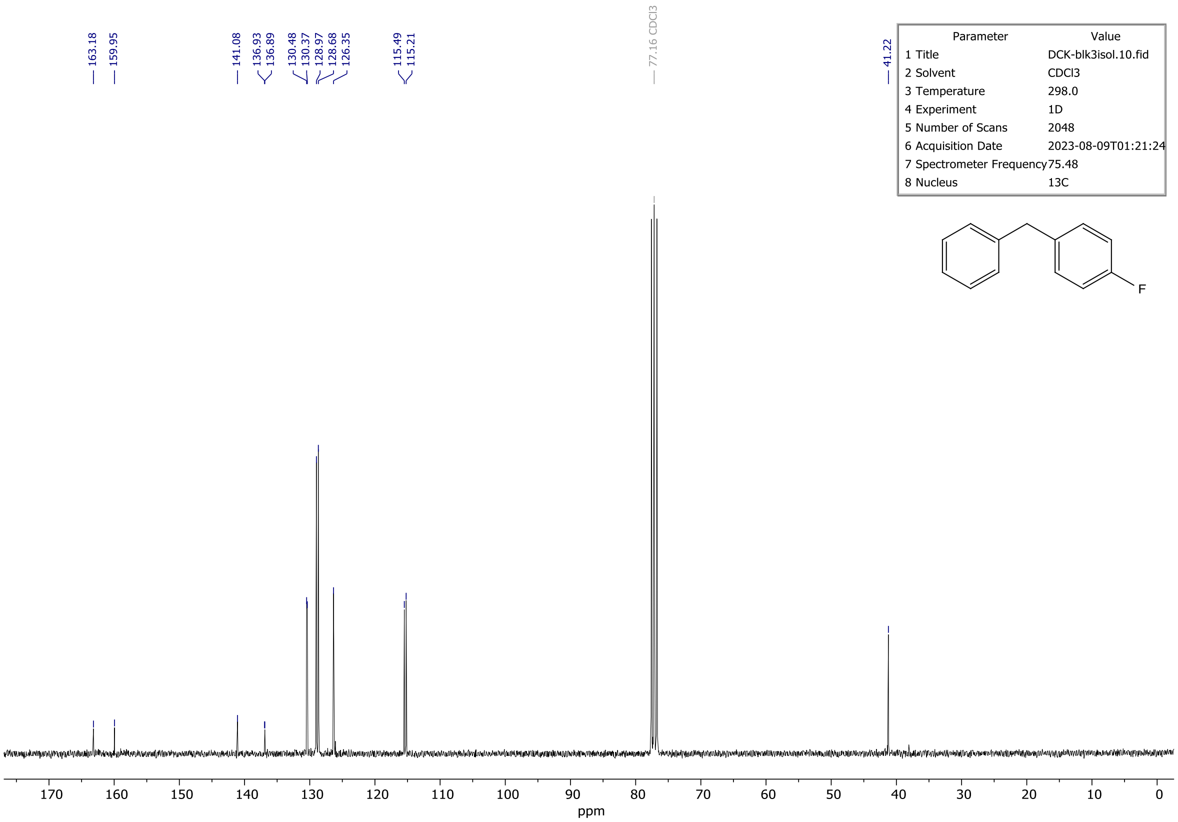

Figure S 26. $^{13}C\{^1H\}$-NMR spectrum of **4af** with homocoupling product of BzK as minor impurity.

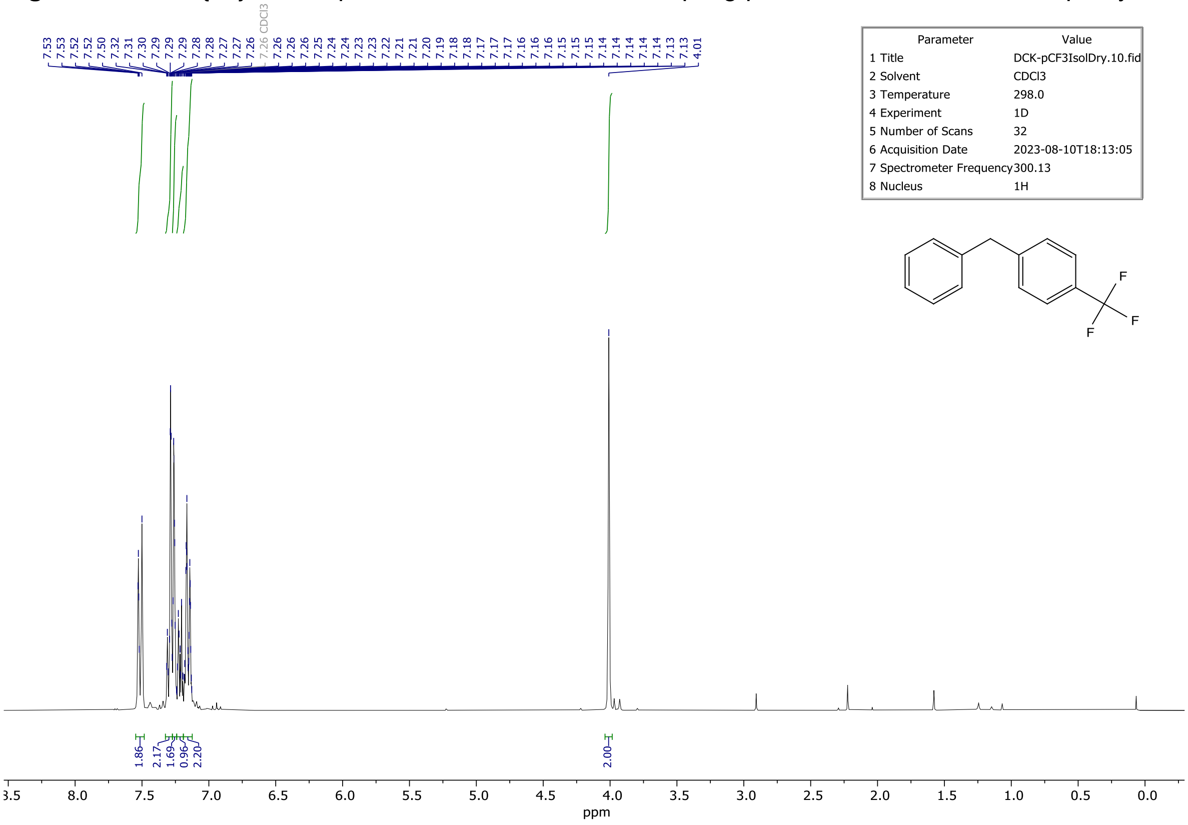

Figure S 27. ^1H-NMR spectrum of **4ag.**

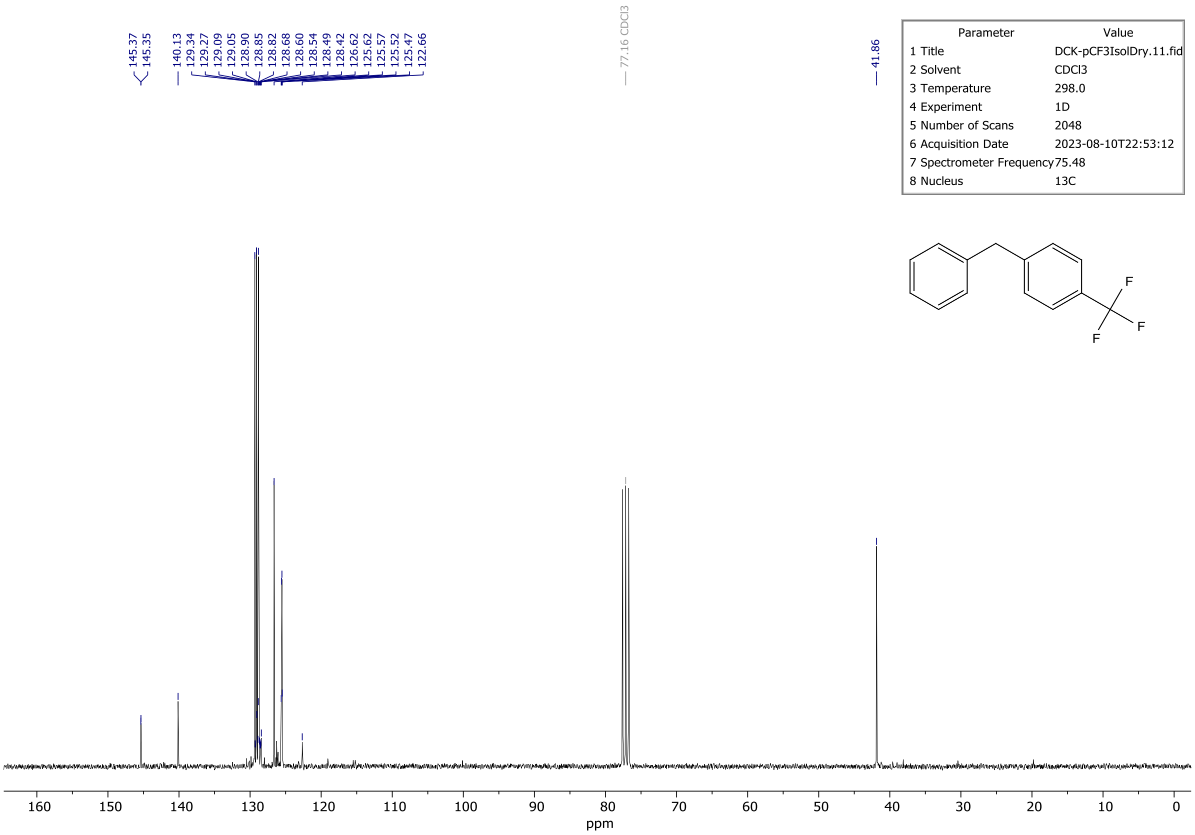

Figure S 28. $^{13}C\{^{1}H\}$-NMR spectrum of **4ag**.

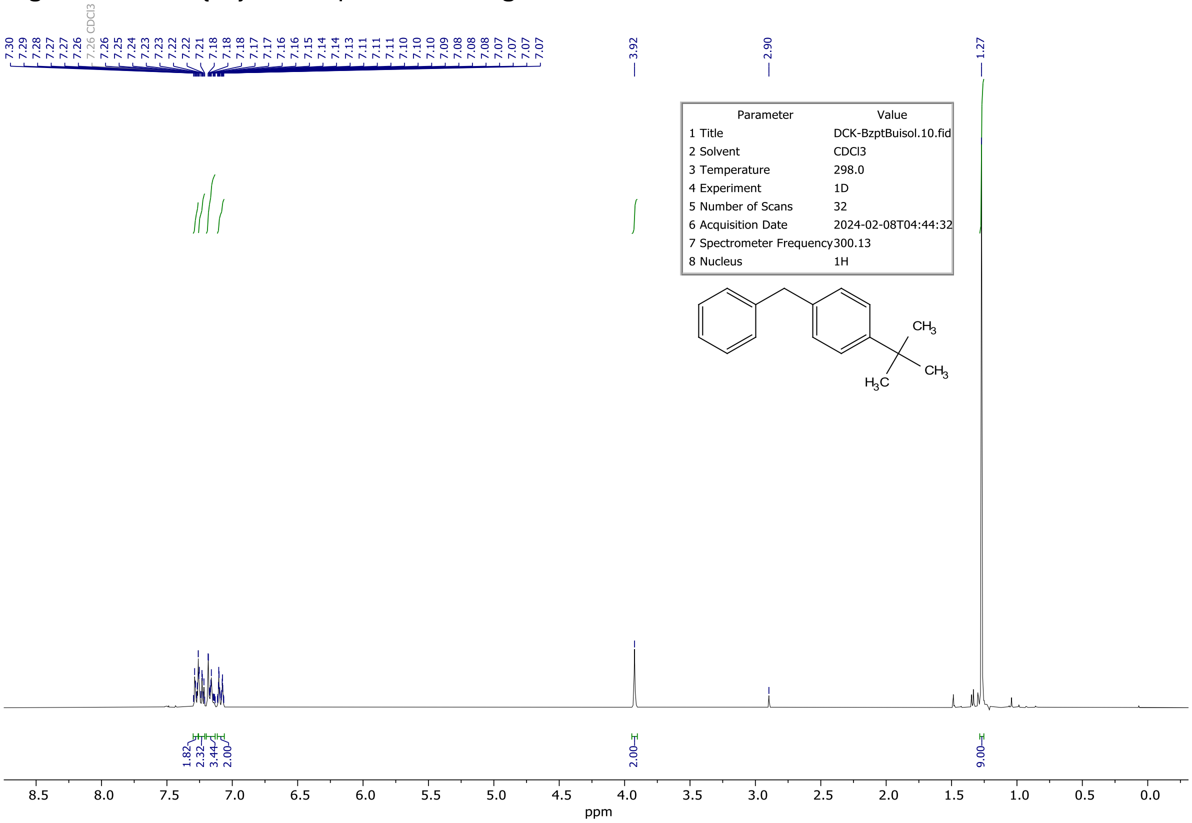

Figure S 29. ^{1}H-NMR spectrum of **4ad** with homocoupling product of BzK as minor impurity.

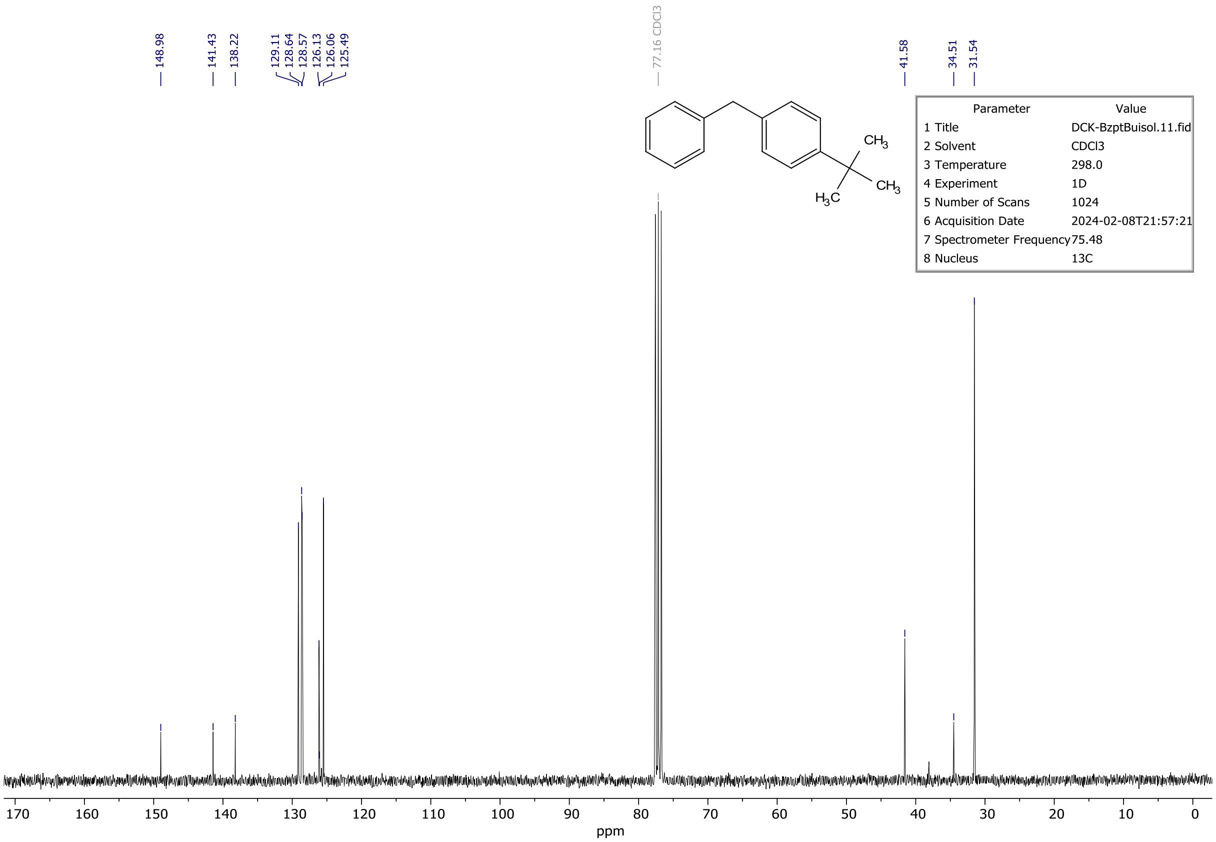

Figure S 30. $^{13}C\{^{1}H\}$-NMR spectrum of **4ad** with homocoupling product of BzK as minor impurity.

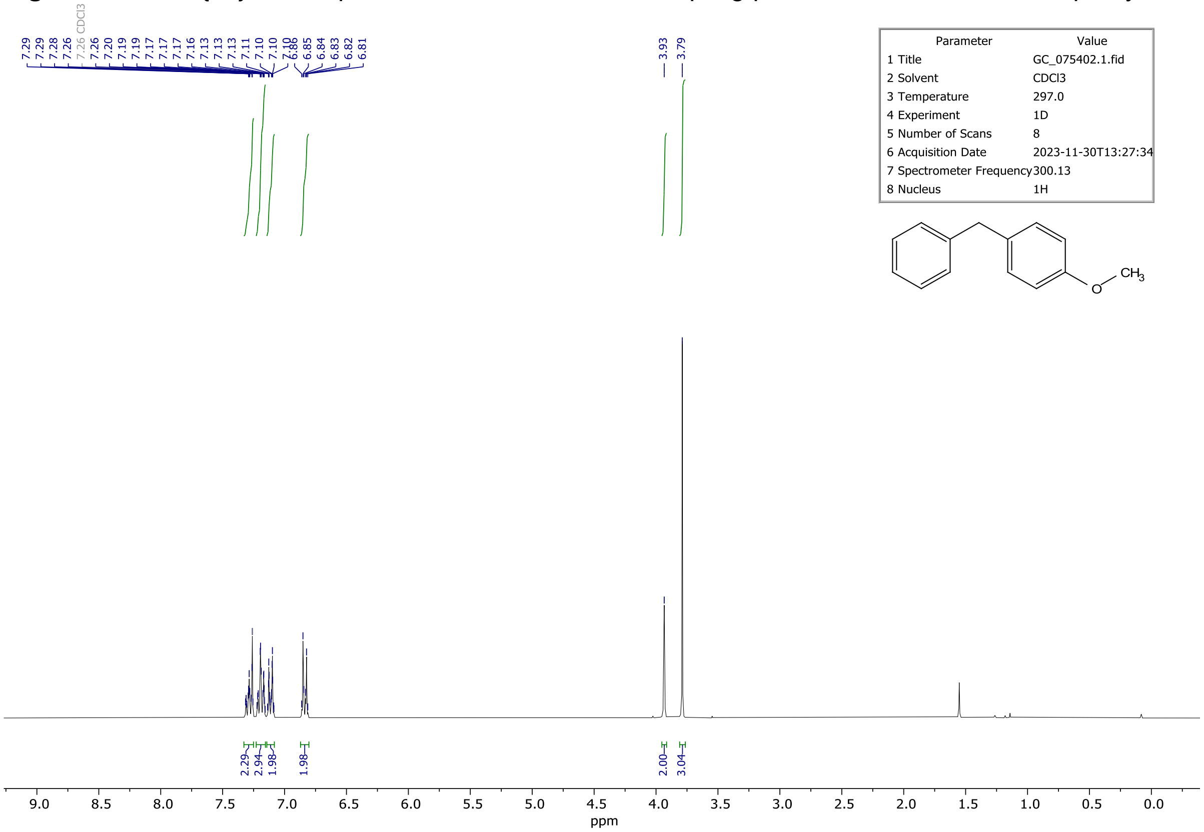

Figure S 31. ^{1}H-NMR spectrum of **4aa**.

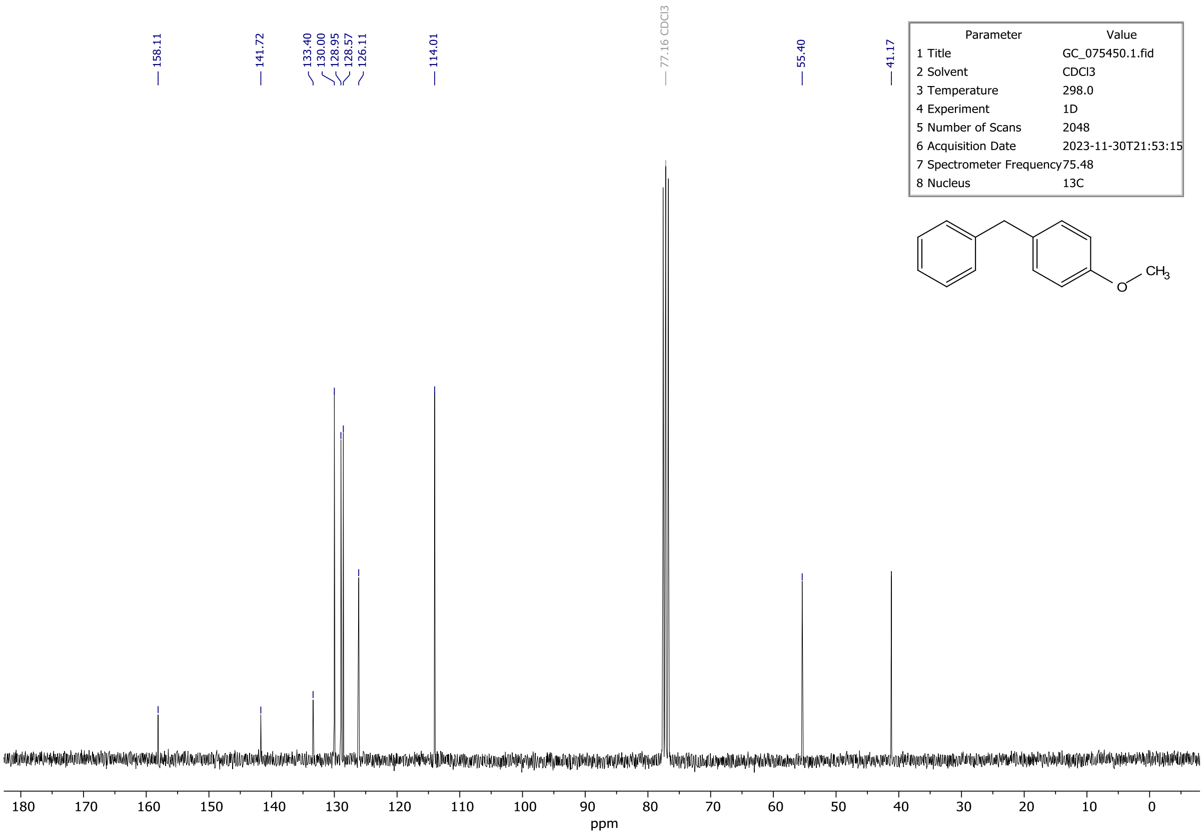

Figure S 32. $^{13}C\{^{1}H\}$-NMR spectrum of **4aa**.

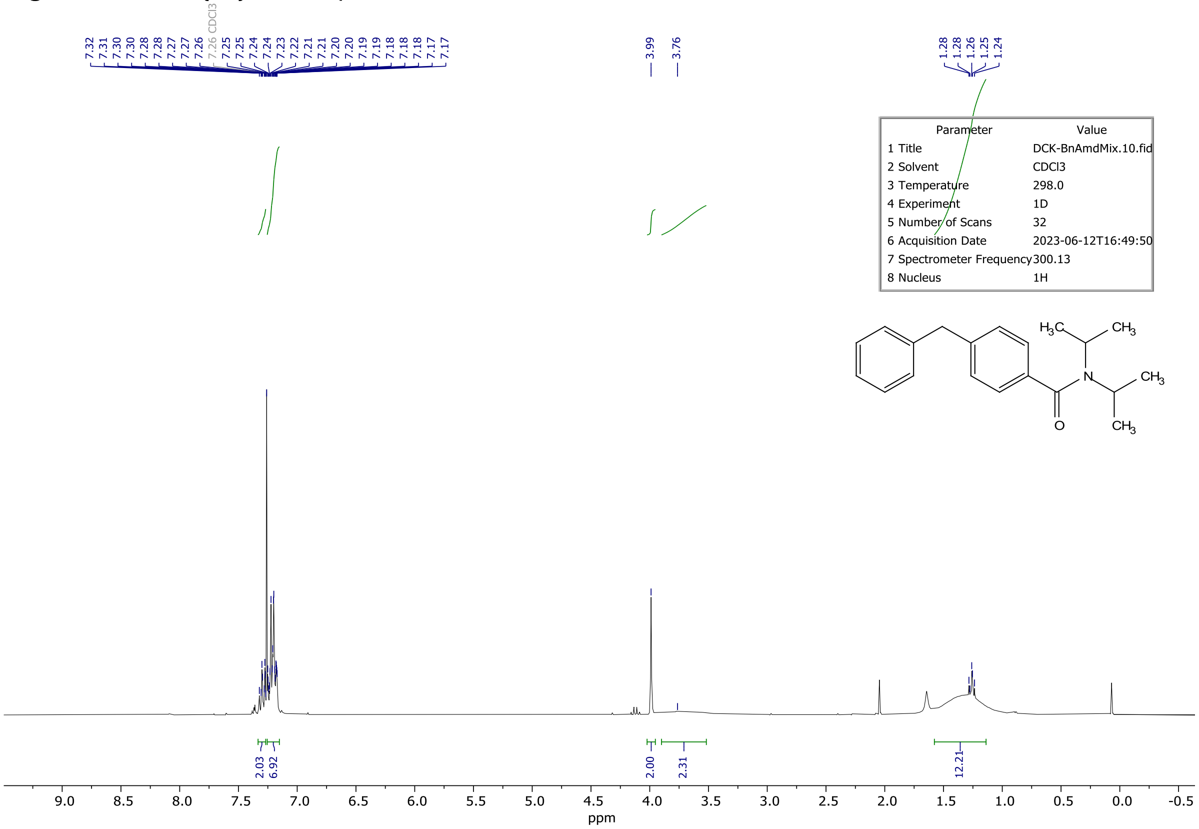

Figure S 33. ^{1}H-NMR spectrum of **4al** with residual EtOAc.

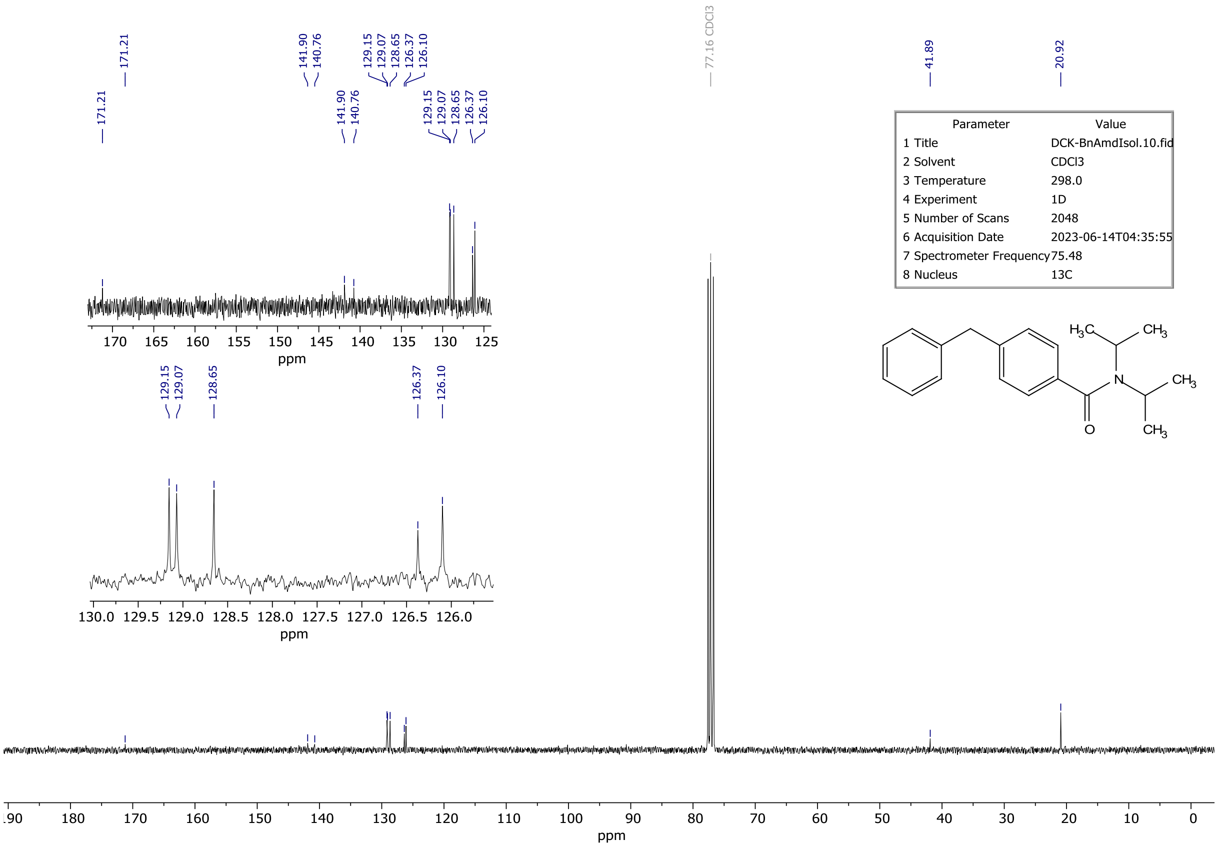

Figure S 34. $^{13}C\{^{1}H\}$-NMR spectrum of **4al**.

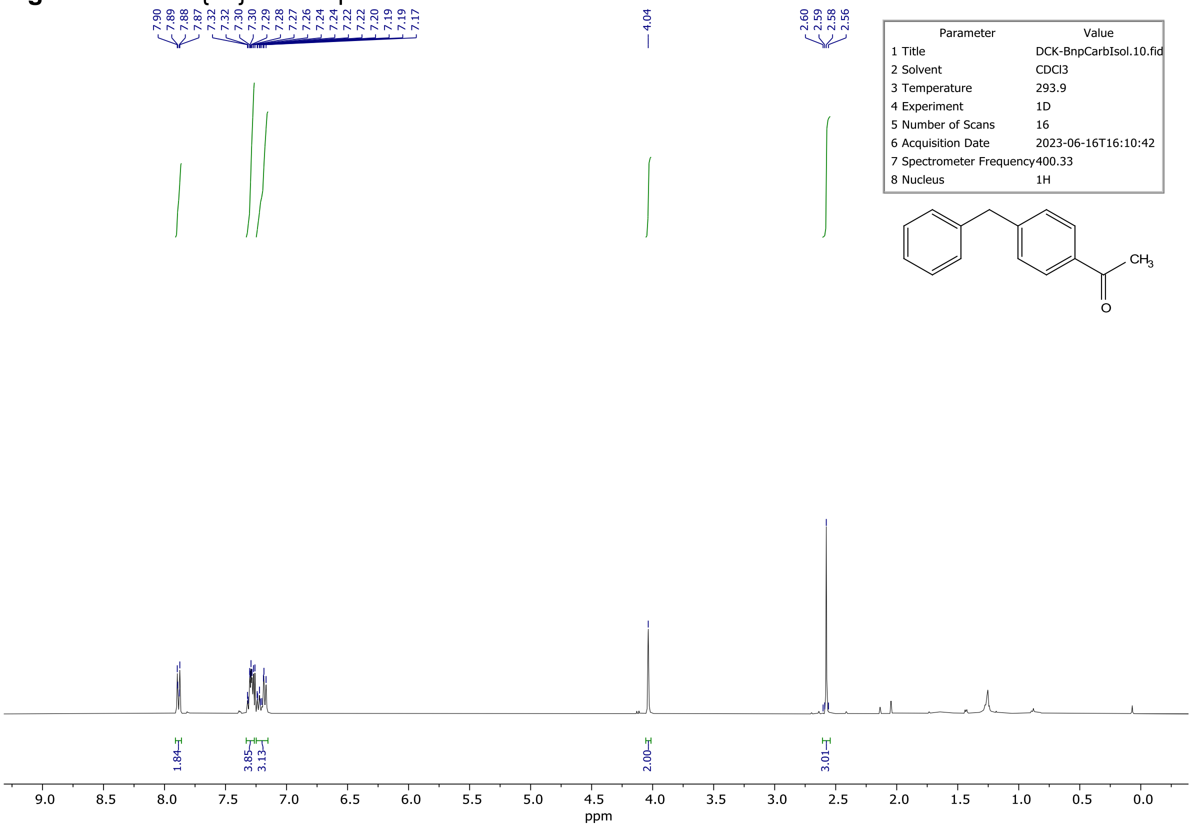

Figure S 35. ^{1}H-NMR spectrum of **4ak**.

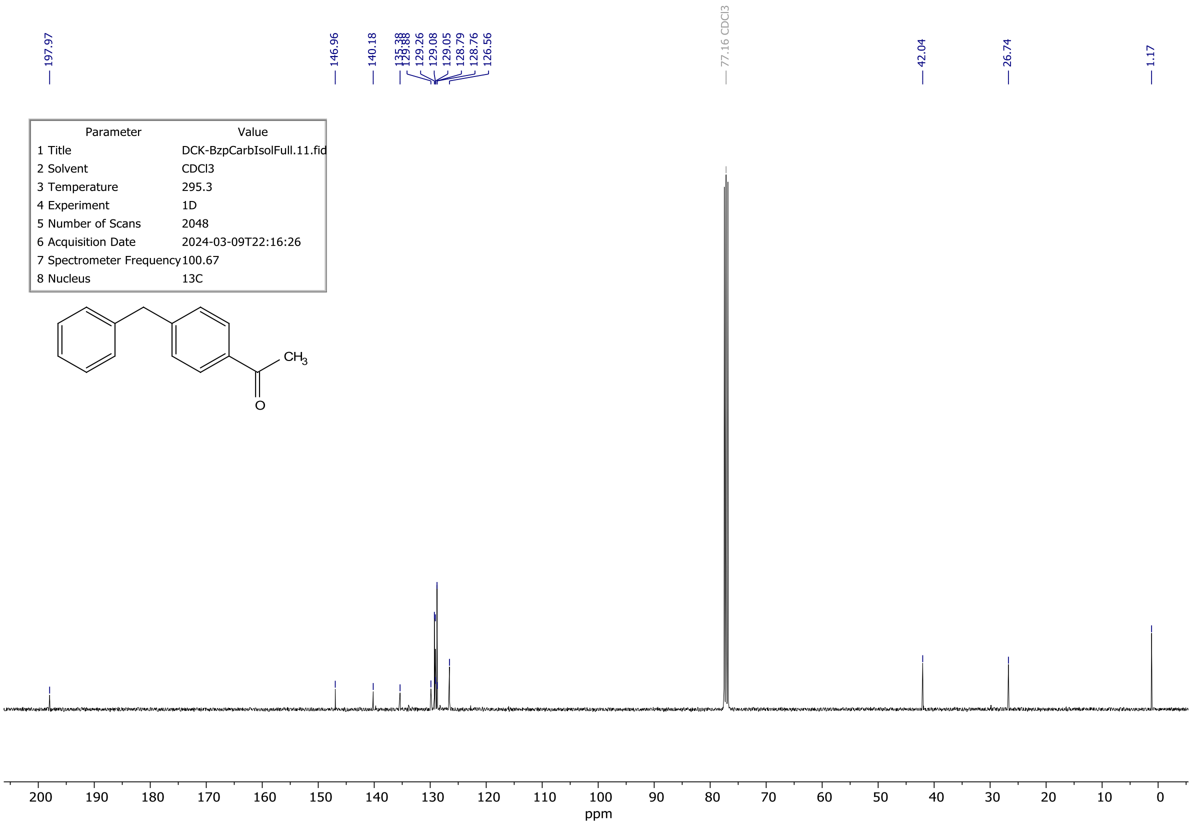

Figure S 36. $^{13}C\{^1H\}$-NMR spectrum of **4ak**.

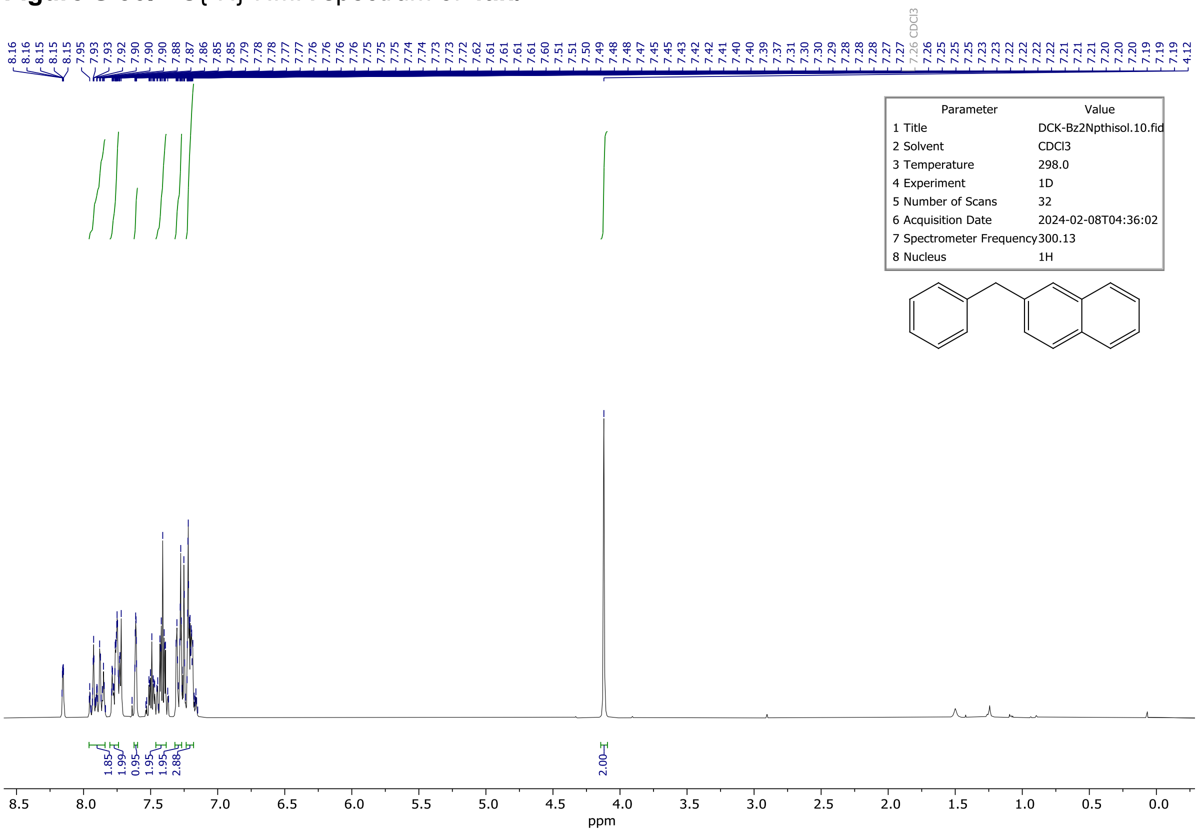

Figure S 37. ^1H-NMR spectrum of **4ab**.

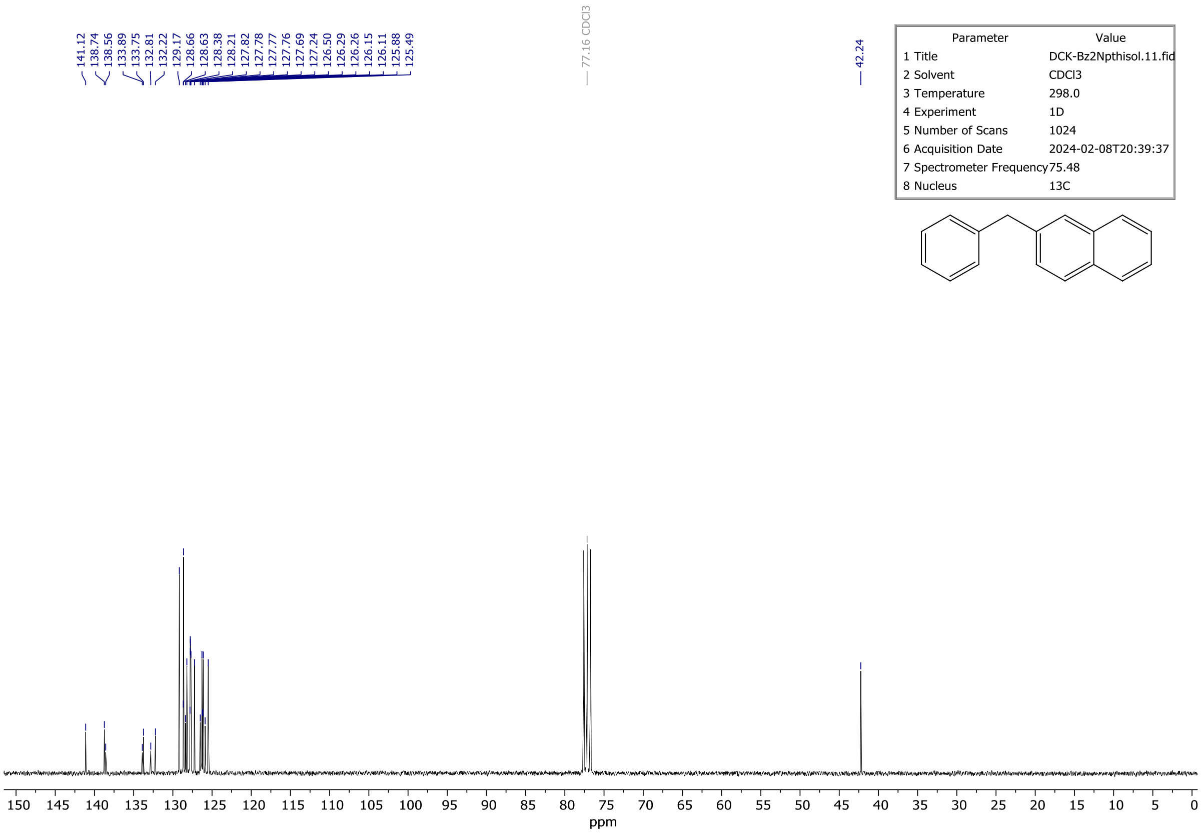

Figure S 38. $^{13}C\{^{1}H\}$-NMR spectrum of **4ab**.

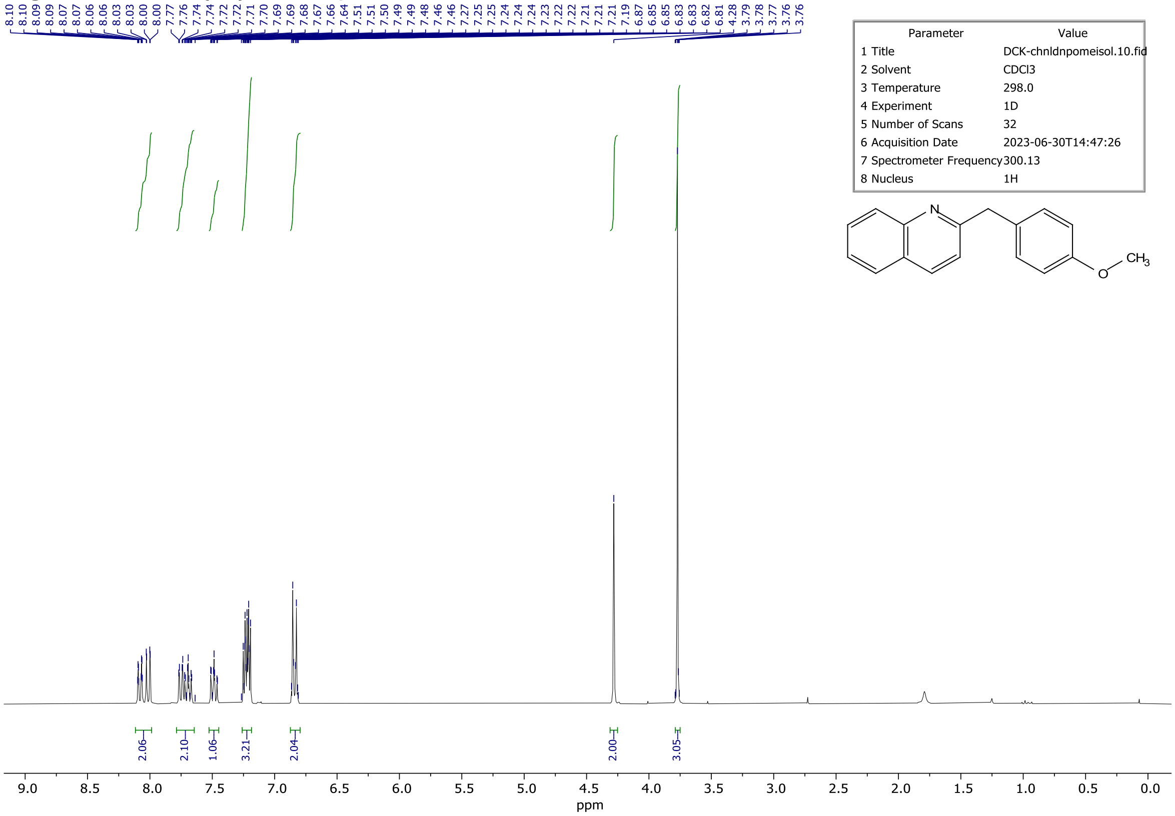

Figure S 39. ^{1}H-NMR spectrum of **4ba**.

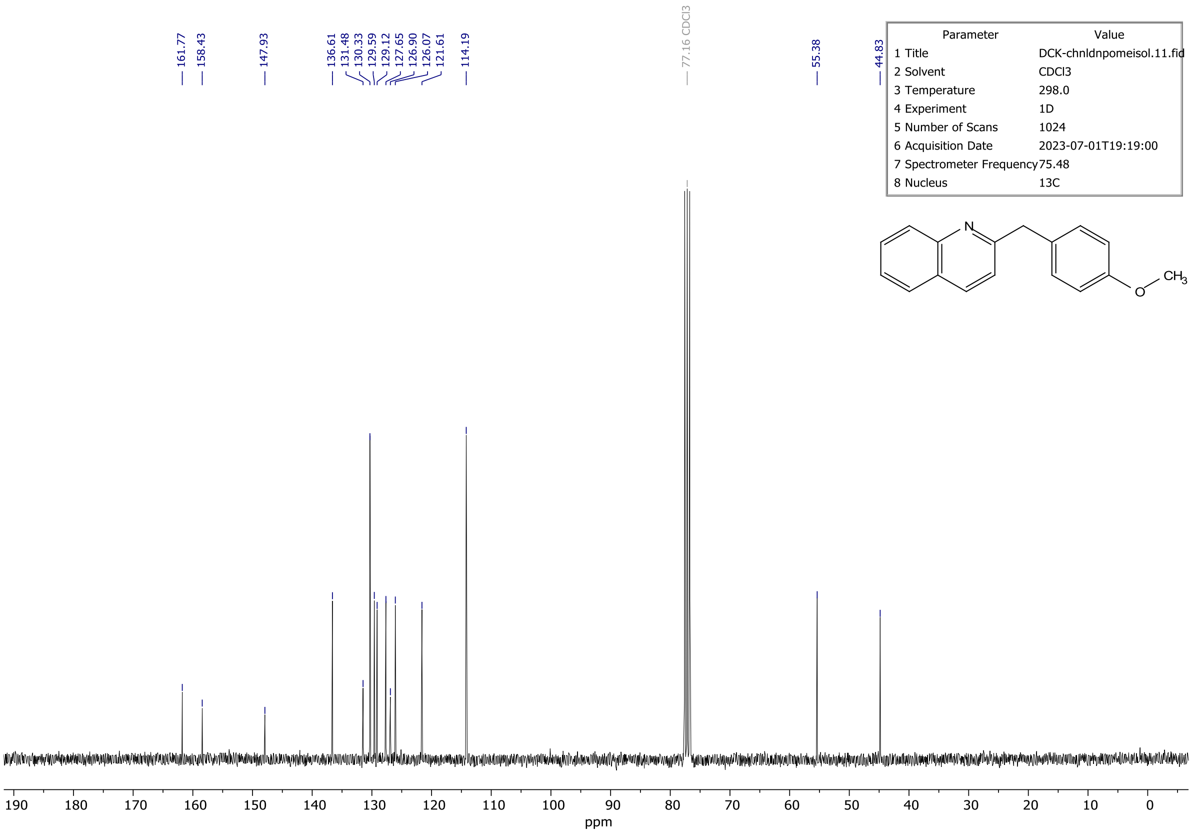

Figure S 40. $^{13}C\{^{1}H\}$-NMR spectrum of **4ba**.

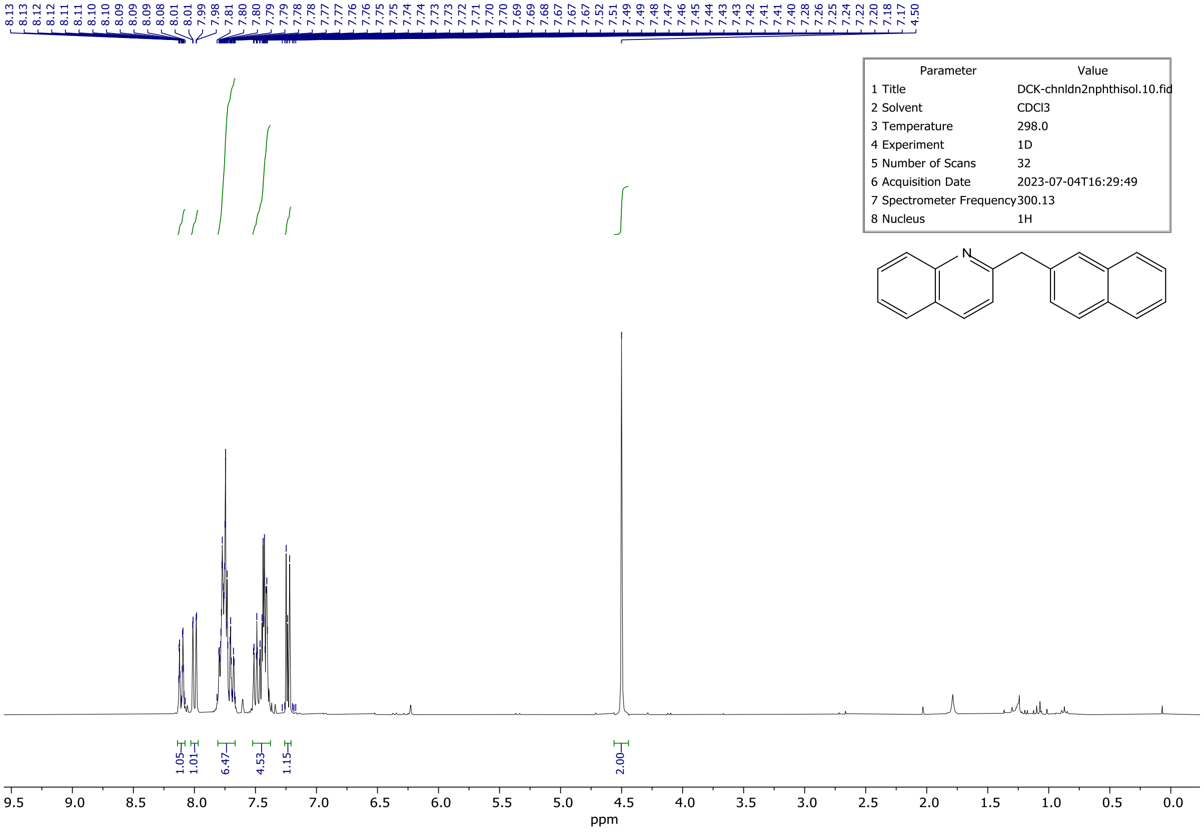

Figure S 41. ^{1}H-NMR spectrum of **4bb**.

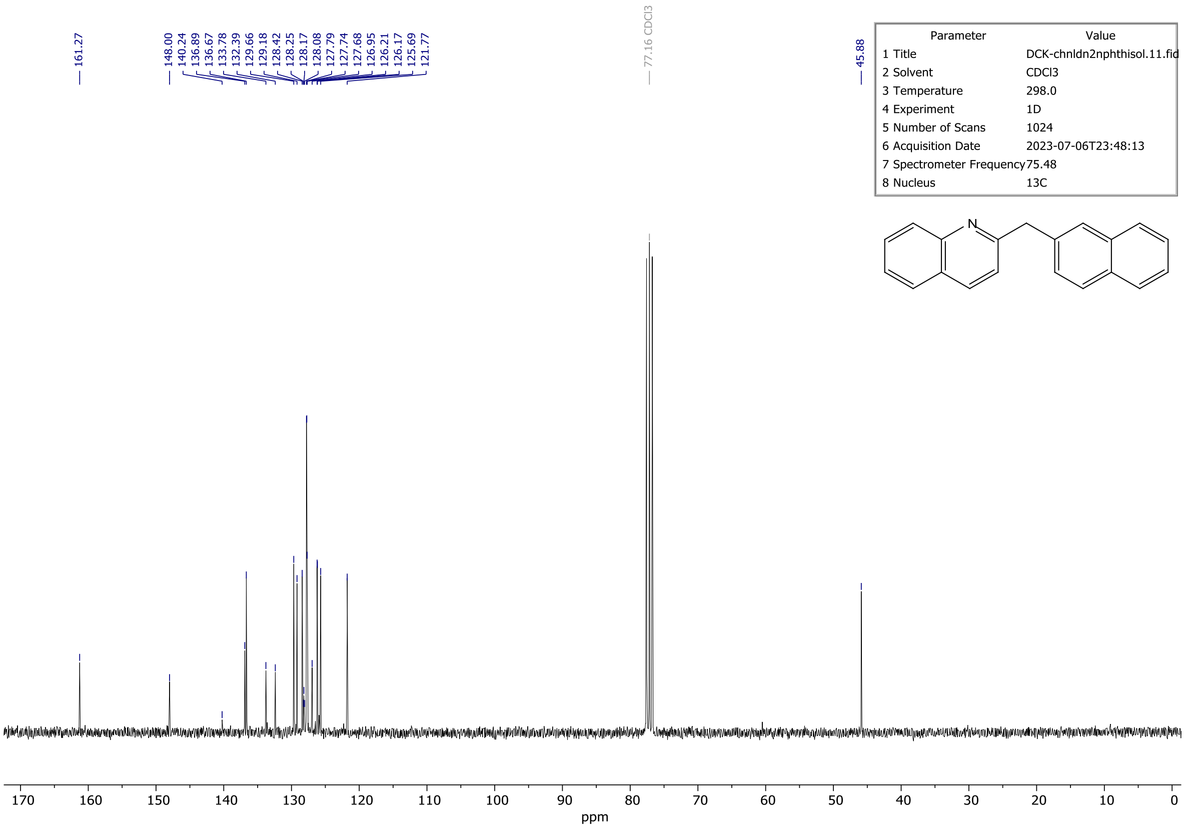

Figure S 42. $^{13}C\{^{1}H\}$-NMR spectrum of **4bb**.

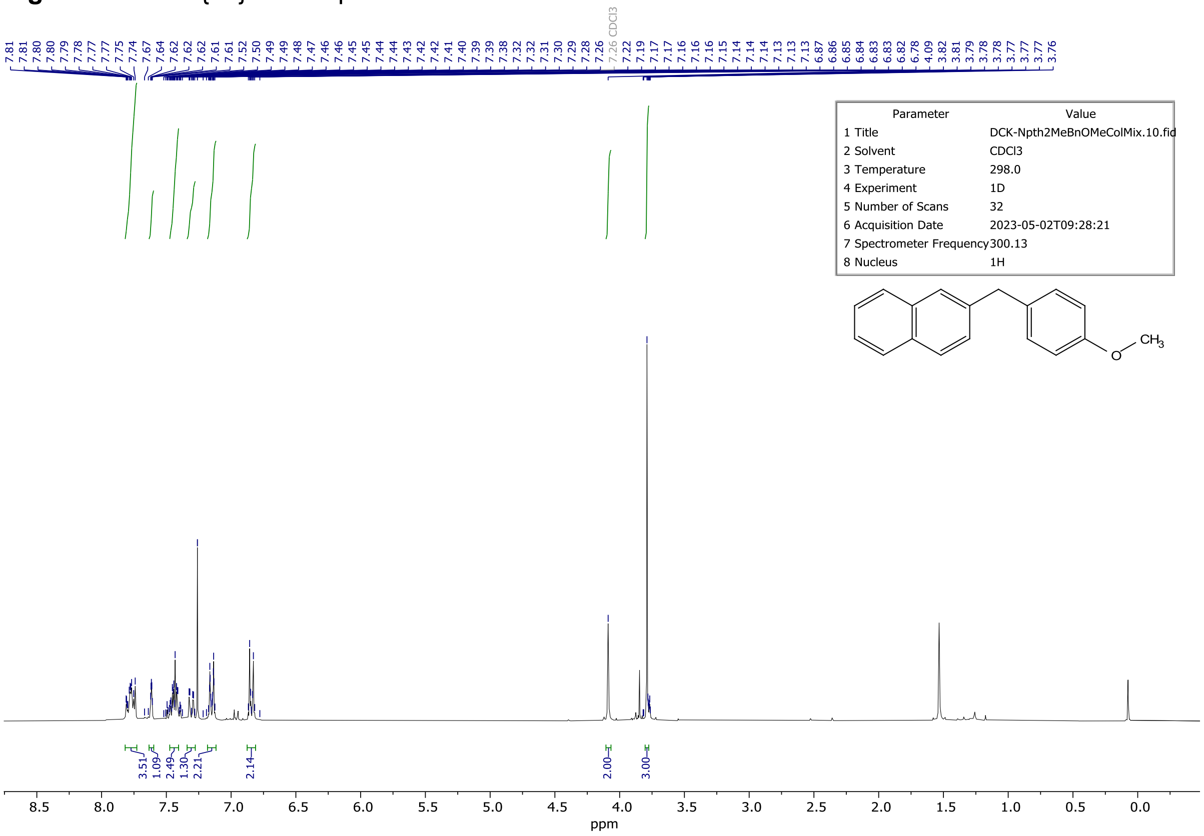

Figure S 43. ^{1}H-NMR spectrum of **4ca** with homocoupling products as minor impurity.

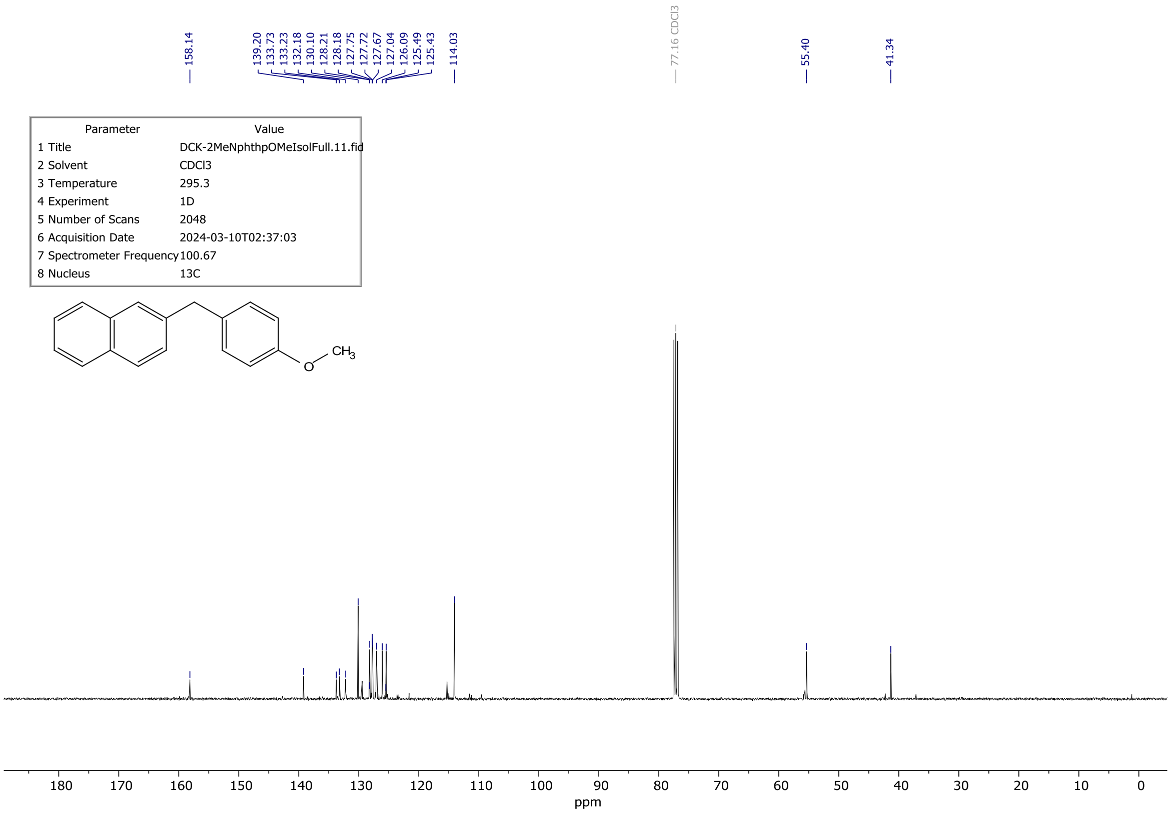

Figure S 44. $^{13}C\{^{1}H\}$-NMR spectrum of **4ca** with homocoupling products as minor impurity.

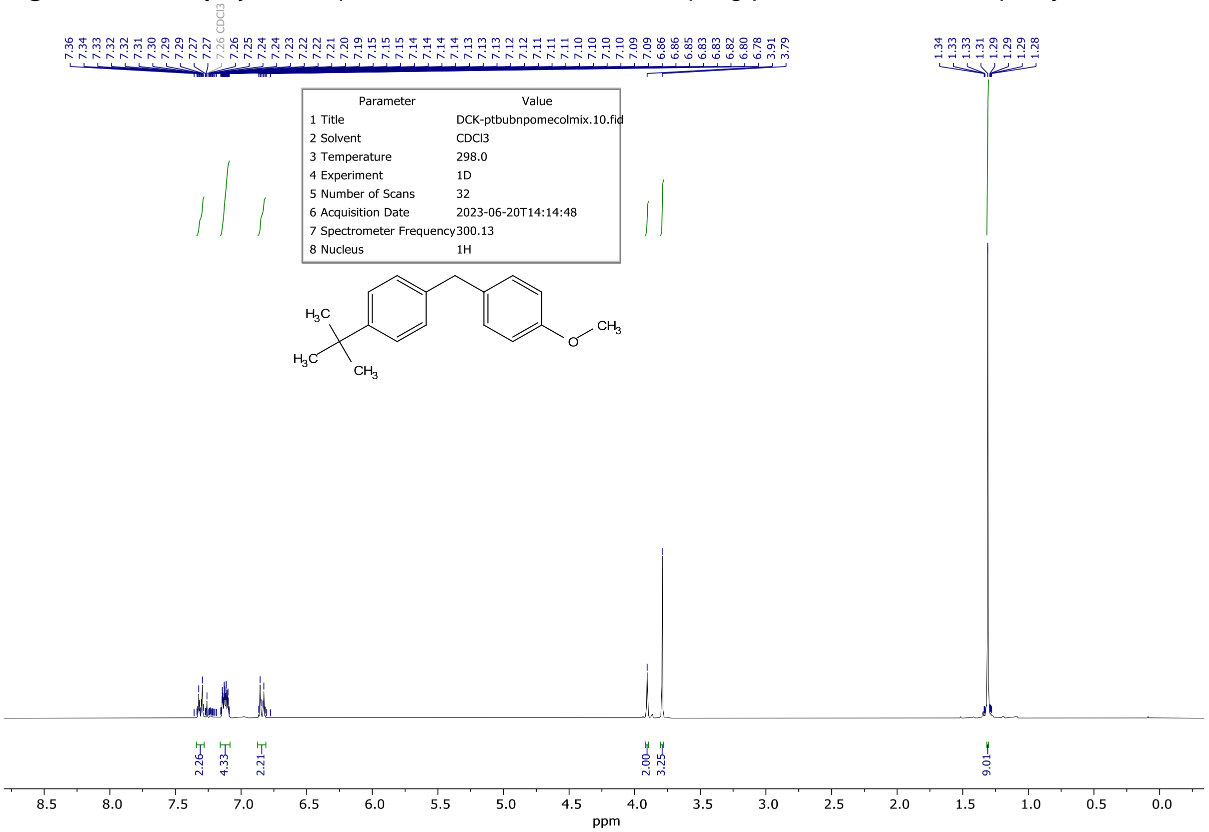

Figure S 45. ^{1}H-NMR spectrum of **4da**.

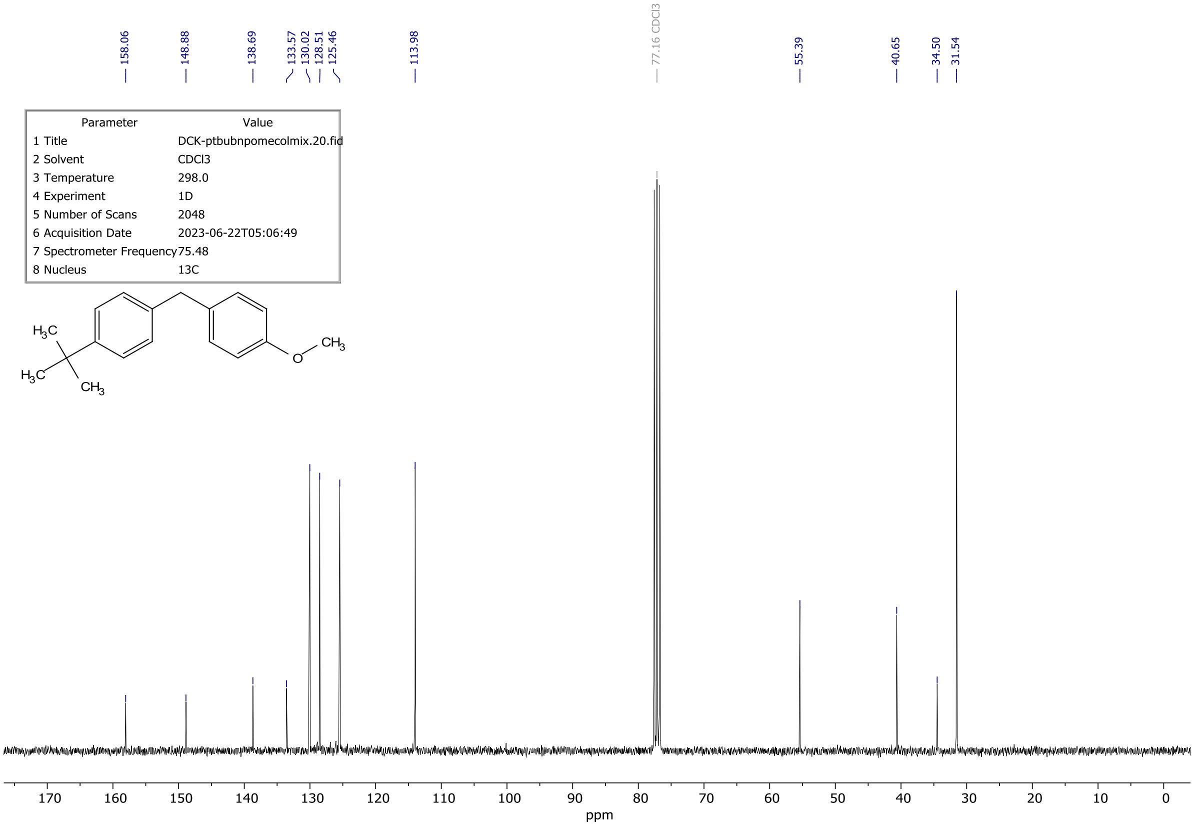

Figure S 46. $^{13}C\{^1H\}$-NMR spectrum of **4da**.

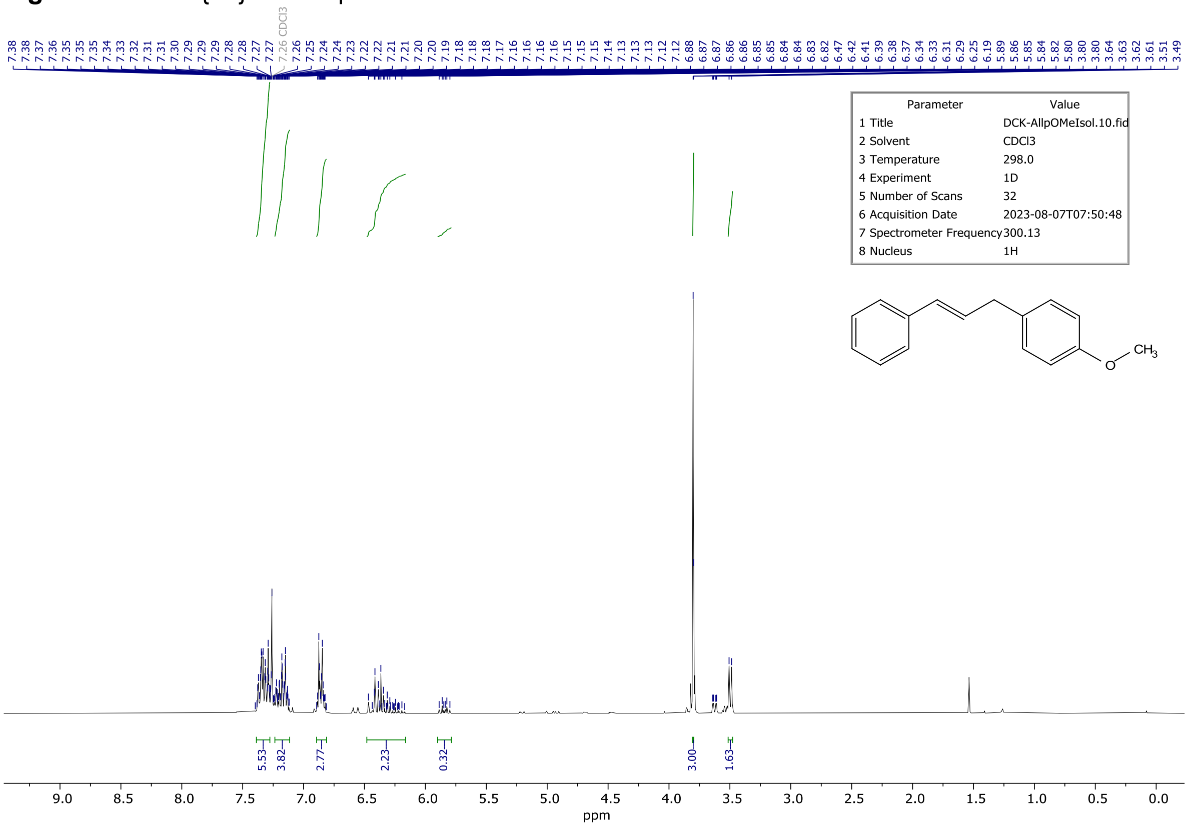

Figure S 47. ^1H-NMR spectrum of **4ja** as a mixture of isomers.

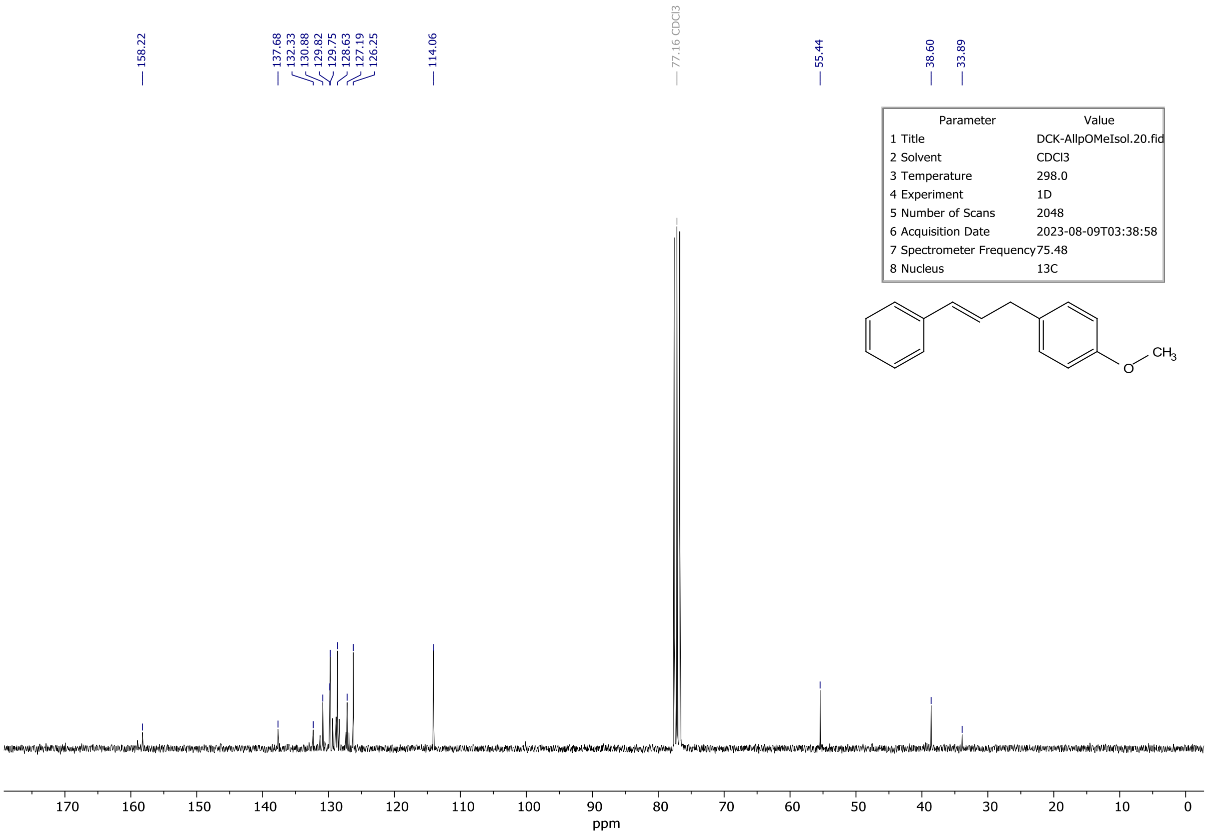

Figure S 48. $^{13}C\{^{1}H\}$-NMR spectrum of **4ja** as a mixture of isomers.

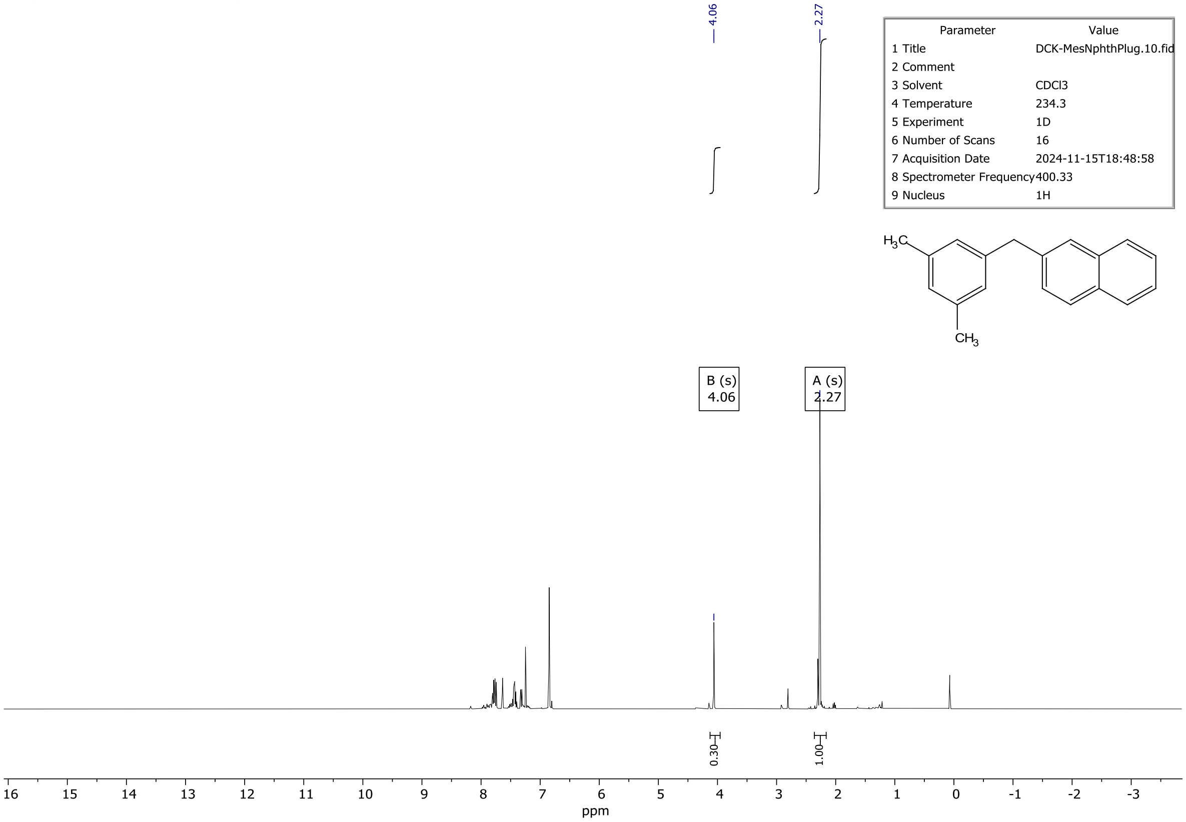

Figure S 49. ^{1}H-NMR spectrum of **4eb**.

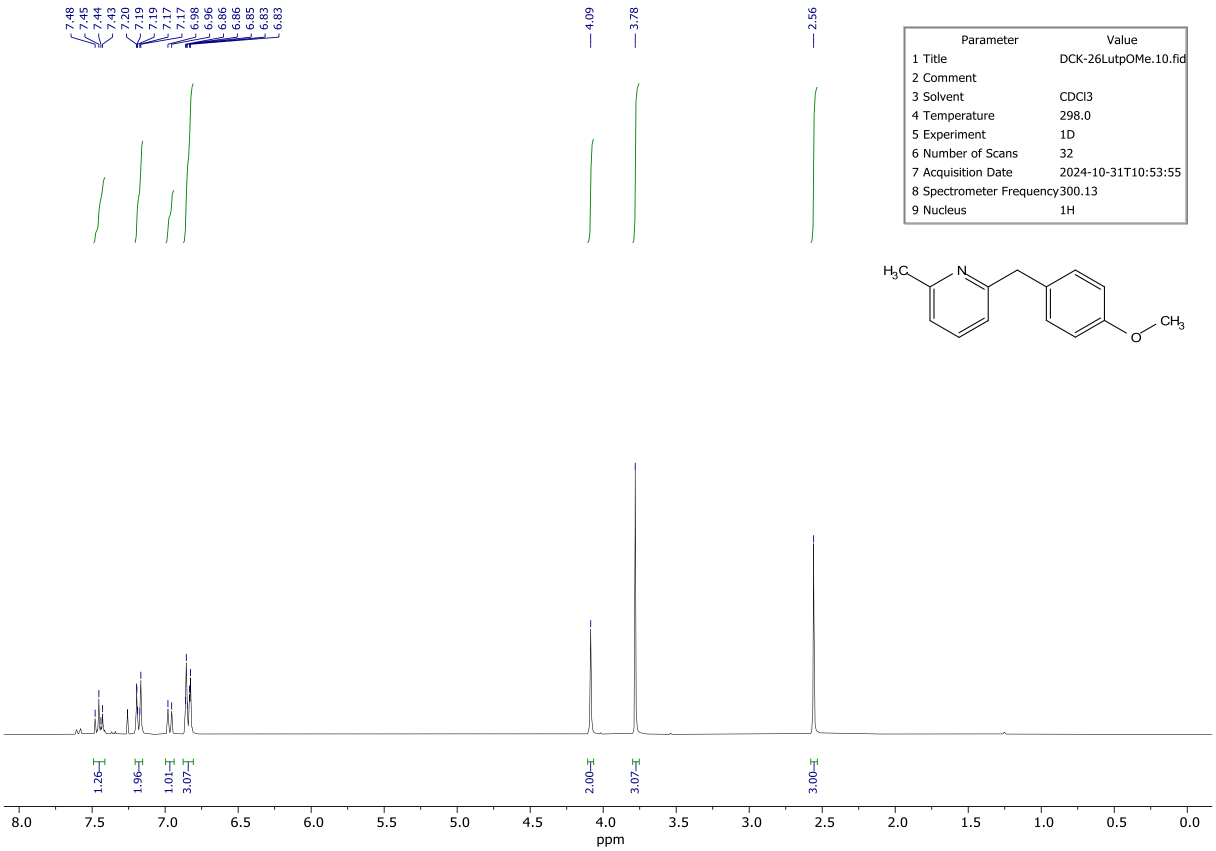

Figure S 50. ^{1}H-NMR spectrum of **4ha.**

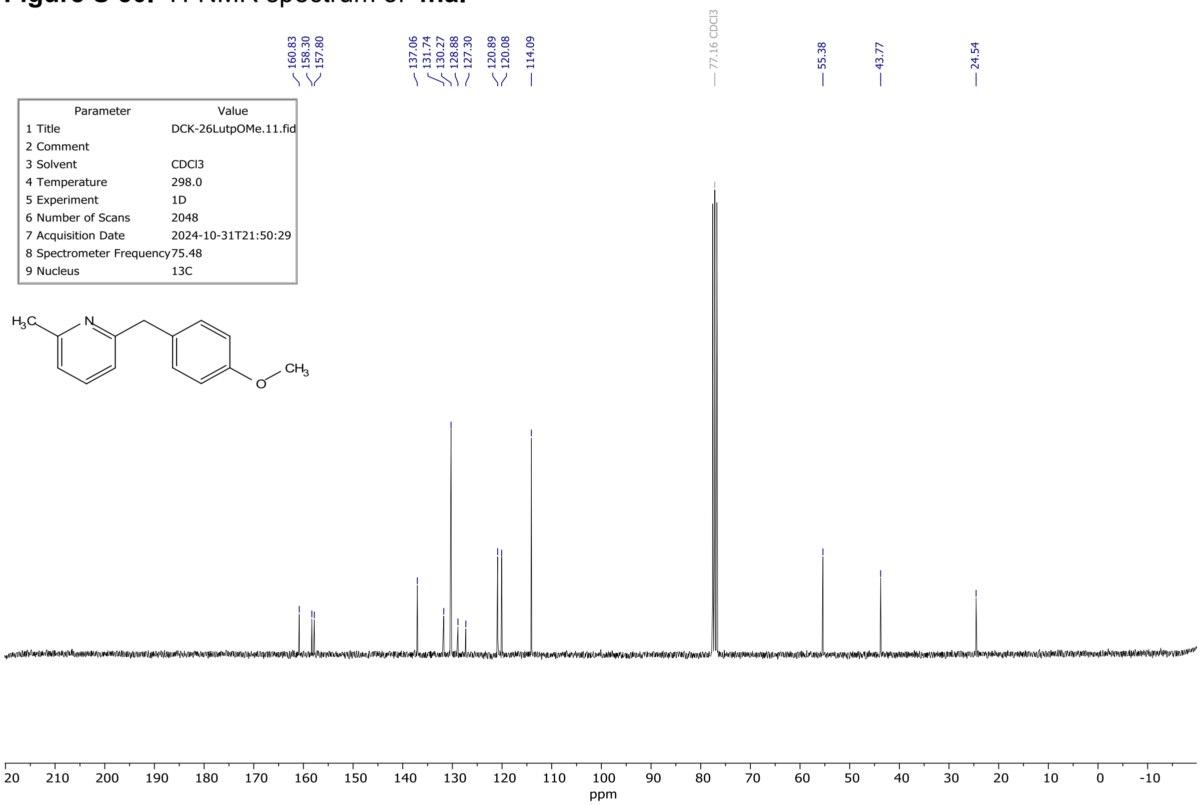

Figure S 51. ^{13}C{^{1}H}-NMR spectrum of **4ha.**

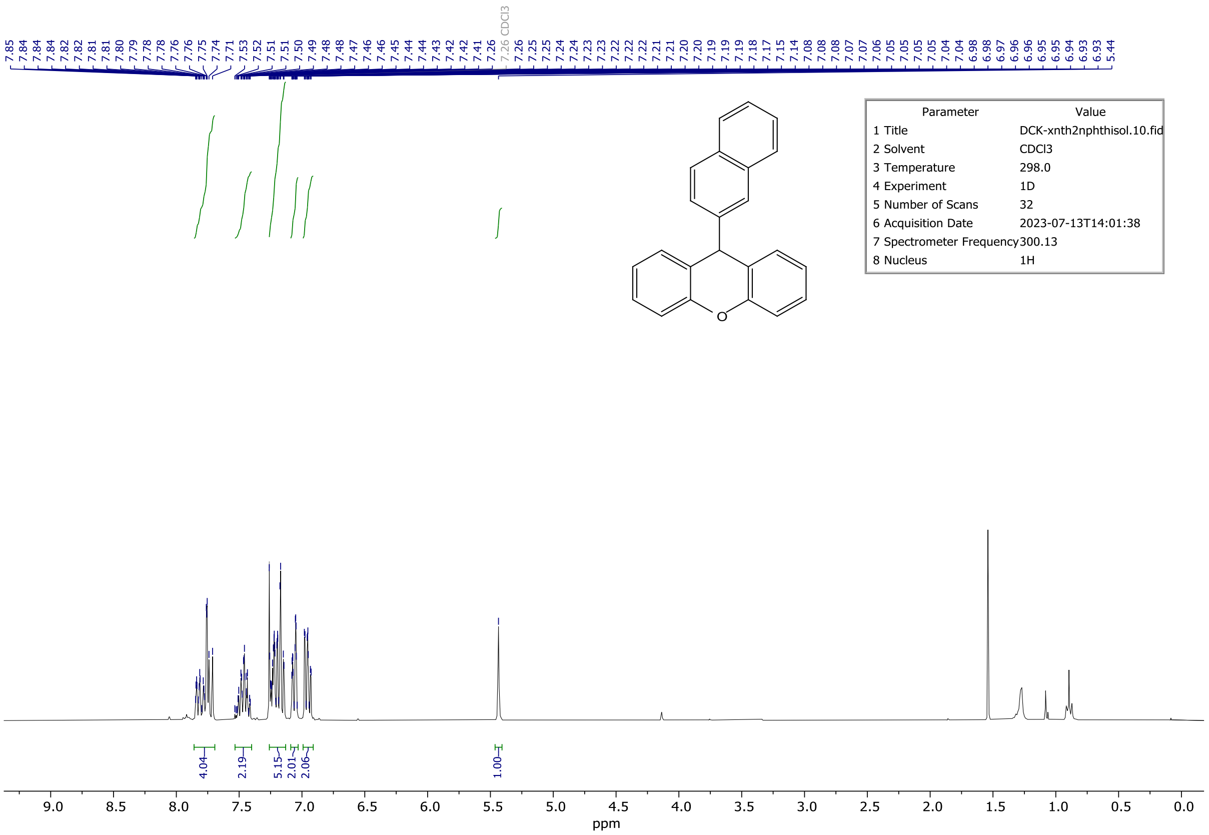

Figure S 52. ^{1}H-NMR spectrum of **4ib**.

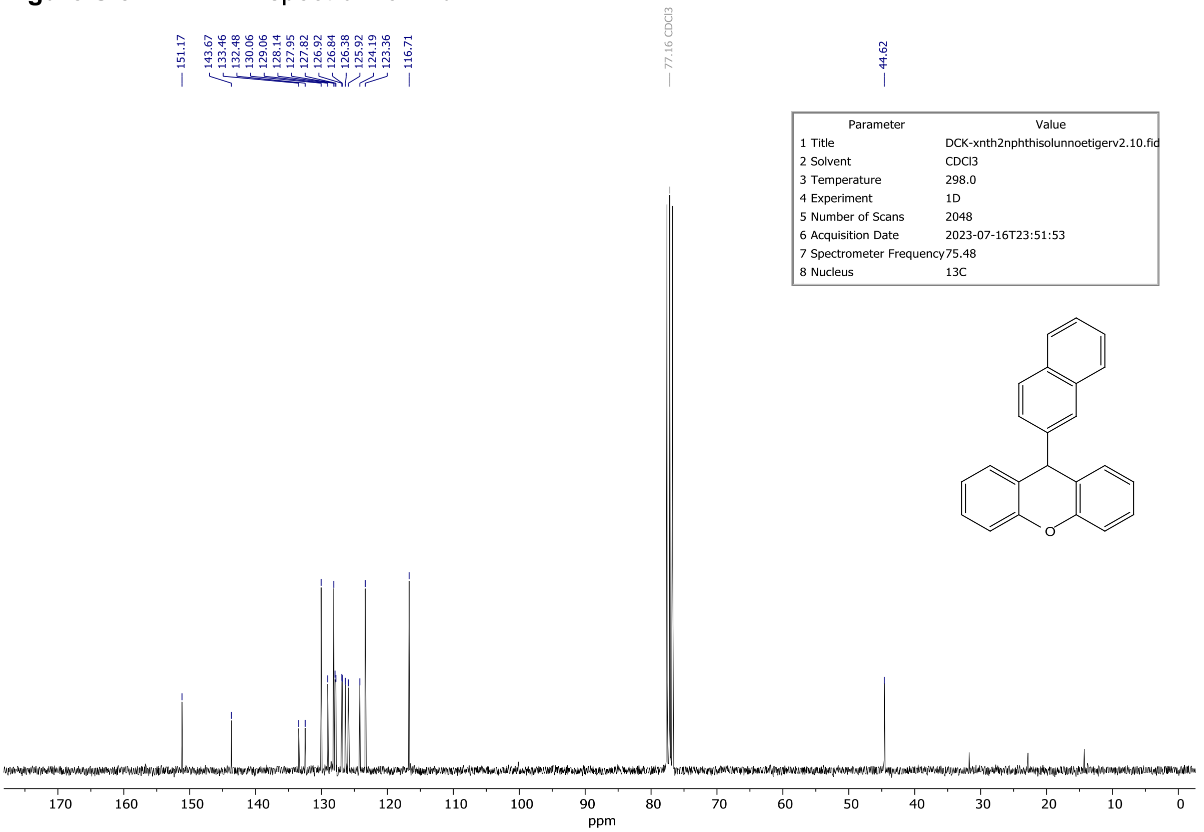

Figure S 53. $^{13}C\{^{1}H\}$-NMR spectrum of **4ib**.

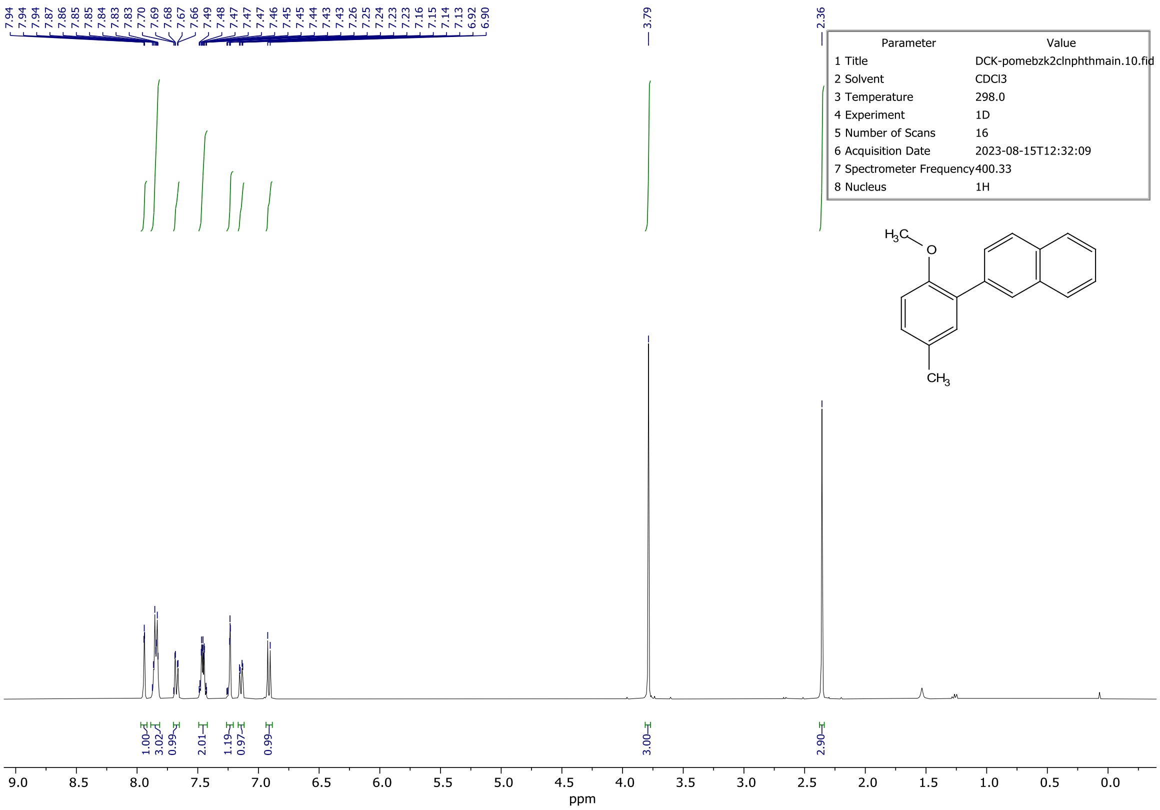

Figure S 54. ^{1}H-NMR spectrum of **4lb**.

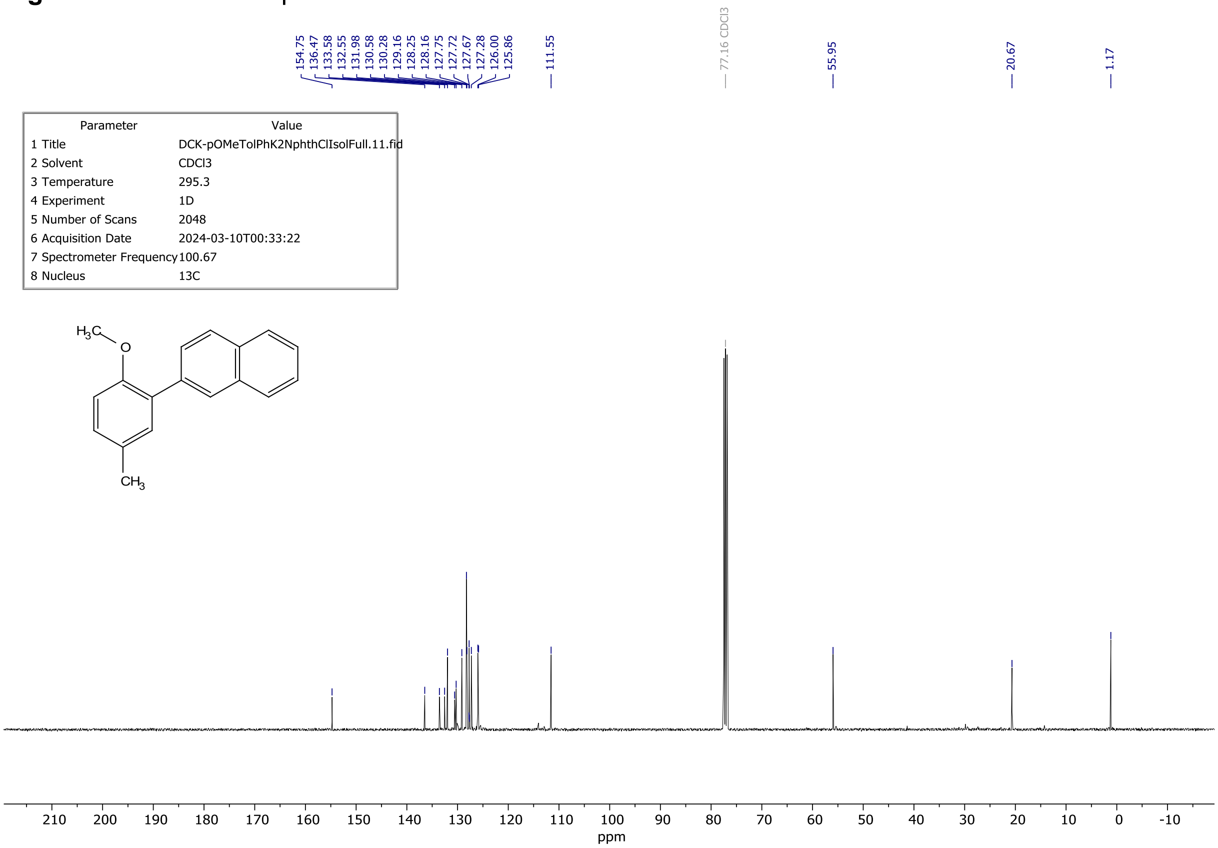

Figure S 55. $^{13}C\{^{1}H\}$-NMR spectrum of **4lb.**

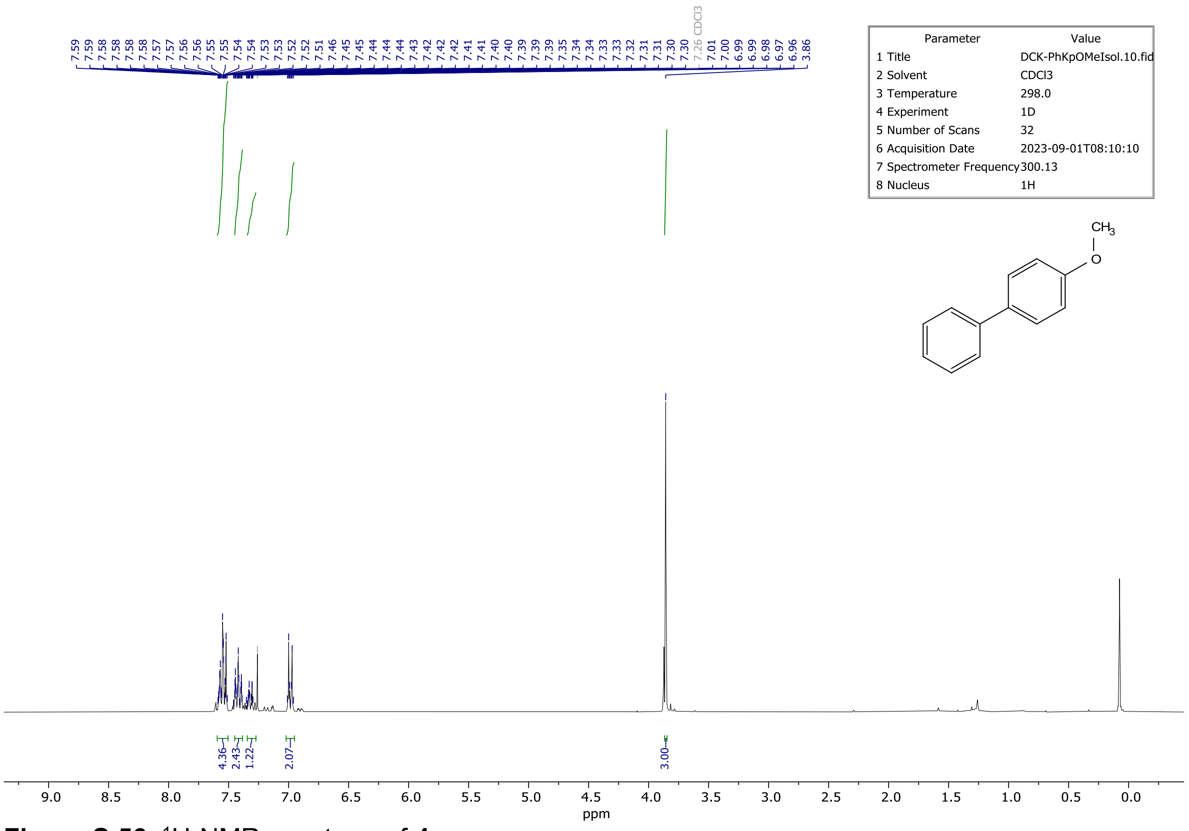

Figure S 56. ^{1}H-NMR spectrum of **4na**.

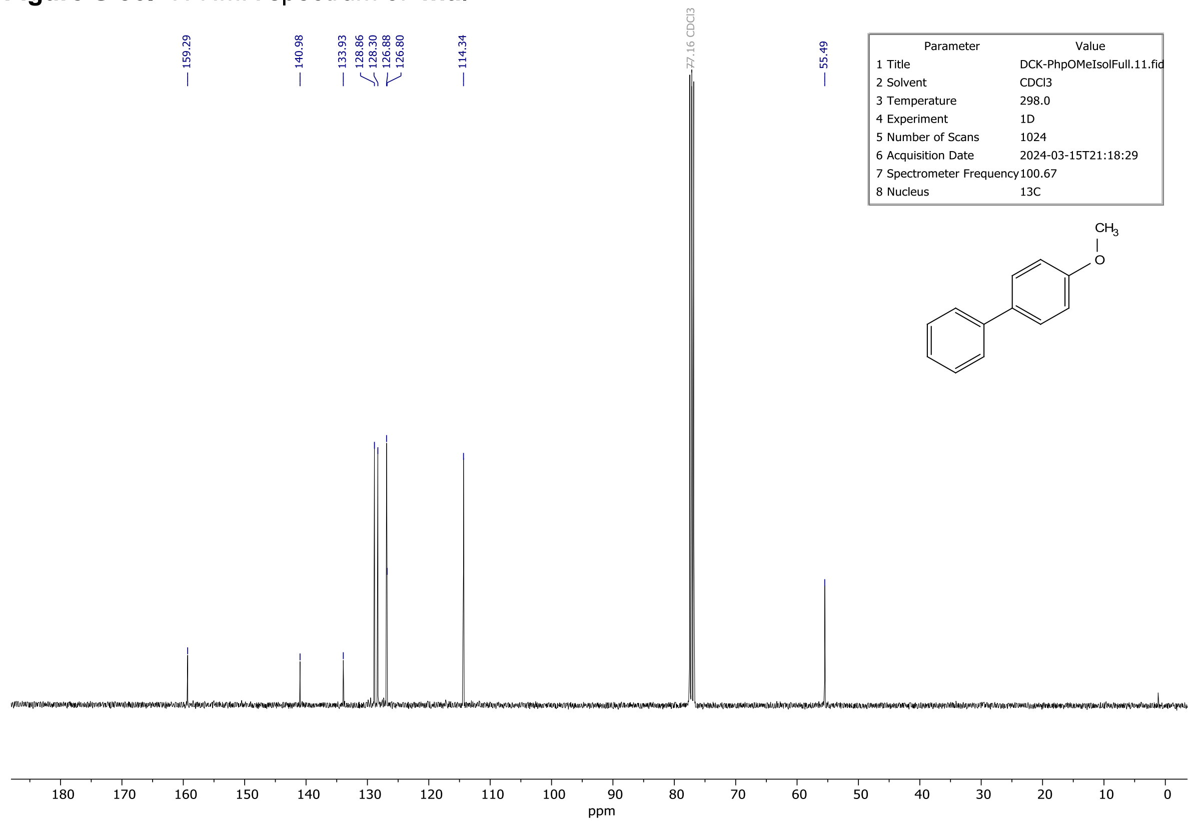

Figure S 57. ^{13}C{^{1}H}-NMR spectrum of **4na**.

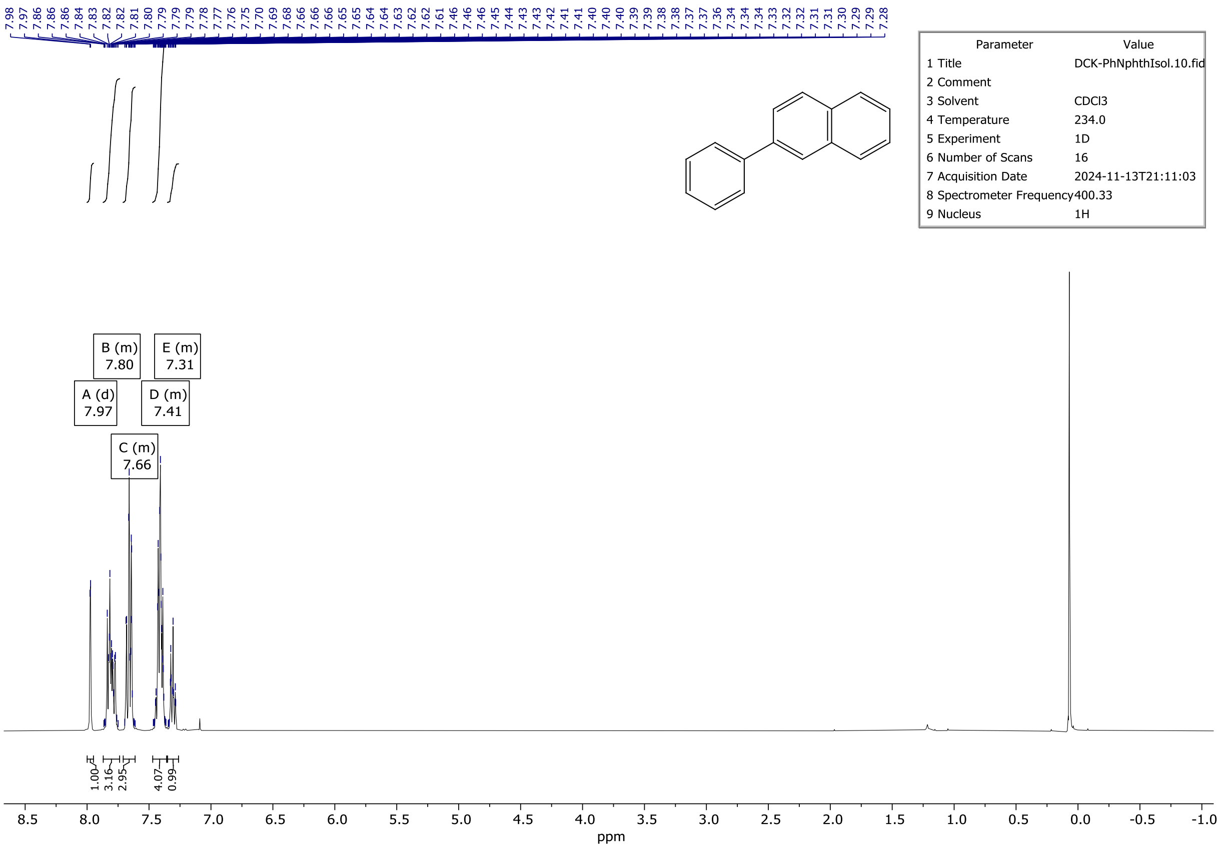

Figure S 58. ^{1}H-NMR spectrum of **4nb**.

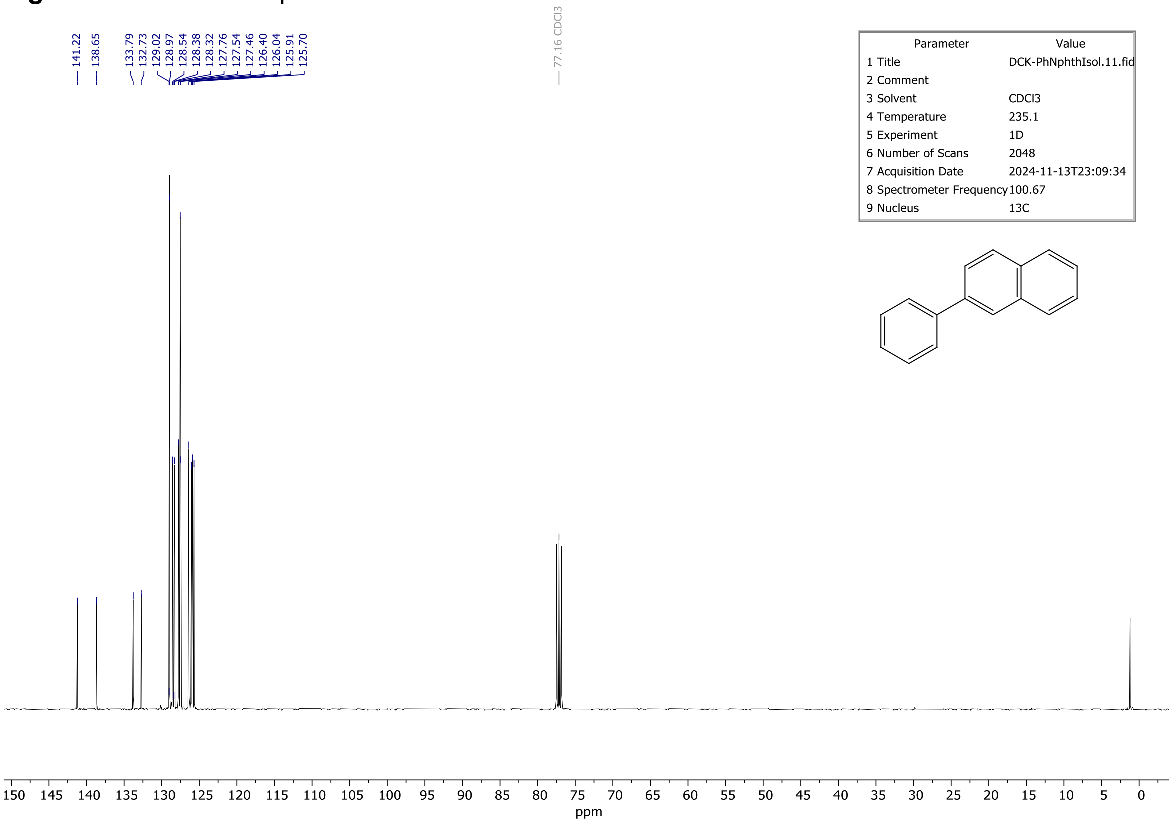

Figure S 59. $^{13}C\{^{1}H\}$-NMR spectrum of **4nb**

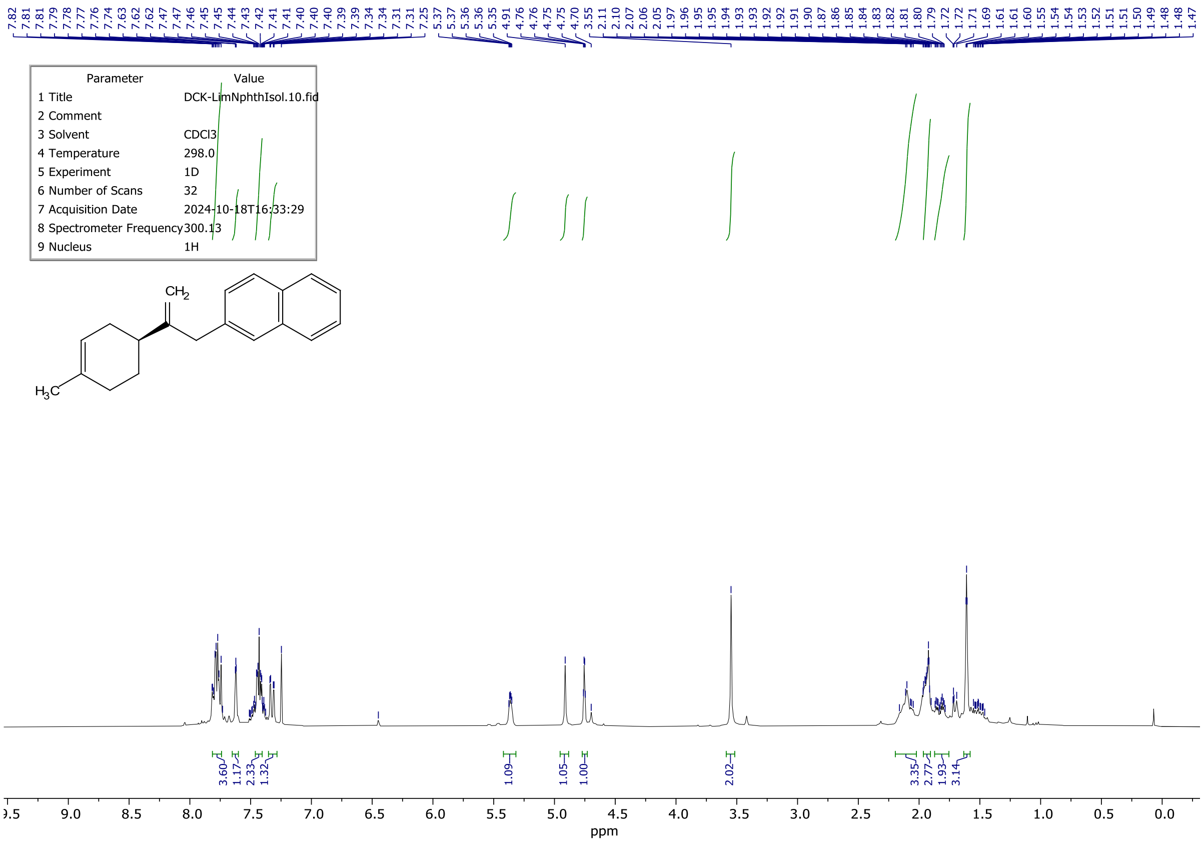

Figure S 60. ^{1}H-NMR spectrum of **4ob** with minor impurities of different isomers.

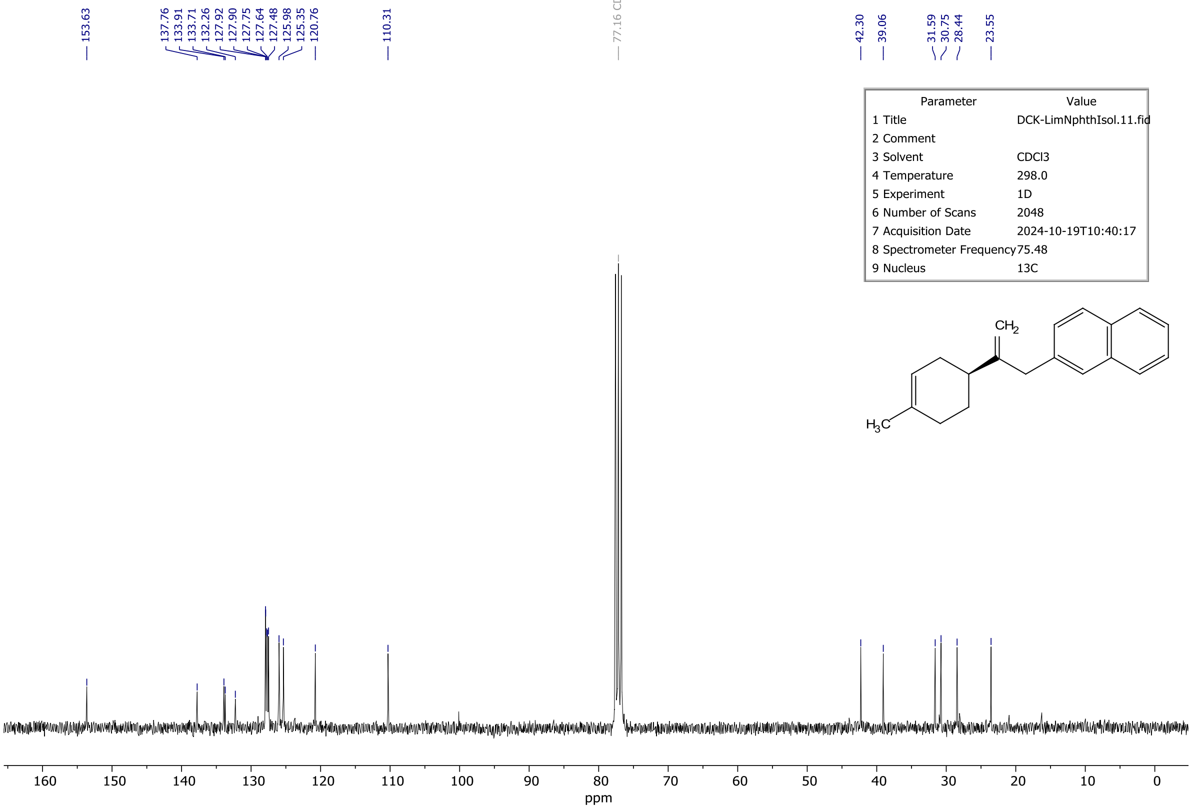

Figure S 61. ^{13}C{^{1}H}-NMR spectrum of **4ob** with minor impurities of different isomers.

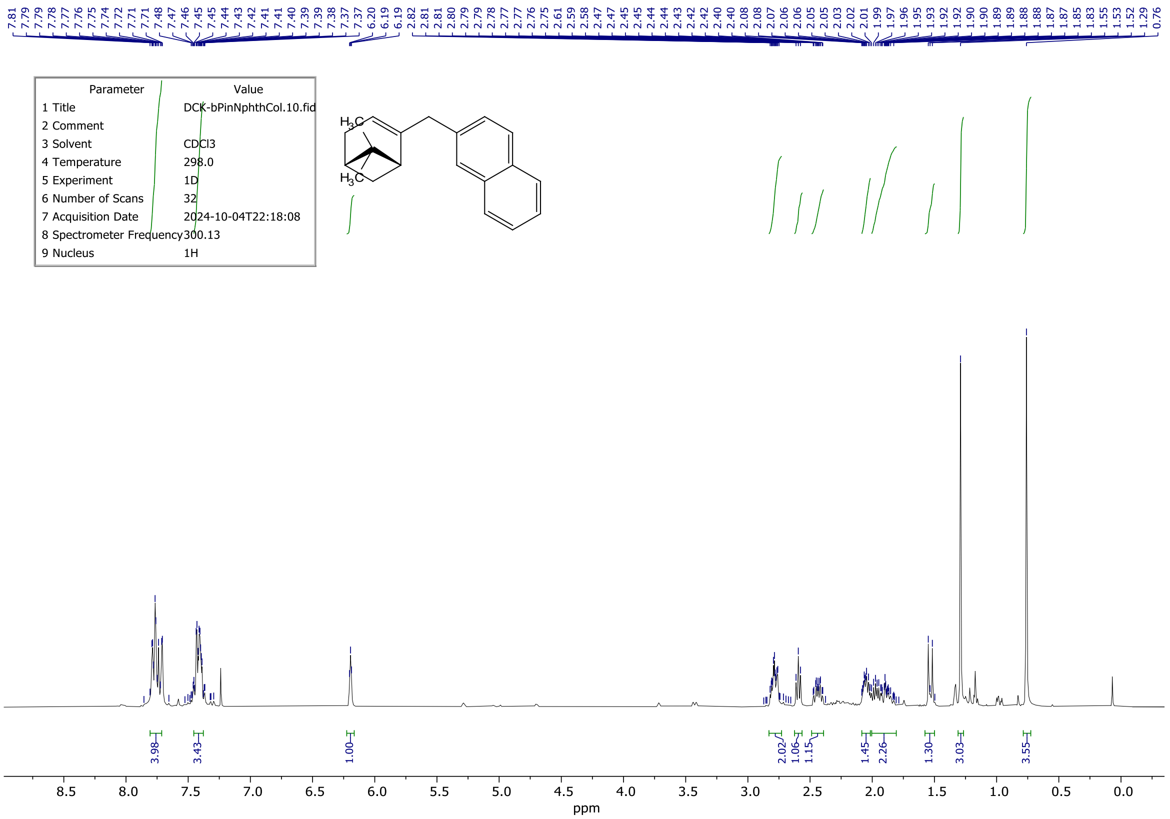

Figure S62. ^{1}H-NMR spectrum of **4pb** with minor impurities of different isomers.

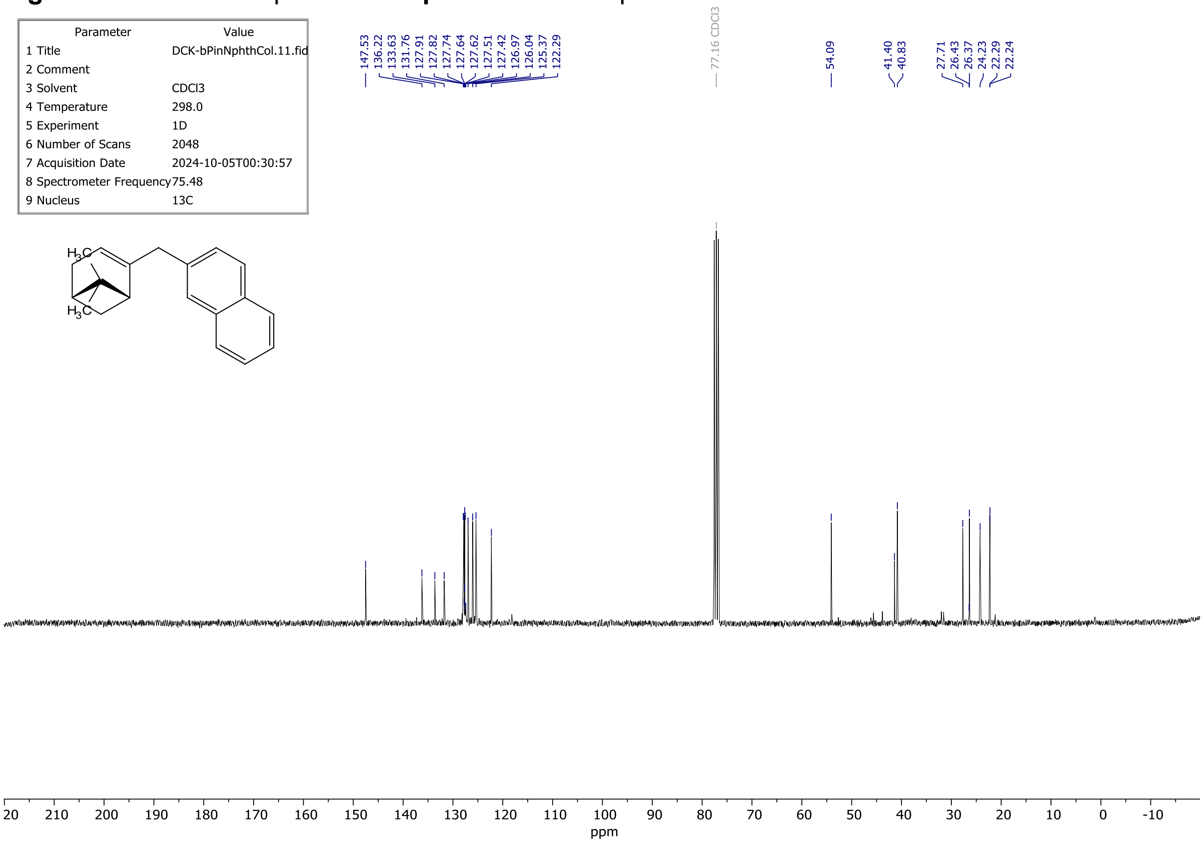

Figure S 63. ^{13}C{^{1}H}-NMR spectrum of **4pb** with minor impurities of different isomers.

3. Xray crystallographic data

Table S7. Crystal data and structure refinement for **2c.**

Empirical formula	C20 H32 K N3	
Formula weight	353.58	
Temperature	173(2) K	
Wavelength l	0.71073 Å (Mo Ka)	
Crystal system	monoclinic	
Space group	P 21/c (no. 14)	
Unit cell dimensions	a = 10.9831(2) Å	a= 90°
	b = 10.76094(18) Å	b= 97.334(2)°
	c = 35.5092(8) Å	g = 90°
Volume	4162.44(15) Å^3	
Z	8	
Density r(calculated)	1.128 g/cm^3	
Absorption coefficient µ	0.261 mm^{-1}	
F(000)	1536	
Crystal size	0.335 x 0.240 x 0.193 mm^3	
Theta range for data collection	2.069 to 30.534°	
Index ranges	-15 <= h <= 15, -15 <= k< = 15, -50 <= l< = 50	
Reflections collected	14097	
Independent reflections	14097 [R_{int} = 0.0783, R_{σ} = 0.0366]	
Completeness to theta = 25.242°	100 %	
Absorption correction	analytical	
Max. and min. transmission	0.961 and 0.94	
Refinement method	full-matrix least-squares on F^2	
Data / restraints / parameters	14097 / 174 / 644	
Goodness-of-fit on F^2	1.014	
Final R indices [I > 2 s(I)]	R1 = 0.0641, wR2 = 0.1617	
R indices (all data)	R1 = 0.0924, wR2 = 0.1725	
Extinction coefficient	n/a	
Largest diff. peak and hole	0.339 and -0.530 e$^-$Å^{-3}	

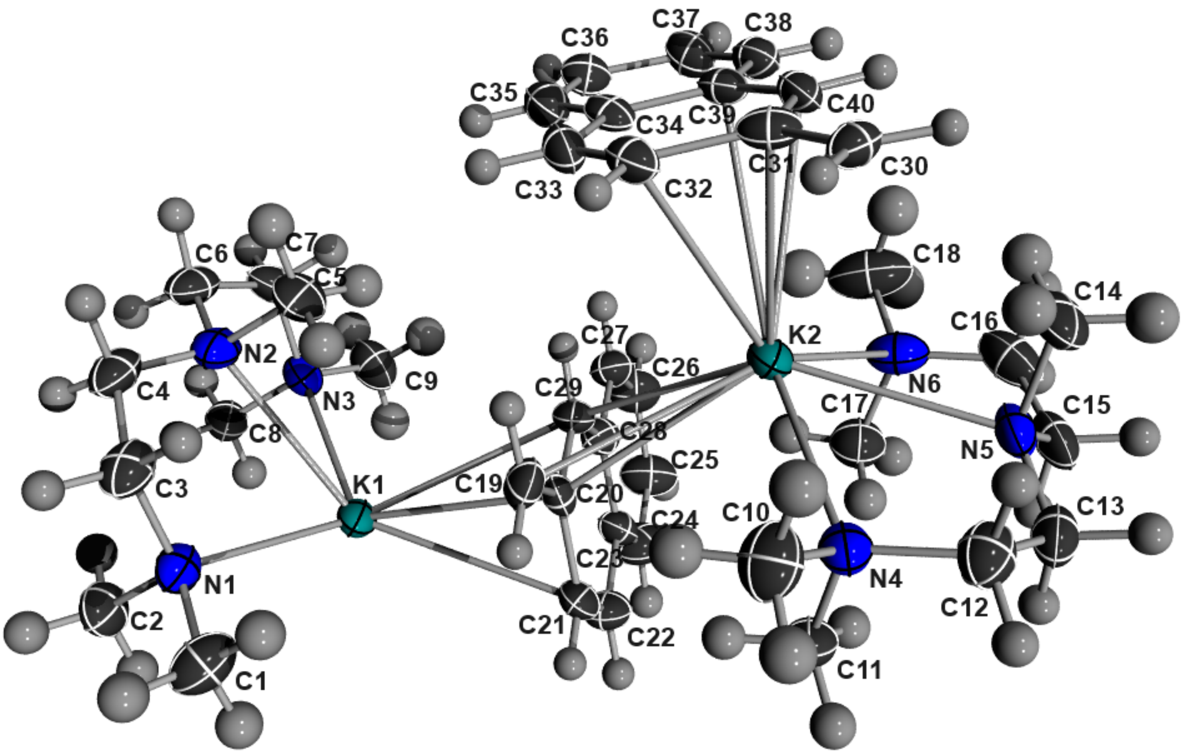

Figure S64. ORTEP of **XXX**. Thermal ellipsoids are drawn at 50 % probability level. Closest contacts are shown at 50 % transparency.

Table S8. Atomic coordinates (x 104) and equivalentisotropic displacement parameters (Å2x 103) for 23eh205.cif. U(eq) is defined as one third of the trace of the orthogonalized Uij tensor for all atoms

atom	x	y	z	U(eq)
K1	0.42357(6)	0.07028(6)	0.35515(2)	0.03336(15)
K2	0.15769(6)	0.56896(6)	0.35755(2)	0.03335(15)
N5	-0.0931(2)	0.6756(2)	0.35017(8)	0.0377(6)
N2	0.6703(2)	0.1780(2)	0.35168(8)	0.0383(6)
N1	0.5315(2)	0.0583(2)	0.28484(7)	0.0336(5)
N4	0.0018(2)	0.5541(2)	0.28566(7)	0.0345(6)
N3	0.6104(2)	0.0521(2)	0.42148(8)	0.0399(6)
N6	0.0197(3)	0.5742(3)	0.42402(8)	0.0485(7)
C13	-0.1698(3)	0.6531(3)	0.31405(10)	0.0432(8)
C30	0.2420(12)	0.8651(11)	0.3105(3)	0.0276(18)
C30A	0.265(3)	0.856(3)	0.3148(9)	0.062(10)
C6	0.7611(3)	0.1577(3)	0.38543(11)	0.0447(8)
C4	0.7231(3)	0.1560(3)	0.31691(10)	0.0414(8)
C5	0.6196(3)	0.3026(3)	0.35197(12)	0.0506(9)
C15	-0.1628(3)	0.6529(3)	0.38214(11)	0.0482(9)
C23	0.1506(16)	0.1618(16)	0.4071(3)	0.033(2)
C20	0.2477(8)	0.2926(8)	0.3436(2)	0.0275(14)
C3	0.6291(3)	0.1491(3)	0.28186(10)	0.0422(8)
C7	0.7058(4)	0.1478(3)	0.42247(10)	0.0483(9)
C31	0.3119(19)	0.8022(18)	0.3389(4)	0.033(2)
C11	-0.0443(3)	0.4288(3)	0.29021(10)	0.0423(7)
C8	0.6615(3)	-0.0701(3)	0.41599(10)	0.0438(7)
C12	-0.0983(3)	0.6452(3)	0.28049(10)	0.0434(8)
C17	-0.0192(4)	0.4446(3)	0.42159(11)	0.0533(9)
C34	0.4657(10)	0.6488(8)	0.3963(2)	0.0301(16)
C39	0.3832(7)	0.7398(6)	0.40690(18)	0.0306(14)
C36	0.5389(12)	0.5924(12)	0.4616(4)	0.037(2)
C10	0.0698(3)	0.5590(4)	0.25269(10)	0.0516(9)
C2	0.5789(3)	-0.0674(3)	0.28813(10)	0.0441(8)
C1	0.4386(3)	0.0674(4)	0.25165(10)	0.0503(9)

C19	0.2880(12)	0.3460(18)	0.3131(5)	0.035(2)
C14	-0.0434(3)	0.8017(3)	0.35152(12)	0.0524(10)
C16	-0.0867(4)	0.6579(3)	0.42044(12)	0.0590(11)
C35	0.5413(6)	0.5786(5)	0.42354(18)	0.0394(13)
C9	0.5561(4)	0.0541(4)	0.45694(11)	0.0602(10)
C40	0.3112(5)	0.8112(5)	0.37964(17)	0.0334(12)
C29	0.2879(6)	0.3149(6)	0.3830(2)	0.0334(13)
C32	0.3999(5)	0.7032(4)	0.33019(16)	0.0317(11)
C18	0.0973(5)	0.5953(5)	0.46048(12)	0.0792(15)
C33	0.4705(4)	0.6359(4)	0.35594(14)	0.0349(11)
C37	0.4499(6)	0.6825(5)	0.47235(19)	0.0431(14)
C38	0.3781(5)	0.7504(4)	0.44710(14)	0.0378(12)
C26	0.2382(8)	0.2186(8)	0.4804(2)	0.0524(18)
C28	0.2442(5)	0.2540(5)	0.41293(17)	0.0349(12)
C27	0.2850(5)	0.2785(5)	0.45184(17)	0.0413(13)
C25	0.1461(11)	0.1273(9)	0.4730(3)	0.056(2)
C21	0.1511(5)	0.1951(4)	0.33794(17)	0.0351(12)
C22	0.1058(5)	0.1364(5)	0.36646(17)	0.0426(14)
C24	0.1041(6)	0.0978(6)	0.4358(2)	0.0550(17)
C20A	0.3325(11)	0.3474(10)	0.3903(4)	0.039(2)
C21A	0.2624(13)	0.2942(13)	0.3582(4)	0.031(3)
C22A	0.1756(8)	0.2035(9)	0.3644(3)	0.046(3)
C23A	0.158(3)	0.157(3)	0.3972(5)	0.031(4)
C24A	0.0714(9)	0.0709(10)	0.4065(4)	0.057(3)
C25A	0.0563(10)	0.0316(10)	0.4421(4)	0.067(4)
C26A	0.130(2)	0.0788(14)	0.4733(7)	0.071(5)
C27A	0.2128(14)	0.1708(13)	0.4692(5)	0.061(4)
C28A	0.2318(9)	0.2135(9)	0.4316(4)	0.045(3)
C29A	0.3151(10)	0.3084(8)	0.4255(4)	0.052(3)
C19A	0.284(3)	0.336(3)	0.3220(9)	0.062(9)
C31A	0.2670(12)	0.8557(11)	0.3815(3)	0.037(3)
C40A	0.3134(10)	0.8084(10)	0.4163(3)	0.046(3)
C39A	0.4026(16)	0.7167(15)	0.4201(4)	0.039(4)
C38A	0.4613(14)	0.6634(14)	0.4552(4)	0.051(4)
C37A	0.542(4)	0.582(4)	0.4562(13)	0.087(13)
C36A	0.5928(14)	0.5403(15)	0.4240(4)	0.058(4)

C35A	0.5452(9)	0.5831(9)	0.3896(3)	0.043(3)
C34A	0.451(2)	0.6716(17)	0.3848(5)	0.032(3)
C33A	0.3942(11)	0.7218(11)	0.3492(4)	0.039(3)
C32A	0.310(4)	0.803(3)	0.3481(7)	0.026(5)

Table S9. Anisotropic displacement parameters(Å^2)for 23eh205.cif. The anisotropic displacement factor exponent takes the form: -2p^2[h^{2}a*^{2}U^{11} + ...+ 2 h k a* b* U^{12}]

atom	U^{11}	U^{22}	U^{33}	U^{23}	U^{13}	U^{12}
K1	0.0292(3)	0.0235(3)	0.0481(4)	-0.0047(3)	0.0081(3)	-0.0013(2)
K2	0.0320(3)	0.0243(3)	0.0441(4)	-0.0039(3)	0.0059(3)	-0.0014(3)
N5	0.0360(14)	0.0233(12)	0.0561(17)	0.0011(11)	0.0147(13)	0.0064(11)
N2	0.0347(14)	0.0240(12)	0.0566(17)	0.0000(11)	0.0069(13)	-0.0040(11)
N1	0.0304(12)	0.0257(12)	0.0456(14)	0.0035(11)	0.0077(11)	0.0019(10)
N4	0.0313(12)	0.0290(12)	0.0442(14)	0.0008(11)	0.0088(11)	-0.0032(11)
N3	0.0411(15)	0.0326(14)	0.0468(16)	-0.0076(12)	0.0086(13)	0.0030(12)
N6	0.0523(17)	0.0502(17)	0.0433(16)	-0.0020(14)	0.0065(14)	-0.0181(15)
C13	0.0313(17)	0.0353(16)	0.063(2)	0.0047(16)	0.0081(16)	0.0066(13)
C30	0.023(4)	0.027(3)	0.032(3)	0.005(2)	-0.001(3)	-0.005(3)
C30A	0.043(15)	0.046(10)	0.09(2)	-0.026(12)	-0.001(11)	0.020(8)
C6	0.0368(18)	0.0313(16)	0.064(2)	0.0003(15)	-0.0005(17)	-0.0093(14)
C4	0.0293(16)	0.0350(16)	0.061(2)	0.0051(15)	0.0087(15)	-0.0056(13)
C5	0.051(2)	0.0244(15)	0.077(3)	-0.0052(16)	0.009(2)	0.0013(15)
C15	0.051(2)	0.0321(16)	0.066(2)	-0.0062(16)	0.0250(19)	0.0079(15)
C23	0.038(4)	0.028(3)	0.031(5)	-0.005(4)	0.001(4)	-0.001(3)
C20	0.031(3)	0.027(3)	0.024(4)	-0.004(3)	0.002(3)	0.010(2)
C3	0.0384(18)	0.0339(16)	0.056(2)	0.0094(15)	0.0136(16)	-0.0037(13)
C7	0.061(2)	0.0308(16)	0.050(2)	-0.0096(15)	-0.0041(18)	-0.0017(16)
C31	0.039(4)	0.032(4)	0.029(5)	0.002(4)	0.005(4)	-0.015(3)
C11	0.0356(16)	0.0352(15)	0.056(2)	-0.0057(16)	0.0039(14)	-0.0055(15)
C8	0.0422(17)	0.0339(15)	0.057(2)	-0.0034(16)	0.0123(16)	-0.0025(15)
C12	0.0391(18)	0.0397(17)	0.051(2)	0.0091(15)	0.0058(16)	0.0028(14)

C17	0.055(2)	0.049(2)	0.056(2)	0.0018(17)	0.0081(19)	-0.0054(18)
C34	0.041(4)	0.020(3)	0.032(4)	0.001(2)	0.012(3)	-0.010(3)
C39	0.036(3)	0.027(3)	0.030(3)	-0.007(2)	0.008(3)	-0.011(2)
C36	0.046(5)	0.031(4)	0.035(4)	0.006(3)	0.003(3)	-0.004(3)
C10	0.0458(19)	0.058(2)	0.053(2)	0.0070(18)	0.0159(16)	0.0034(19)
C2	0.0426(18)	0.0309(15)	0.059(2)	-0.0016(16)	0.0094(15)	0.0076(16)
C1	0.0401(17)	0.054(2)	0.055(2)	0.0127(18)	0.0024(15)	-0.0058(19)
C19	0.023(4)	0.039(4)	0.045(6)	-0.001(4)	0.011(4)	0.002(3)
C14	0.056(2)	0.0236(15)	0.080(3)	0.0023(16)	0.019(2)	-0.0015(15)
C16	0.082(3)	0.0413(19)	0.060(2)	-0.0096(18)	0.031(2)	-0.004(2)
C35	0.044(3)	0.032(3)	0.043(3)	-0.002(2)	0.010(3)	-0.001(2)
C9	0.073(3)	0.057(2)	0.054(2)	-0.0096(19)	0.018(2)	0.005(2)
C40	0.034(3)	0.022(3)	0.047(3)	-0.009(2)	0.014(2)	-0.003(2)
C29	0.028(3)	0.026(3)	0.046(4)	-0.009(2)	0.002(3)	-0.005(2)
C32	0.039(3)	0.026(2)	0.032(3)	-0.005(2)	0.013(2)	-0.0070(18)
C18	0.089(3)	0.098(4)	0.050(2)	-0.003(2)	0.007(2)	-0.045(3)
C33	0.036(2)	0.026(2)	0.046(3)	-0.0035(19)	0.018(2)	0.0000(18)
C37	0.055(3)	0.040(3)	0.035(3)	-0.005(2)	0.010(3)	-0.002(2)
C38	0.040(3)	0.035(2)	0.040(3)	-0.009(2)	0.010(2)	-0.006(2)
C26	0.061(4)	0.055(4)	0.039(3)	0.006(3)	0.000(3)	-0.007(4)
C28	0.032(3)	0.025(3)	0.046(3)	0.000(2)	-0.002(2)	0.001(2)
C27	0.037(3)	0.040(3)	0.045(3)	-0.004(2)	-0.002(2)	-0.005(2)
C25	0.071(5)	0.050(5)	0.044(4)	0.007(4)	-0.001(4)	-0.015(5)
C21	0.034(2)	0.024(2)	0.047(3)	-0.010(2)	0.002(2)	0.0008(19)
C22	0.039(3)	0.036(3)	0.053(3)	-0.005(2)	0.002(2)	-0.009(2)
C24	0.053(4)	0.046(3)	0.066(4)	0.003(3)	0.006(3)	-0.013(3)
C20A	0.037(5)	0.021(4)	0.056(6)	0.002(4)	-0.006(5)	-0.004(4)
C21A	0.040(5)	0.019(4)	0.030(6)	-0.001(5)	-0.012(6)	0.009(3)
C22A	0.028(4)	0.042(5)	0.063(6)	-0.028(4)	-0.017(4)	0.013(4)
C23A	0.034(5)	0.033(5)	0.028(8)	-0.009(7)	0.007(7)	0.000(4)
C24A	0.039(5)	0.044(5)	0.082(7)	-0.008(5)	-0.011(5)	0.003(4)
C25A	0.037(6)	0.044(6)	0.116(12)	-0.002(6)	-0.006(6)	-0.019(5)
C26A	0.072(10)	0.041(8)	0.091(11)	0.015(9)	-0.018(8)	-0.012(9)
C27A	0.054(8)	0.039(7)	0.084(11)	0.009(7)	-0.018(8)	-0.014(6)
C28A	0.040(5)	0.022(4)	0.070(8)	0.003(5)	-0.003(5)	-0.009(4)
C29A	0.051(6)	0.028(4)	0.072(8)	0.000(5)	-0.012(6)	-0.009(4)

C19A	0.072(15)	0.051(15)	0.056(18)	-0.024(12)	-0.018(11)	0.022(11)
C31A	0.045(7)	0.025(6)	0.041(6)	-0.001(5)	0.007(5)	-0.003(5)
C40A	0.047(5)	0.040(5)	0.058(6)	-0.015(5)	0.032(5)	0.002(4)
C39A	0.040(6)	0.038(6)	0.038(7)	0.006(5)	0.004(6)	-0.003(5)
C38A	0.061(8)	0.058(8)	0.038(7)	-0.014(7)	0.024(7)	-0.017(7)
C37A	0.10(2)	0.076(19)	0.08(2)	-0.016(14)	-0.025(16)	-0.019(15)
C36A	0.053(9)	0.071(10)	0.051(8)	0.006(7)	0.013(7)	0.019(7)
C35A	0.041(5)	0.030(5)	0.062(7)	0.000(5)	0.020(5)	0.000(4)
C34A	0.042(7)	0.024(7)	0.031(9)	0.009(5)	0.010(7)	-0.007(5)
C33A	0.043(6)	0.040(6)	0.034(6)	-0.018(5)	0.004(5)	-0.020(5)
C32A	0.032(6)	0.014(5)	0.032(10)	0.005(7)	0.002(8)	-0.010(4)

4. Contributions

V.H.G. and E.H. designed and supervised the project. J.L. discovered the reported reactivity. D.K. performed all metalation experiments, the screening of the catalysis conditions and the isolation of the cross-coupling products. D.A. performed crystallizations of potassium organyl 2c. The manuscript was written by D.K. and V.H.G.

5. References

1 P. Weber, T. Scherpf, I. Rodstein, D. Lichte, L. T. Scharf, L. J. Gooßen and V. H. Gessner, *Angew. Chem. Int. Ed.* 2019, **58**, 3203-3207.

2 J. Tappen, I. Rodstein, K. McGuire, A. Großjohann, J. Löffler, T. Scherpf and V. H. Gessner, *Chem. Eur. J.* 2020, **26**, 4281- 4288.

3 I. Rodstein, D.S. Prendes, L. Wickert, M. Paaßen and V.H. Gessner, *J. Org. Chem.* 2020, **85**, 14674-14683.

4 S. Bachmann, B. Gernert, D. Stalke, *Chem Commun.* **2016**, *52*, 12861-12864
